# Axicabtagene ciloleucel as first-line therapy in high-risk large B-cell lymphoma: the phase 2 ZUMA-12 trial

**DOI:** 10.1038/s41591-022-01731-4

**Published:** 2022-03-21

**Authors:** Sattva S. Neelapu, Michael Dickinson, Javier Munoz, Matthew L. Ulrickson, Catherine Thieblemont, Olalekan O. Oluwole, Alex F. Herrera, Chaitra S. Ujjani, Yi Lin, Peter A. Riedell, Natasha Kekre, Sven de Vos, Christine Lui, Francesca Milletti, Jinghui Dong, Hairong Xu, Julio C. Chavez

**Affiliations:** 1 The University of Texas MD Anderson Cancer Center, Houston, TX USA; 2 Peter MacCallum Cancer Centre, Royal Melbourne Hospital and The University of Melbourne, Melbourne, Victoria Australia; 3 Banner MD Anderson Cancer Center, Gilbert, AZ USA; 4 Université de Paris, AP-HP, Hôpital Saint-Louis, Hemato-oncology, DMU HI, Paris, France; 5 Research Unit NF-kappaB, Différenciation et Cancer, Paris, France; 6 Vanderbilt-Ingram Cancer Center, Nashville, TN USA; 7 City of Hope National Medical Center, Duarte, CA USA; 8 Seattle Cancer Care Alliance, Fred Hutchinson Cancer Research Center, Seattle, WA USA; 9 Mayo Clinic, Rochester, MN USA; 10 University of Chicago Medicine, Chicago, IL USA; 11 Department of Medicine, The Ottawa Hospital, Ottawa, Ontario Canada; 12 David Geffen School of Medicine at UCLA, Santa Monica, CA USA; 13 Kite, a Gilead Company, Santa Monica, CA USA; 14 Moffitt Cancer Center, Tampa, FL USA

**Keywords:** Phase II trials, Cancer immunotherapy, B-cell lymphoma

## Abstract

High-risk large B-cell lymphoma (LBCL) has poor outcomes with standard first-line chemoimmunotherapy. In the phase 2, multicenter, single-arm ZUMA-12 study (ClinicalTrials.gov NCT03761056) we evaluated axicabtagene ciloleucel (axi-cel), an autologous anti-CD19 chimeric antigen receptor (CAR) T-cell therapy, as part of first-line treatment in 40 patients with high-risk LBCL. This trial has completed accrual. The primary outcome was complete response rate (CRR). Secondary outcomes were objective response rate (ORR), duration of response (DOR), event-free survival (EFS), progression-free survival (PFS), overall survival (OS), assessment of safety, central nervous system (CNS) relapse and blood levels of CAR T cells and cytokines. The primary endpoint in efficacy-evaluable patients (*n* = 37) was met, with 78% CRR (95% confidence interval (CI), 62–90) and 89% ORR (95% CI, 75–97). As of 17 May 2021 (median follow-up, 15.9 months), 73% of patients remained in objective response; median DOR, EFS and PFS were not reached. Grade ≥3 cytokine release syndrome (CRS) and neurologic events occurred in three patients (8%) and nine patients (23%), respectively. There were no treatment-related grade 5 events. Robust CAR T-cell expansion occurred in all patients with a median time to peak of 8 days. We conclude that axi-cel is highly effective as part of first-line therapy for high-risk LBCL, with a manageable safety profile.

## Abstract (web summary)

In a phase 2 trial, first-line treatment with axicabtagene ciloleucel, an autologous CD19-targeting CAR T-cell therapy, exhibited a high complete response rate and a manageable safety profile in adults with high-risk large B-cell lymphoma.

## Main

Large B-cell lymphoma accounts for up to 40% of all new diagnoses among non-Hodgkin lymphomas worldwide, making it the most common subtype^1^. While about 60% of patients with LBCL have durable responses to standard first-line chemoimmunotherapy regimens—such as six cycles of rituximab, cyclophosphamide, doxorubicin, vincristine and prednisone (R-CHOP) or dose-adjusted etoposide, prednisone, vincristine, cyclophosphamide, doxorubicin and rituximab (DA-EPOCH-R)—outcomes are lower in patients with high-risk LBCL^2^. For instance, patients with LBCL and an International Prognostic Index (IPI) score of 3–5 (high–intermediate- and high-risk) have lower EFS, PFS and OS with standard rituximab-containing chemoimmunotherapy regimens^3,4^. Intensification of chemoimmunotherapy and/or first-line consolidation with high-dose chemotherapy and autologous stem cell transplantation did not improve outcomes in patients with high IPI^2^. A number of recent studies have also assessed early positron emission tomography–computed tomography (PET–CT) using visual analysis by Deauville criteria as a measure of dynamic risk assessment to therapy^5–9^. Based on the results of these studies, patients with LBCL who had a positive PET result (PET2^+^) after two cycles of standard first-line chemoimmunotherapy appeared to have a worse prognosis compared with patients who had a negative PET2 (PET2^–^).

Additionally, patients with high-grade B-cell lymphoma (HGBL) characterized by gene rearrangements of *MYC* and *BCL2* and/or *BCL6* (that is, double- or triple-hit lymphomas) have a poor prognosis with standard first R-CHOP, with CRR <50% (refs. ^10–12^). These high-risk patients are also more likely to have CNS involvement^13^. While DA-EPOCH-R is widely used in double- or triple-hit lymphomas, CRRs remain <60% and there is no clear survival benefit with the more aggressive induction regimen compared with standard R-CHOP^14,15^. At present, optimal therapy for double- or triple-hit lymphomas or patients with LBCL and high IPI is unclear. Given the poor outcomes collectively observed, these patients have a high need for more effective therapeutic options early in their disease course.

Chimeric antigen receptor T-cell therapies targeting CD19 have demonstrated manageable safety profiles and high efficacy in the treatment of relapsed/refractory B-cell lymphomas, including LBCL, mantle cell lymphoma and follicular lymphoma^16–20^. Axi-cel, an autologous anti-CD19 CAR T-cell therapy, is approved for patients with relapsed/refractory LBCL after two or more systemic lines of therapy^21,22^. Initial approval of axi-cel in relapsed/refractory LBCL was based on ZUMA-1, a single-arm, multicenter trial of 101 patients with refractory LBCL after two or more lines of systemic therapy^23^. After 63 months of median follow-up, median OS in ZUMA-1 was 25.8 months and the 5-year OS rate was 43%, demonstrating long-term disease control with axi-cel in patients with refractory LBCL^24^. In ZUMA-1, patients who were less heavily pretreated appeared to have product CAR T cells that were potentially more biologically and clinically active^25^.

Although CAR T-cell therapy is established for the treatment of relapsed/refractory B-cell lymphomas, its potential when applied as part of first-line therapy for patients at risk of early chemotherapy failure has not been studied. ZUMA-12, a prospective, phase 2, multicenter, single-arm trial, assessed the efficacy and safety of axi-cel as part of first-line therapy after an incomplete first-line treatment regimen of two cycles of chemoimmunotherapy. High-risk LBCL was defined by the dynamic risk assessment of interim PET2^+^, together with either double- or triple-hit lymphomas or high–intermediate- and high-risk IPI scores (≥3). Here, we report the results of the primary analysis from ZUMA-12.

## Results

### Patients

**Extended Data Fig. 1:**
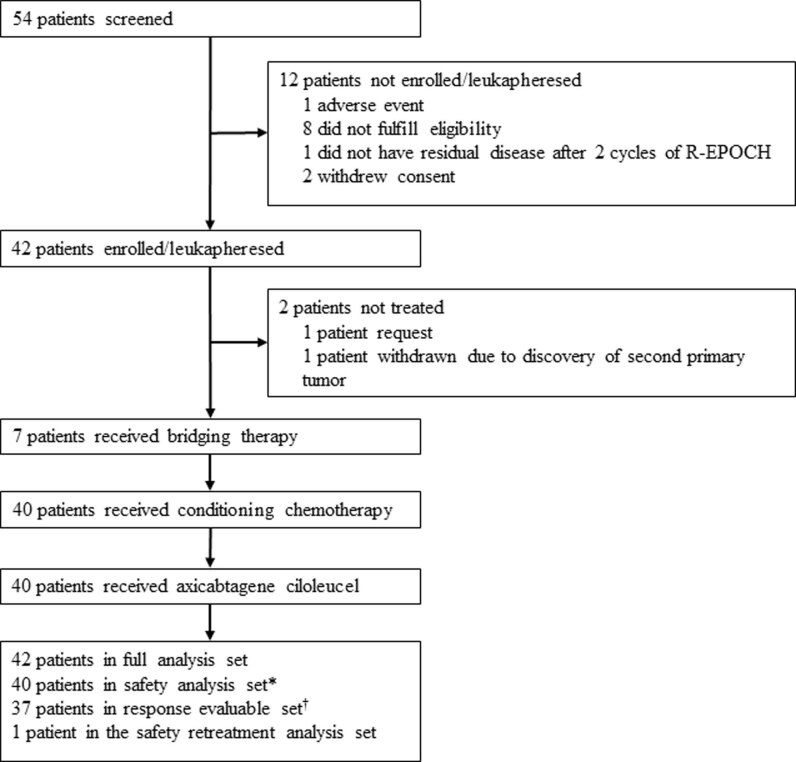
Patient flow diagram. *Includes all treated patients who received any dose of axicabtagene ciloleucel. †Includes all treated patients with centrally confirmed disease type (double- or triple-hit lymphomas) or IPI score ≥3 who received ≥1×10^6^ CAR T cells/kg. CAR, chimeric antigen receptor; IPI, International Prognostic Index.

Between 6 February 2019 and 22 October 2020, a total of 42 patients were enrolled and underwent leukapheresis (Extended Data Fig. 1 and Extended Data Table 1). Axi-cel was manufactured for all 42 patients and administered to 40. One patient did not receive treatment at their request, and one patient was withdrawn from the study before treatment due to the discovery of a second primary malignancy (Extended Data Fig. 1). The median time from leukapheresis to delivery of axi-cel product to the treatment facility was 18 days (range, 14–32; Extended Data Table 2). The median weight-adjusted dose of axi-cel received by patients weighing <100 kg (*n* = 31) was 2 × 10^6^ cells kg^–1^ (range, 2–2); all nine patients weighing ≥100 kg received the per-protocol maximum flat dose of 2 × 10^8^ cells. The date of data cutoff for the primary analysis was 17 May 2021, after which all treated patients had the opportunity to be followed for 6 months after the first disease assessment post-infusion. The median follow-up time among patients included in the primary efficacy analysis (*n* = 37) was 15.9 months (range, 6.0–26.7), and that among all patients treated with axi-cel (*n* = 40) was 17.4 months (range, 6.0–26.7).

All 40 treated patients met the eligibility criteria for having high-risk disease, defined by either double- or triple-hit lymphomas per investigator site assessment or LBCL with IPI ≥3. Additionally, all patients met the inclusion criteria of PET2^+^ by local review according to the Lugano classification^26^ (Deauville score of 4 or 5) after two cycles of chemoimmunotherapy with an anti-CD20 monoclonal antibody and an anthracycline-containing regimen, with a Deauville PET score of 4 (48%) or 5 (53%; Table 1). The median age was 61 years (range, 23–86; Table 1). Patients included 22 (55%) with diffuse LBCL (DLBCL), 16 (40%) with double- or triple-hit lymphomas and two (5%) with HGBL not otherwise specified (HGBL-NOS; Table 1). Most patients (95%) had stage III or IV disease and 78% had IPI ≥3 (Table 1). All treated patients received two cycles of one previous systemic therapy, most commonly R-CHOP (48%) or DA-EPOCH-R (45%). The median time from the last dose of previous therapy to leukapheresis was 1 month. Seven patients received nonchemotherapy bridging therapy after leukapheresis and before conditioning chemotherapy (Extended Data Fig. 1). Five patients received CNS prophylaxis.

**Table 1:** Baseline characteristics for all treated patients

Baseline characteristic	Patients (*n* = 40)
Age, median (range), years	61 (23–86)
≥65 years, *n* (%)	15 (38)
Male sex, *n* (%)	27 (68)
Histological disease type per investigator, *n* (%)	
DLBCL not otherwise specified	22 (55)
HGBL-NOS	2 (5)
Double- or triple-hit lymphoma	16 (40)
ECOG performance status score of 1^a^, *n* (%)	25 (63)
Disease stage, *n* (%)	
I or II	2 (5)
III or IV	38 (95)
IPI total score^b^, *n* (%)	
1 or 2	9 (23)
3 or 4	31 (78)
Deauville five-point scale, *n* (%)	
4	19 (48)
5	21 (53)
Bone marrow assessment at enrollment^c^, *n* (%)	
Lymphoma present	10 (25)
Double- or triple-hit status by FISH per central laboratory and IPI total score, *n* (%)^d^	
Double- or triple-hit and IPI ≥3	4 (10)
Double- or triple-hit only	6 (15)
IPI ≥3 only	20 (50)
Neither double- or triple-hit nor IPI ≥3	2 (5)
Double- or triple-hit not done and IPI ≥3	7 (18)
Double- or triple-hit not done and non-IPI ≥3	1 (3)
Double expression per central laboratory, *n* (%)	13 (33)
c-Myc expression per central laboratory, *n* (%)	21 (53)
Alterations by FISH, per investigator, *n* (%)	
*MYC*	19 (48)
*BCL2*	15 (38)
*BCL6*	10 (25)
Previous systemic therapy regimen (two cycles)^e^, *n* (%)	
R-CHOP	19 (48)
DA-EPOCH-R	18 (45)
Neither R-CHOP nor DA-EPOCH-R	6 (15)
Best response to two cycles of previous systemic therapy, *n* (%)	
PR	21 (53)
SD	2 (5)
PD	16 (40)
NE	1 (3)
Previous radiotherapy, *n* (%)	2 (5)
Received bridging therapy, *n* (%)	7 (17.5)

^a^Four patients had ECOG ≥2 at the time of diagnosis, which was changed to ECOG ≤1 before enrollment.

^b^IPI measured at initial diagnosis or any time between initial diagnosis and enrollment.

^c^Bone marrow assessment at baseline is the last assessment based on biopsy or PET–CT on or before first dose of conditioning chemotherapy.

^d^Of 6 patients reported to be double- or triple-hit per investigator, 3 remained inconclusive, 1 was determined not to be double- or triple-hit, and 2 were not tested by the central laboratory.

^e^Three patients received both R-CHOP and DA-EPOCH-R. Of the six patients who did not receive R-CHOP or DA-EPOCH-R, two received EPOCH-R, one received EPOCH (patient’s tumor was CD20^–^), one received EPOCH-R and intrathecal chemotherapy, one received R-mini-CHOP and one received R-CODOX-M. NE, not evaluable; R-CODOX-M, cyclophosphamide, vincristine, doxorubicin and high-dose methotrexate; R-mini-CHOP, rituximab and reduced-dose cyclophosphamide, doxorubicin, vincristine and prednisone

### Efficacy

**Fig. 1:**
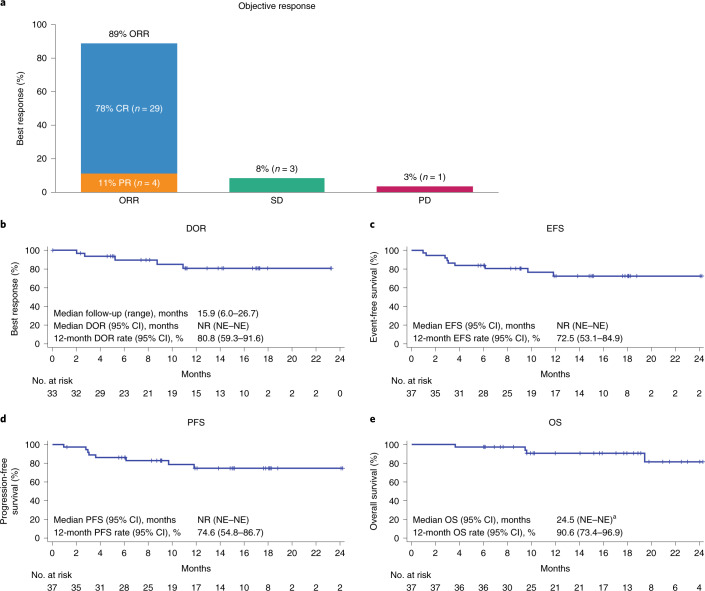
Best response, DOR, PFS, EFS and OS in patients included in primary efficacy analysis. **a**, Summary of best responses in efficacy-evaluable patients (*n* = 37). **b**–**e**, Kaplan–Meier plots for DOR (**b**), PFS (**c**), EFS (**d**) and OS (**e**) in efficacy-evaluable patients. ^a^One patient died following progression after month 24 (cause of death was progression). NR, not reached; NE, not evaluable.

According to protocol, the primary efficacy analysis was performed after all treated patients were followed for ≥6 months after the first post-infusion disease assessment, and included patients with centrally confirmed disease type (double- or triple-hit lymphomas) or IPI score ≥3 who received a target dose of 2 × 10^6^ CAR T cells kg^–1^. Among the 37 patients included in primary efficacy analysis, the primary endpoint of CRR was 78% (95% CI, 62–90; Fig. 1a). The median time to first complete response (CR) was 30 days (range, 27–207). The secondary endpoint of ORR was 89% (95% CI, 75–97) and the median time to first objective response was 29 days (range, 27–207). As of the data cutoff date, 25 patients (86% of complete responders and 68% of patients in primary efficacy analysis) had an ongoing CR while 27 patients (82% of objective responders and 73% of patients in the primary efficacy analysis) had an ongoing objective response.

**Fig. 2:**
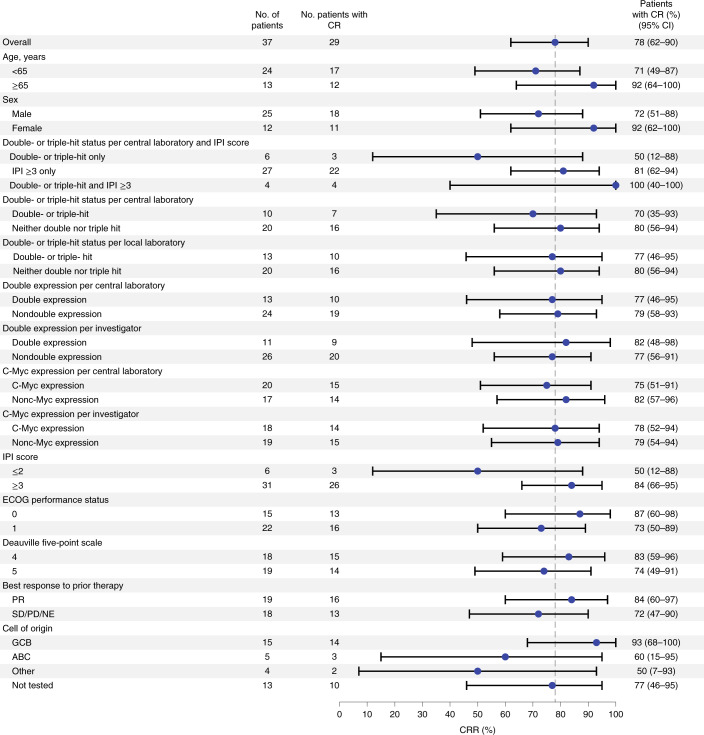
Forest plot representing subgroup analyses of CRR in patients included in primary efficacy analysis. Response assessments, according to Lugano classification^30^, are displayed regarding CRR for subgroups of interest within the efficacy-evaluable population (*n* = 37). Data for each group are presented as the proportion of patients with CR and 95% CI (based on the Clopper–Pearson method). Dashed vertical line represents the point estimate of the overall population.

**Extended Data Fig. 2:**
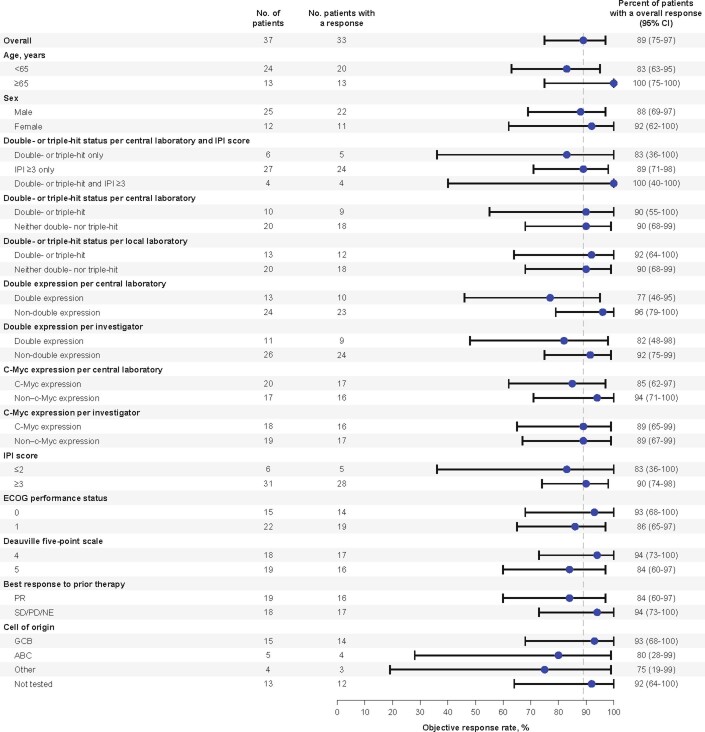
Subgroup analyses of objective response rates (N = 37). Response assessments were per Lugano Classification^26^. Data for each group are presented as the proportion of patients with an objective response, with a 95% CI (Clopper-Pearson method). The dotted vertical line presents the point estimate of overall population. ABC, activated B-cell; CI, confidence interval; ECOG, Eastern Cooperative Oncology Group; GCB, germinal center B-cell; IPI, International Prognostic Index; NE not evaluable; PD, progressive disease; PR, partial response; SD, stable disease.

Complete response rates and ORRs among key subgroups generally aligned with the overall patient population (Fig. 2 and Extended Data Fig. 2). All four patients with double- or triple-hit lymphomas and IPI score ≥3 achieved CR (Fig. 2), and all 13 patients aged ≥65 years achieved objective response (Extended Data Fig. 2). CRR for the six patients with double- or triple-hit lymphoma and IPI score ≤2 was lower than that of the overall population (50 versus 78%), although sample size was small (Fig. 2).

With a median follow-up of 15.9 months at the time of data cutoff, the medians for secondary endpoints of DOR, PFS and EFS had not yet been reached (Fig. 1b–d). The estimated rates for DOR, PFS and EFS at 12 months were 81% (95% CI, 59–92), 75% (95% CI, 55–87) and 73% (95% CI, 53–85), respectively (Fig. 1b–d). The 12-month estimated OS rate (secondary endpoint) was 91% (95% CI, 73–97; Fig. 1e). Of the 37 patients included in the primary efficacy analysis, 32 (86%) were still alive at the time of data cutoff. Efficacy outcomes were similar among all patients treated with axi-cel (*n* = 40; Extended Data Table 3).

Five patients experienced disease progression after an initial response to axi-cel at the time of data cutoff: one patient was retreated with axi-cel and achieved partial response (PR); two patients received subsequent therapies and did not respond; one patient was screened for axi-cel retreatment and is awaiting treatment; and one patient is still alive as of the data cutoff date, with subsequent therapies unknown. No patients experienced CNS relapse, a secondary endpoint. One patient achieved PR as best response to axi-cel and then proceeded to subsequent therapy, which included autologous stem cell transplantation, after which the patient achieved CR. Three patients achieved a best response of stable disease (SD) to axi-cel. At the time of data cutoff, one patient had not received subsequent therapy but was still alive while two patients had received subsequent therapy but died of progressive disease (PD). The one patient who had PD as their best response to axi-cel went on to receive subsequent therapies but died of PD.

### Safety

We assessed the secondary endpoint of the incidence of adverse events among all 40 treated patients. All 40 patients experienced at least one adverse event of any grade, with grade ≥3 adverse events experienced by 34 patients (85%). The most common treatment-emergent adverse events of any grade were pyrexia (100%), headache (70%) and decreased neutrophil count (55%; Table 2). The most common treatment-emergent adverse events of grade ≥3 were decreased neutrophil count (53%), reduced white blood cell count (43%) and anemia (30%; Table 2).

**Table 2:** Adverse events occurring in ≥15% of all treated patients, by worst grade

Adverse event^a^, *n* (%)	Grade 1	Grade 2	Grade ≥3^c^	Total
Any adverse event^b^	1 (3)	5 (13)	34 (85)	40 (100)
Pyrexia	8 (20)	28 (70)	4 (10)	40 (100)
Headache	19 (48)	9 (23)	0 (0)	28 (70)
Neutrophil count decreased	0 (0)	1 (3)	21 (53)	22 (55)
Nausea	9 (23)	11 (28)	1 (3)	21 (53)
Diarrhea	14 (35)	6 (15)	0 (0)	20 (50)
Fatigue	8 (20)	12 (30)	0 (0)	20 (50)
White blood cell count decreased	0 (0)	1 (3)	17 (43)	18 (45)
Hypotension	8 (20)	5 (13)	1 (3)	14 (35)
Anemia	0 (0)	1 (3)	12 (30)	13 (33)
Chills	10 (25)	1 (3)	0 (0)	11 (28)
Confusional state	7 (18)	2 (5)	2 (5)	11 (28)
Hypokalemia	8 (20)	2 (5)	1 (3)	11 (28)
Hypoxia	3 (8)	3 (8)	5 (13)	11 (28)
Encephalopathy	2 (5)	2 (5)	6 (15)	10 (25)
Sinus tachycardia	9 (23)	1 (3)	0 (0)	10 (25)
Tremor	8 (20)	2 (5)	0 (0)	10 (25)
Constipation	6 (15)	2 (5)	0 (0)	8 (20)
Decreased appetite	3 (8)	5 (13)	0 (0)	8 (20)
Platelet count decreased	1 (3)	1 (3)	6 (15)	8 (20)
Vomiting	3 (8)	5 (13)	0 (0)	8 (20)
Alanine aminotransferase increased	1 (3)	3 (8)	3 (8)	7 (18)
Hypophosphatemia	0 (0)	5 (13)	2 (5)	7 (18)
Muscular weakness	4 (10)	2 (5)	1 (3)	7 (18)
Insomnia	5 (13)	1 (3)	0 (0)	6 (15)
Neutropenia	0 (0)	1 (3)	5 (13)	6 (15)

^a^Adverse events include those with onset on or after axi-cel infusion date, coded using MedDRA v.23.1 and graded according to CTCAE v.5.0.

^b^The first row, showing any adverse event, displays the worst grade event experienced by each of the 40 treated patients.

^c^One grade 5 event occurred and was reported as COVID-19.

Cytokine release syndrome of any grade occurred in all 40 patients (Table 3). Most cases of CRS were grade 1 or 2 (93%), with three (8%) being grade ≥3, and no patients died from CRS. The most common CRS symptoms of any grade were pyrexia (100%), hypotension (30%), chills (25%) and hypoxia (23%; Table 3). The median time to onset for CRS after infusion with axi-cel was 4 days (range, 1–10; Table 3). All 40 patients (100%) had their CRS resolved by data cutoff, with a median event duration of 6 days. CRS was managed with tocilizumab in 25 patients (63%), steroids in 14 patients (35%) and vasopressors in one (3%).

**Table 3:** Adverse events of interest occurring in ≥15% of all treated patients, by worst grade

Adverse event^a^, *n* (%)	Grade 1	Grade 2	Grade ≥3	Total
Subjects with any CRS^a^	27 (68)	10 (25)	3 (8)	40 (100)
Pyrexia	8 (20)	28 (70)	4 (10)	40 (100)
Hypotension	7 (18)	5 (13)	0 (0)	12 (30)
Chills	9 (23)	1 (3)	0 (0)	10 (25)
Hypoxia	2 (5)	2 (5)	5 (13)	9 (23)
Sinus tachycardia	6 (15)	0 (0)	0 (0)	6 (15)
Subjects with any neurologic events	14 (35)	6 (15)	9 (23)	29 (73)
Confusional state	7 (18)	2 (5)	2 (5)	11 (28)
Encephalopathy	2 (5)	2 (5)	6 (15)	10 (25)
Tremor	8 (20)	2 (5)	0 (0)	10 (25)

^a^Adverse events include those with onset on or after axi-cel infusion date and coded using MedDRA v.23.1. Neurologic events were identified using the modified blinatumomab registrational study^35^. CRS was graded according to Lee et al.^36^. The severity of all adverse events, including neurologic events and symptoms of CRS, was graded according to CTCAE v.5.0.

Neurologic events of any grade were experienced by 29 patients (73%), with seven events (18%) being grade 3 and two (5%) being grade 4. No patient died from a neurologic event. The most common neurologic events of any grade were confusional state (28%), encephalopathy (25%) and tremor (25%). Grade 4 serious adverse events of encephalopathy were experienced by two patients (5%), and both events had fully resolved by data cutoff. The median time to onset for neurologic events was 9 days (range, 2–44) and median event duration was 7 days. As of data cutoff date, neurologic events had resolved in 28 patients, with one patient experiencing an ongoing neurologic event of grade 1 tremor. Neurologic events were managed with steroids in 13 patients (33%) and tocilizumab in one (3%). Additionally, no patient required mechanical ventilation for the management of neurologic events.

Serious adverse events of any grade were experienced by 18 patients (45%; Extended Data Table 4). A total of 13 patients (33%) experienced infection of any grade (Extended Data Table 5); three of these events were SARS-CoV-2 infection, including one each of grade 2 and grade 5 infection (these patients did not report receiving a COVID-19 vaccine) and one of grade 3 COVID-19 pneumonia (the patient was fully vaccinated against COVID-19). The remaining ten adverse events of infection were grade 3 (*n* = 4), grade 2 (*n* = 3) or grade 1 (*n* = 3) and included a grade 1 event of cytomegalovirus infection reactivation. A total of four patients (10%) had adverse events of hypogammaglobulinemia, all of which were grade 2. Grade ≥3 cytopenias were present in 68% of patients (*n* = 27); grade ≥3 cytopenias present on or after day 30 were experienced by eight patients (20%). All cytopenias of any grade had resolved by the data cutoff date, with a median duration of 0.5 months. No cases of tumor lysis syndrome, replication-competent retrovirus or secondary malignancies related to axi-cel were reported.

A total of six patients (15%) treated with axi-cel died, four from PD after proceeding to subsequent therapies (10%). The other two deaths were due to COVID-19 (day 350 post-infusion) and septic shock (day 287 post-infusion). Only the one death from COVID-19 was reported as an adverse event. The instance of septic shock was reported after the patient had proceeded to subsequent therapy.

### Biomarkers

**Fig. 3:**
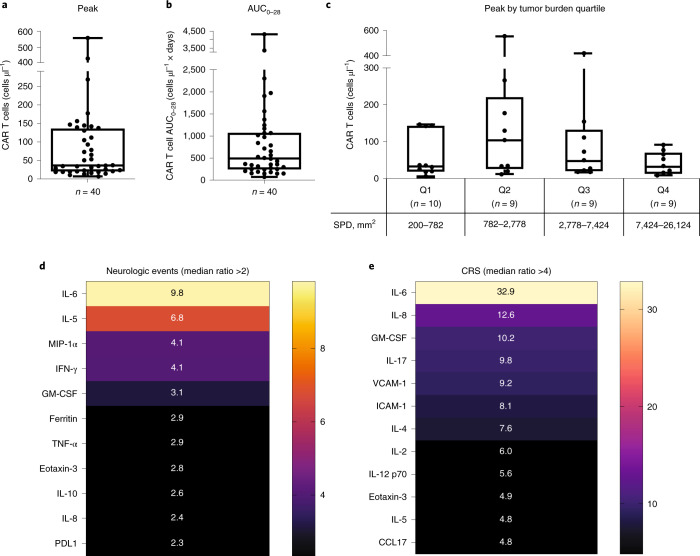
Levels of CAR T cells in blood and association of serum cytokines with adverse events. **a**,**b**, Peak levels of CAR T cells reached in the blood (**a**) and AUC_0–28_ (**b**). **c**, Peak CAR T-cell levels by tumor burden quartile, as determined by SPD of target lesions. For all box plots, the center line denotes the median, box limits the upper and lower quartiles and whiskers 1.5× interquartile range. **d**,**e**, Selected cytokines associated with neurologic events (**d**) and CRS (**e**) with median ratio >2 based on ratio of median peak levels in grade ≥3 events to grade 2, grade 1 or no events. Resultant values with ratio >2 for neurologic events and >4 for CRS are depicted. Color scales indicate the median ratio, with increasing intensity representing higher values. CCL, chemokine ligand; ICAM-1, intercellular adhesion molecule-1; MIP-1, macrophage inflammatory protein-1; VCAM-1, vascular cell adhesion protein-1.

CAR T-cell expansion was observed in peripheral blood in all 40 patients. The median peak CAR T-cell level, a secondary endpoint, was 36.27 cells μl^–1^ and median area under the curve (AUC) in a plot of CAR T cells in blood between days 0 and 28 (AUC_0–28_) was 495.38 cells μl^–1^ × days (Fig. 3a,b). Median time to peak anti-CD19 CAR T-cell levels in blood was 8 days (range, 8–37; Extended Data Table 6). Pharmacokinetic profiles were similar across patients in different diagnostic categories, including those with double- or triple-hit lymphoma and IPI score ≥3 (Extended Data Table 6). At 6 months after infusion, 13 of 21 patients (62%) with evaluable samples maintained low, but detectable, levels of CAR gene-marked cells in blood. Three patients had samples evaluable at the approximate time of their relapse, two of whom had detectable CAR gene-marked cells in blood. Two additional patients who relapsed did not have evaluable samples at the time of relapse, although they had detectable CAR gene-marked cells in blood at the final time point assessed before relapse (days 85 and 145).

The median peak levels of CAR T cells and AUC_0–28_ among patients who relapsed or did not respond trended higher but were not statistically significantly different to those who had an ongoing response as of the data cutoff date. CAR T-cell persistence declined similarly among patients who had an ongoing response compared with those who had relapsed disease or did not respond to axi-cel. Additionally, no trend was found between peak or AUC_0–28_ and response.

Patients with a tumor burden per sum of product diameters (SPD) in the second and third quartiles of baseline tumor burden value appeared to have higher median peak levels of CAR T cells and lower average time to peak than those with baseline tumor burden in the first and fourth quartiles (although low numbers of patients in each quartile limited comparison and differences were not statistically significant; Fig. 3c). Patients who experienced grade ≥3 CRS (*n* = 3) had peak levels of CAR T cells in blood and AUC_0–28_ with a median ratio of fourfold more (144.2 cells μl^–1^ (range 10.4–268.4) versus 35.7 cells μl^–1^ (range, 6.8–560.3)), and 2.2-fold more (1,067.7 cells μl^–1^ × days (range, 151.4–2303.3) versus 486.7 cells μl^–1^ × days (range, 74.5–4,288.0) than that of patients who experienced grade 2, grade 1 or no CRS. Patients who experienced grade ≥3 neurologic events (*n* = 9) had peak levels of CAR T cells in blood and AUC_0–28_ with a median ratio 2.1- and 2.3-fold more, respectively, than that of patients who experienced grade 2, grade 1 or no neurologic event, although the difference between the two groups was not statistically significant.

Serum levels of cytokines were also assessed as a secondary endpoint, with median time to peak of most serum cytokines being within 8 days. Several serum analytes were elevated in patients experiencing grade ≥3 CRS or neurologic events compared with those who had grade 2, grade 1 or no CRS or neurologic events (Fig. 3d,e). Among serum analytes that were at least twice as high at peak among patients who experienced grade ≥3 neurologic events compared with those who did not (Fig. 3d), interleukin (IL)-5, macrophage inflammatory protein-1α (MIP-1α), interferon (IFN)-γ, granulocyte-macrophage colony-stimulating factor (GM-CSF), ferritin, tumor necrosis factor (TNF)-α, IL-10, IL-8 and programmed death-ligand 1 (PDL1) were all determined to be significantly higher (*P* < 0.05). Serum cytokine peak values that were at least fourfold higher among patients who experienced grade ≥3 CRS compared with those who did not were analyzed, but were not assessed for statistical significance due to the small patient population size who experienced grade ≥3 CRS (*n* = 3). The most highly elevated serum cytokines among those experiencing grade ≥3 CRS were IL-6, IL-8 and GM-CSF (Fig. 3e). Axi-cel product characteristics are shown in Extended Table 2. The median proportion of naïve-like T cells (CCR7^+^ CD45RA^+^ ) among total T cells infused was 35% (range, 7–80).

## Discussion

Patients with high-risk LBCL, including those diagnosed with double- or triple-hit lymphomas and LBCL with high IPI scores, have a poor prognosis with currently available therapy options, demonstrating a high need for effective therapies earlier in their disease course^2,12,27^. Moreover, prospective trials of therapies primarily in these high-risk patient populations are very limited^13^. Clinical trials of frontline therapy in high-risk LBCL are rare and are challenging to conduct due to the risks of disease progression during screening^13^. To our knowledge, ZUMA-12 is the first prospective phase 2 trial to evaluate CAR T-cell therapy as part of first-line treatment in patients with high-risk LBCL. Importantly, the high-risk patient population for this trial was selected based on both high-risk features identified at the time of initial diagnosis (IPI and double- or triple-hit status) and dynamic risk assessment utilizing interim PET after an incomplete regimen of two cycles of standard first-line chemoimmunotherapy. Forty-five per cent of patients in ZUMA-12 had either SD (5%) or PD (40%) at study entry, suggesting that a substantial proportion had primary chemorefractory disease, a group historically associated with median OS of approximately 6 months and long-term survival estimates near 10% (refs. ^28,29^).

In this high-risk LBCL population, axi-cel demonstrated a high rate of rapid and CRs at 78%, and a median time to first CR of 30 days; among all patients treated with axi-cel, 80% achieved CR. Results from ZUMA-12 compare favorably with those of the GELA study of rituximab, doxorubicin, cyclophosphamide, vindesine, bleomycin and prednisone (R-ACVBP) or R-CHOP-14 induction in young patients with high-risk DLBCL, wherein the primary outcome of achieving CRR >50% after four cycles of induction regimen was not met in either randomization group^11^. CRRs in ZUMA-12 were numerically higher than those with DA-EPOCH-R (59%) and R-CHOP (60%) in the Alliance/CALGB 50303 trial, and patients with high-risk disease were underrepresented in that trial^14^. Additional measures of efficacy also indicated positive outcomes for patients with high-risk LBCL in ZUMA-12, with an ORR of 89% and an ongoing response rate of 73% after a median of 15.9 months of follow-up as of the data cutoff date.

Recent randomized controlled trials of second-line CAR T-cell therapy in LBCL also suggest that treatment with CAR T-cell therapy in earlier lines is a viable option regardless of high-risk disease characteristics, with similar EFS among patients with high-grade B-cell lymphoma or high IPI and the overall population in each study^30,31^. ORRs among patients with high-grade B-cell lymphoma (with or without double- or triple-hit) treated with axi-cel in ZUMA-7 were similar to those in ZUMA-12 (84 versus 89%)^30^. Of note, a separate randomized control trial in a similar patient population (BELINDA) showed differing results, possibly due to differences in trial design, CAR T-cell product, timing of infusion and patient population, among other factors^32,33^. The CRR in ZUMA-12 appeared higher than that in patients with double- or triple-hit lymphomas and refractory disease in the ZUMA-1 study of axi-cel (78 versus 67%), supporting the use of axi-cel in earlier lines, although comparisons are limited by the sample size in ZUMA-1 (*n* = 6)^16^. CRs were generally consistent among key high-risk features of double- or triple-hit status and IPI score ≥3, and patients with IPI score ≥3 achieved 84% CRR. Importantly, medians of DOR, PFS and EFS were not reached after >15 months of median follow-up, suggesting that responses to axi-cel have the potential to be durable.

Safety with axi-cel was manageable in these high-risk patients, and no new safety signals were reported with axi-cel in an earlier line. The incidence of any treatment-emergent adverse events of grade ≥3 was 85%, which appears numerically lower than the incidence of 95% reported in the ZUMA-1 study^23^. The incidence of grade ≥3 CRS in the ZUMA-12 study (8%) also appeared slightly numerically lower than that observed in ZUMA-1 (13%). Similarly, the incidences of grade ≥3 neurologic events in the ZUMA-12 study (23%) were slightly lower than observed in ZUMA-1 (28%)^23^. Observed differences between these trials of axi-cel may be connected to disease-related factors and improvements in toxicity management over time, although our study was not designed to compare outcomes between trials. Both CRS and neurologic events were generally manageable and reversible, suggesting that safety management guidelines for axi-cel during the ZUMA-12 study kept higher-grade adverse events of special interest to a minimum.

The median tumor burden was lower in ZUMA-12 than previously reported in ZUMA-1 Cohort 1 (2,778 versus 3,684 mm^2^), and the median time to peak levels of CAR T cells in blood was 8 days for ZUMA-12 compared with 7 days for ZUMA-1 Cohort 1 (ref. ^23^). CAR T-cell expansion by peak and AUC appeared higher in ZUMA-12 than in ZUMA-1 (ref. ^16^). Pharmacokinetic profiles were similar in patients with double- or triple-hit lymphoma and in LBCL with IPI score ≥3. In ZUMA-1, responders had higher peak levels of CAR T cells in blood and higher AUC_0–28_ than nonresponders^23^. This trend was not observed among patients in ZUMA-12, which could be due to differences in either axi-cel product characteristics or patient responses to axi-cel when administered as part of first-line therapy. However, only four patients treated in ZUMA-12 did not respond to therapy, so any trends observed may not be representative of a larger population of nonresponders. The median peak serum analyte levels associated with grade ≥3 neurologic events in ZUMA-12 were consistent with previous findings in ZUMA-1, with several cytokines, including IL-10, IL-1Ra, IFN-γ and IL-8, being statistically significantly elevated at peak values among patients in both ZUMA-1 and ZUMA-12 who experienced grade ≥3 neurologic events^23^. In ZUMA-12, median peak levels of cytokines that were substantially higher with both grade ≥3 CRS and neurologic events included IL-6, IL-5, IL-8, GM-CSF and eotaxin-3.

As with all personalized therapies, including CAR T-cell therapies such as axi-cel, reliable and expedient manufacturing is paramount. Successful manufacture of the therapy is especially important for patients with aggressive and rapidly progressing disease and in those recently treated with chemotherapy. In ZUMA-12, axi-cel was successfully manufactured in 100% of the 42 enrolled patients, with axi-cel delivered to the site a median of 18 days after leukapheresis. This high rate of manufacturing success and quick delivery time demonstrated that manufacturing issues or delays were not a factor in the treatment of patients in ZUMA-12, and recent exposure to R-CHOP or DA-EPOCH-R did not affect generation of axi-cel. Furthermore, a higher proportion of naïve-like T cells (CCR7^+^ CD45RA^+^) in axi-cel products in ZUMA-12 versus ZUMA-1, a cell phenotype previously associated with greater expansion of CAR T cells, supports the notion that T-cell functionality—and thereby clinical efficacy—may be improved if CAR T-cell therapy is moved to earlier lines of therapy^25^.

This study had certain limitations. Dynamic risk assessment was conducted using PET2^+^ by Deauville score, which may be less reproducible than other prognostic assessments such as ΔSUV, although the prognostic value of these two measures has not been studied in high-risk LBCL and some patients in ZUMA-12 did not have a baseline PET^34^. Assessments of quality of life were not conducted in this study. Further, immunoglobulin levels, CD19 antigen detection at relapse and further genomic analyses at baseline beyond the presence of *MYC*, *BCL2* and *BCL6* alterations were not assessed. The primary analysis of ZUMA-12 was designed to assess CR to axi-cel, and durability of responses will be better assessed with longer follow-up.

In conclusion, our findings from the primary analysis of ZUMA-12 provide evidence that axi-cel is safe and highly effective as part of first-line treatment for adult patients with high-risk LBCL, including those with positive interim PET2, together with either double- or triple-hit lymphomas or with IPI score ≥3. Further investigation is warranted in these patient populations to determine the benefit of axi-cel as first-line therapy compared with standard chemoimmunotherapy.

## Methods

### Study design and participants

This phase 2, single-arm, open-label, registrational ZUMA-12 trial (Clinicaltrials.gov no. NCT03761056) was conducted at seven medical centers in the United States, Australia and France (Extended Data Table 1). The study protocol and statistical analysis plan are included in the Supplementary Information. Patients were considered eligible for the study if they were male or female, 18 years or older and had LBCL with one or more of the following features: double-hit or triple-hit lymphoma (that is, high-grade B-cell lymphoma with *MYC* and *BCL2* and/or *BCL6* translocations), as determined by the investigator using fluorescent in situ hybridization (FISH); or other histologically confirmed LBCL, as defined by the World Health Organization (WHO) in 2016 (ref. ^10^) with an IPI score of ≥3 at initial diagnosis or any time between initial diagnosis and enrollment. Lymphoma types included under eligibility criteria were: (1) DLBCL not otherwise specified, including types germinal center B-cell (GCB) and ABC; (2) intravascular LBCL; (3) T-cell/histiocyte-rich LBCL; (4) DLBCL associated with chronic inflammation; (5) Epstein–Barr virus^+^ DLBCL not otherwise specified; and (6) HGBL-NOS. Additional eligibility criteria required that patients were PET^+^ according to the Lugano classification^26^ (Deauville score of 4 or 5) after two cycles of chemoimmunotherapy consisting of an anti-CD20 monoclonal antibody (unless the investigator determined that the tumor was CD20^–^), and an anthracycline-containing regimen. At least 2 weeks must have elapsed since any previous systemic therapy at the time the patient was scheduled for leukapheresis. Additional inclusion criteria were as follows: (1) no evidence, suspicion and/or history of CNS involvement; (2) Eastern Cooperative Oncology Group (ECOG) performance status of 0 or 1; and (3) toxicities due to previous therapy stabilized and recovered to grade 1 or lower, with the exception of clinically nonsubstantial toxicities such as alopecia. Patients must also have adequate bone marrow, renal, hepatic, pulmonary and cardiac function (absolute neutrophil count ≥1,000 μl^–1^; platelet count ≥75,000 μl^–1^; absolute lymphocyte count ≥100 μl^–1^; creatinine clearance ≥60 ml min^–1^; serum alanine aminotransferase and aspartate aminotransferase ≤2.5 upper limit of normal; total bilirubin ≤1.5 mg dl^–1^, except in patients with Gilbert’s syndrome; cardiac ejection fraction ≥50%); no evidence of pericardial effusion (except trace or physiological) as determined by echocardiogram, and no clinically notable electrocardiogram findings; no clinically notable pleural effusion; and baseline oxygen saturation >92% on room air. Females of childbearing potential must have had a negative serum or urine pregnancy test.

Patients were excluded from the trial if any of the following applied: a history of malignancy other than nonmelanoma skin cancer or carcinoma in situ, unless disease-free for at least 3 years; a history of Richter’s transformation of chronic lymphocytic leukemia or PMBCL; a history of autologous or allogeneic stem cell transplantation; previous CD19-targeted therapy previous CAR T-cell therapy or other genetically modified T-cell therapy; history of severe, immediate hypersensitivity reaction attributed to aminoglycosides; presence or suspicion of fungal, bacterial, viral or other infection that is uncontrolled or requiring intravenous antimicrobials for management (simple urinary tract infection and uncomplicated bacterial pharyngitis are permitted if responding to active treatment and after consultation with the sponsor’s medical monitor); history of human immunodeficiency virus infection or acute or chronic active hepatitis B or C infection (patients with previous hepatitis infection must have cleared their infection as determined by standard serological and genetic testing according to current Infectious Diseases Society of America guidelines or applicable country guidelines); presence of any indwelling line or drain (dedicated central venous access catheters were permitted); detectable cerebrospinal fluid malignant cells, brain metastases or active CNS lymphoma; history or presence of CNS disorder or any autoimmune disease with CNS involvement; cardiac atrial or cardiac ventricular lymphoma involvement; history of clinically substantial cardiac disease within 12 months of enrollment; required urgent therapy due to tumor mass effects; a primary immunodeficiency or history of autoimmune disease resulting in end organ injury or requiring systemic immunosuppression/systemic disease-modifying agents within the past 2 years; history of symptomatic deep vein thrombosis or pulmonary embolism within 6 months of enrollment; any medical condition likely to interfere with assessment of safety or efficacy of study treatment; history of severe immediate hypersensitivity reaction to any of the agents used in this study; or live vaccine ≤6 weeks before the planned start of a conditioning regimen. Women of childbearing potential who were pregnant or breastfeeding, and patients of either gender who were not willing to practice birth control from the time of consent through to 6 months after the completion of conditioning chemotherapy or axi-cel infusion, whichever is longer, were excluded. In the investigator’s judgment, if the patient was unlikely to complete all protocol-required study visits or procedures, including follow-up visits, or comply with the study requirements for participation, they were excluded.

All patients enrolled in the study provided written informed consent, and the study was conducted in accordance with applicable International Conference on Harmonisation Good Clinical Practice Guidelines, the principles of the Declaration of Helsinki and any applicable local laws and regulations. The protocol was approved by the institutional review board or independent ethics committee at each study site (City of Hope National Medical Center, Moffitt Cancer Center, MD Anderson Cancer Center, Banner MD Anderson Cancer Center, Vanderbilt–Ingram Cancer Center, Peter MacCallum Cancer Center and Hopital Saint-Louis)–Service Hematologic Seniors) and was provided to the key sponsor contact. Patients were not compensated for trial participation beyond receipt of therapy and associated care. Patients may be compensated for study-related illness or injury pursuant to the information outlined in the injury section of the informed consent form.

### Procedures

Eligible patients underwent leukapheresis to obtain cells for use in manufacture of the axi-cel CAR T-cell product. Patients then received intravenous conditioning chemotherapy of cyclophosphamide (500 mg m^–2^) and fludarabine (30 mg m^–2^) on days −5, −4 and −3, followed by a single intravenous infusion of axi-cel at a target dose of 2 × 10^6^ anti-CD19 CAR T cells kg^–1^ on day 0. The minimum allowable dose of axi-cel was 1 × 10^6^ anti-CD19 CAR T cells kg^–1^, and patients weighing ≥100 kg were administered a maximum flat dose of axi-cel at 2 × 10^8^ anti-CD19 CAR T cells. Patients were monitored for at least 7 days at a healthcare facility after axi-cel infusion. At the discretion of the investigator, nonchemotherapy bridging therapy, including corticosteroids or high-dose methylprednisolone plus rituximab, was permitted for patients with high disease burden at screening or baseline assessments. Nonchemotherapy bridging therapy was to have been administered after leukapheresis/enrollment and completed as early as 7 days before the start of conditioning chemotherapy, depending on the bridging therapy type.

Within approximately 5 days of eligibility, laboratory monitoring began and then continued on days −5, −4 and −3; day 0; and days 1–7 after infusion. Post-treatment follow-up monitoring occurred at weeks 2 and 4, months 2 and 3 and then every 3 months thereafter until month 24. Disease response assessment was performed locally by PET–CT at week 4, month 3 and then every 3 months thereafter until month 24 or until disease progression, whichever occurred first.

All adverse events observed by the investigator or reported by the patient that occurred from enrollment up to 3 months after treatment with axi-cel infusion were reported. After 3 months, targeted adverse events (for example, infections; neurologic, hematological and autoimmune disorders; and secondary malignancies) were monitored and reported for 24 months after treatment with axi-cel or until disease progression, whichever occurred first. For patients who were enrolled but did not receive axi-cel, the adverse event-reporting period ended 30 days after the final study-specific procedure (for example, leukapheresis, conditioning chemotherapy). In review of adverse events, investigators assessed and reported whether the adverse event was potentially related to (1) axi-cel; (2) conditioning chemotherapy; (3) any protocol-required study procedure or treatment; (4) disease progression; (5) concurrent disease; (6) concomitant medication; or (7) other. Adverse events were coded using Medical Dictionary for Regulatory Activities (MedDRA) v.23.1 and graded according to the National Cancer Institute Common Terminology Criteria for Adverse Events (CTCAE) v.5.0. The overall status of CRS was graded according to a modification of the grading system proposed by Lee and colleagues^36^; individual CRS symptoms were graded according to CTCAE v.5.0. Neurologic events were also graded using CTCAE v.5.0, and were identified using a modified search strategy based on known neurologic events associated with anti-CD19 immunotherapy^35^. Guidelines for management of treatment-related toxicities were updated subsequent to increased experience with axi-cel. Patients could be removed from the study due to patient withdrawal of consent for further follow-up, investigator discretion, loss to follow-up or death.

An independent Data Safety Monitoring Board (DSMB) was chartered to meet and review serious adverse events and suspected unexpected serious adverse reactions on a semiannual basis through the primary analysis data cutoff. The DSMB met to review safety data after 15 patients were enrolled and treated with axi-cel and had an opportunity to be followed for 3 months after infusion. The DSMB also made trial conduct recommendations on an ongoing basis based on an analysis of risk versus benefit. All data were collected in an electronic case report form system between study initiation on 29 January 2019 and the data cutoff date for the primary analysis of 17 May 2021.

### Outcomes

The primary endpoint was CRR according to the Lugano classification^26^ as determined by the study investigators. Secondary endpoints were ORR (defined as the incidence of either CR or PR); DOR (among patients experiencing an objective response, defined as the time from the first objective response to events of disease progression or death from any cause); EFS (defined as the time from the axi-cel infusion date to the earliest date of the events of disease progression, commencement of subsequent new antilymphoma therapy, including stem cell transplant, or death from any cause); PFS (defined as the time from axi-cel infusion date to the date of the events of disease progression or death from any cause); OS (defined as the time from axi-cel infusion to the date of the event of death from any cause); incidence of adverse events; levels of anti-CD19 CAR T cells in blood; levels of cytokines in serum; and associations between biomarker levels and clinical outcomes.

### Biomarker analyses

The presence, expansion and persistence of anti-CD19 CAR T cells in peripheral blood were measured using TaqMan quantitative polymerase chain reaction (Thermo Fisher Scientific) and confirmed by droplet digital PCR (Bio-Rad Laboratories) according to the manufacturer’s instructions^25^. Serum cytokines were analyzed either by Simple Plex (Simpleprotein) according to the manufacturer’s instructions or Luminex (EMD Millipore) or V-Plex Multiplex assay panels (Meso Scale Discovery)^25^. T-cell phenotypes in axi-cel products were assessed for CCR7 and CD45RA expression by multicolor flow cytometry using established protocols and antibodies^37^. Briefly, the analysis employed a cell-gating strategy that selected viable CD3^+^ by exclusion of dead/apoptotic cells using 7-amino-actinomycin D. Data analysis was performed using FlowJo software v.10 (Supplemental Fig. 1).

### Statistical analysis

This trial used a single-arm design to estimate CRR in patients with high-risk LBCL who were treated with axi-cel. No formal hypothesis was tested. The CRR targeted in this study was 60% among treated patients, which included all patients who received infusion of axi-cel and determined to be clinically meaningful after assessment of a previous trial in similar high-risk populations with CRR <50% in both arms^11^. With a total sample size of 40 patients, an observed CRR of 60% will yield 80% CI for the response rate with maximum half-width ≤11%, corresponding to a lower limit of at least 48.6% and representing an improvement in response rate. Two-sided 95% CIs were calculated with the Clopper–Pearson method. The primary analysis was performed after all treated patients had an opportunity to be assessed for response 6 months after week 4 disease assessment. Patients with centrally confirmed disease type (double- or triple-hit lymphomas) or IPI score ≥3 and who received a target dose of 2 × 10^6^ CAR T cells kg^–1^ (minimally 1 × 10^6^ CAR T cells kg^–1^) were included in the primary efficacy analysis. Response was also measured among prespecified subgroups of disease and patient characteristics among efficacy-eligible patients. All patients who were treated with axi-cel were included in the safety and biomarker analyses.

Time-to-event analyses were assessed using the Kaplan–Meier method. For EFS, patients who were alive, in response and with no new antilymphoma therapy (including stem cell transplantation or axi-cel retreatment) were censored at their last evaluable disease assessment. For PFS and DOR, patients who had not died or had disease progression were censored at their last evaluable disease assessment before the data cutoff date or the start of subsequent therapy, including stem cell transplantation or axi-cel retreatment. For OS, patients who were alive were censored at the last date they were known to be alive or the data cutoff date, whichever occurred first. Patients who died after the data cutoff date were censored at the data cutoff date.

Pharmacokinetic and pharmacodynamic analyses were reported as descriptive summaries. No formal hypothesis testing was performed for pharmacokinetic and pharmacodynamic analyses. Wilcoxon rank-sum test was used to evaluate exploratory associations among biomarker levels and clinical outcomes; *P* values are descriptive and were not adjusted for multiplicity. Efficacy endpoints were evaluated in all patients treated with axi-cel at a dose of at least 1 × 10^6^ anti-CD19 CAR T cells kg^–1^ and with centrally confirmed disease type (double- or triple- hit lymphoma) or IPI score ≥3. Safety analyses included all treated patients. All statistical analyses were done using SAS v.9.4.

### Reporting Summary

Further information on research design is available in the Nature Research Reporting Summary linked to this article.

## Online content

Any methods, additional references, Nature Research reporting summaries, source data, extended data, supplementary information, acknowledgements, peer review information; details of author contributions and competing interests; and statements of data and code availability are available at 10.1038/s41591-022-01731-4.

### Supplementary information

Supplementary Information Supplementary Fig. 1, and redacted study protocol and SAP.

Reporting Summary

## Figures and Tables

**Extended Data Table 1:**
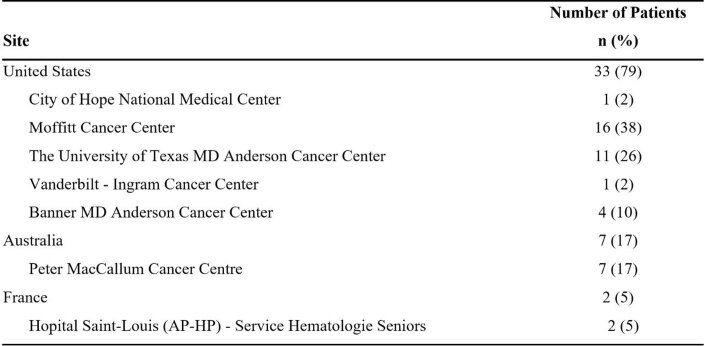
Patient enrollment by country and study site (*n* = 42)

**Extended Data Table 2:**
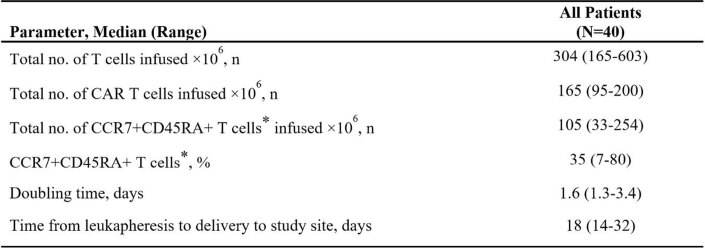
Axi-cel product characteristics in all treated patients (*n* = 40)

* Data are reported based on the total number of T cells infused and not the CAR^+^ T-cell population. Axi-cel, axicabtagene ciloleucel; CAR, chimeric antigen receptor; CCR7, C-C chemokine receptor type 7.

**Extended Data Table 3:**
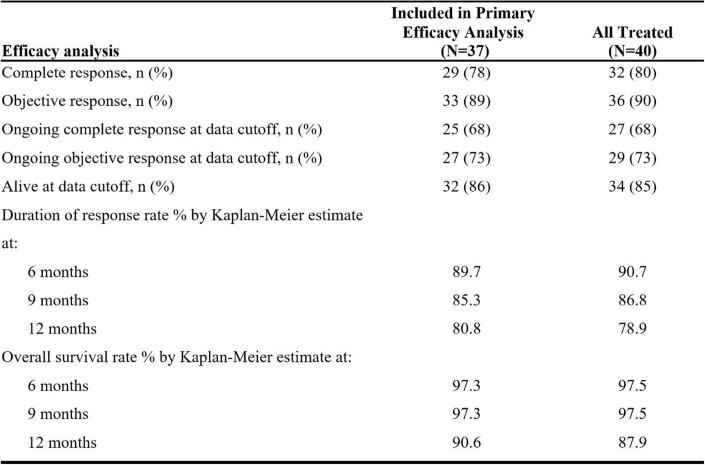
Key efficacy results for both patients included in the primary analysis and all treated patients

**Extended Data Table 4:**
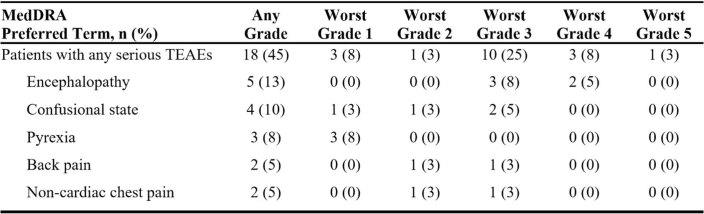
Serious adverse events occurring in at least two treated patients (*n* = 40)

TEAE includes all AEs with onset on or after axicabtagene ciloleucel infusion date. AEs with onset during retreatment period are excluded. Multiple incidences of the same AE in one patient are counted once at the worst grade for that patient. Preferred terms are sorted in descending order of frequency count in any grade. AEs are coded using MedDRA v.23.1 and graded according to CTCAE v.5.0. AE, adverse event; CTCAE, Common Terminology Criteria for Adverse Event; MedDRA, Medical Dictionary for Regulatory Activities; TEAE, treatment-emergent adverse event.

**Extended Data Table 5:**
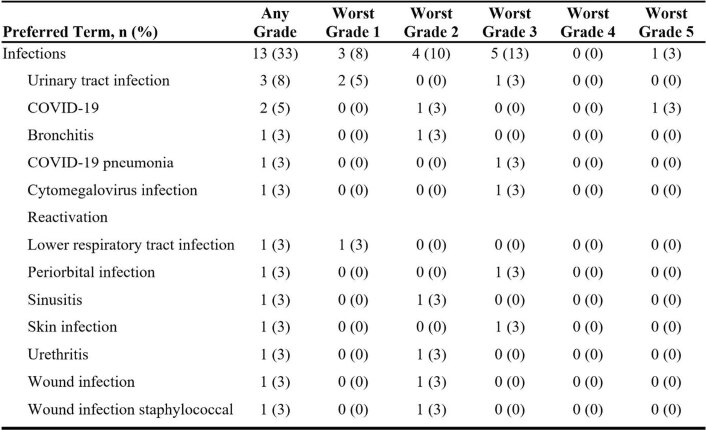
Infections occurring among all treated patients (*n* = 40)

**Extended Data Table 6:**
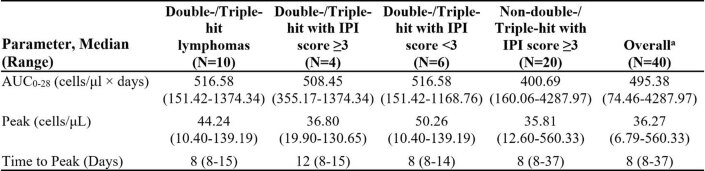
Number of anti-CD19 CAR T cells in blood over time by double- or triple-hit status according to a central laboratory

AUC_0–28_ is defined as the AUC in a plot of number of CAR T cells in blood against scheduled visit from day 0–28. Peak is defined as the maximum number of CAR T cells in blood measured after infusion. Time to peak is defined as the number of days from axicabtagene ciloleucel infusion to the date when the number of CAR T cells in blood first reached the maximum post-baseline level. Patients included are all treated patients, including two in nondouble- or triple-hit with IPI score <3 and eight in double- or triple-hit status not done. CAR, chimeric antigen receptor; IPI, International Prognostic Index.

## Data Availability

Gilead is committed to sharing clinical trial data with external medical experts and scientific researchers in the interest of advancing public health. As such, Gilead shares anonymized individual patient data (IPD) upon request or as required by law and/or regulation. Qualified external researchers may request IPD for studies of Gilead compounds approved in the United States and the European Union with a marketing authorization date on or after 1 January 2014 and that are publicly listed on clinicaltrials.gov or the European Union-Clinical Trials Register. For studies of newly approved compounds or indication, IPD will be available for request 6 months after US Food and Drug Administration (FDA) and European Medicines Agency approval. Such requests are at Gilead’s discretion and are dependent on the nature of the request, the merit of the research proposed, availability of the data and the intended use of the data. If Gilead agrees to the release of clinical data for research purposes, the requester will be required to sign a data-sharing agreement to ensure protection of patient confidentiality before the release of any data. Please contact medinfo@kitepharma.com for all data requests.

## References

[1] *WHO Classification of Tumours of Haematopoietic and Lymphoid Tissue* 4th edn (World Health Organization, 2008).

[2] Sehn LH, Salles G (2021). Diffuse large B-cell lymphoma. New Engl. J. Med..

[3] Ziepert M (2010). Standard International Prognostic Index remains a valid predictor of outcome for patients with aggressive CD20^+^ B-cell lymphoma in the rituximab era. J. Clin. Oncol..

[4] Sehn LH (2007). The revised International Prognostic Index (R-IPI) is a better predictor of outcome than the standard IPI for patients with diffuse large B-cell lymphoma treated with R-CHOP. Blood.

[5] Itti E (2013). An international confirmatory study of the prognostic value of early PET/CT ind diffuse large B-cell lymphoma: comparison between Deauville criteria and DeltaSUVmax. Eur. J. Nucl. Med. Mol. Imaging.

[6] Mamot C (2015). Final results of a prospective evaluation of the predictive value of interim positron emission tomography in patients with diffuse large B-cell lymphoma treated with R-CHOP-14 (SAKK 38/07). J. Clin. Oncol..

[7] Zhu D (2015). Prognostic value of interim (18)F-FDG-PET in diffuse large B cell lymphoma treated with rituximab-based immune-chemotherapy: a systematic review and meta-analysis. Int. J. Clin. Exp. Med..

[8] de Oliveira Costa R (2016). Interim fluorine-18 fluorodeoxyglucose PET-computed tomography and cell of origin by immunohistochemistry predicts progression-free and overall survival in diffuse large B-cell lymphoma patients in the rituximab era. Nucl. Med. Commun..

[9] Barrington SF (2014). Role of imaging in the staging and response assessment of lymphoma: consensus of the International Conference on Malignant Lymphomas Imaging Working Group. J. Clin. Oncol..

[10] Swerdlow SH (2016). The 2016 revision of the World Health Organization classification of lymphoid neoplasms. Blood.

[11] Casasnovas RO (2017). FDG-PET-driven consolidation strategy in diffuse large B-cell lymphoma: final results of a randomized phase 2 study. Blood.

[12] Green TM (2012). Immunohistochemical double-hit score is a strong predictor of outcome in patients with diffuse large B-cell lymphoma treated with rituximab plus cyclophosphamide, doxorubicin, vincristine, and prednisone. J. Clin. Oncol..

[13] Friedberg JW (2017). How I treat double-hit lymphoma. Blood.

[14] Bartlett NL (2019). Dose-adjusted EPOCH-R compared with R-CHOP as frontline therapy for diffuse large B-cell lymphoma: clinical outcomes of the phase III Intergroup Trial Alliance/CALGB 50303. J. Clin. Oncol..

[15] Petrich AM (2014). Impact of induction regimen and stem cell transplantation on outcomes in double-hit lymphoma: a multicenter retrospective analysis. Blood.

[16] Locke FL (2019). Long-term safety and activity of axicabtagene ciloleucel in refractory large B-cell lymphoma (ZUMA-1): a single-arm, multicentre, phase 1-2 trial. Lancet Oncol..

[17] Wang M (2020). KTE-X19 CAR T-cell therapy in relapsed or refractory mantle-cell lymphoma. New Engl. J. Med..

[18] Abramson JS (2020). Lisocabtagene maraleucel for patients with relapsed or refractory large B-cell lymphomas (TRANSCEND NHL 001): a multicentre seamless design study. Lancet.

[19] Schuster SJ (2021). Long-term clinical outcomes of tisagenlecleucel in patients with relapsed or refractory aggressive B-cell lymphomas (JULIET): a multicentre, open-label, single-arm, phase 2 study. Lancet Oncol..

[20] Jacobson CA (2022). Axicabtagene ciloleucel in relapsed or refractory indolent non-Hodgkin lymphoma (ZUMA-5): a single-arm, multicentre, phase 2 trial. Lancet Oncol..

[21] YESCARTA® (axicabtagene ciloleucel) Prescribing information. https://www.fda.gov/media/108377/download (2021).

[22] YESCARTA® (axicabtagene ciloleucel; summary of product characteristics). https://www.ema.europa.eu/en/medicines/human/EPAR/yescarta (2021).

[23] Neelapu SS (2017). Axicabtagene ciloleucel CAR T-cell therapy in refractory large B-cell lymphoma. New Engl. J. Med..

[24] Jacobson C (2021). Long-term (≥4 year and ≥5 year) overall survival (OS) by 12- and 24-month event-free survival (EFS): an updated analysis of ZUMA-1, the pivotal study of axicabtagene ciloleucel (axi-cel) in patients (pts) with refractory large B-cell lymphoma (LBCL). Blood.

[25] Locke F (2020). Tumor burden, inflammation, and product attributes determine outcomes of axi-cel in large B-cell lymphoma. Blood Adv..

[26] Cheson BD (2014). Recommendations for initial evaluation, staging, and response assessment of Hodgkin and non-Hodgkin lymphoma: the Lugano classification. J. Clin. Oncol..

[27] International Non-Hodgkin’s Lymphoma Prognostic Factors Project. (1993). A predictive model for aggressive non-Hodgkin’s lymphoma. New Engl. J. Med..

[28] Crump M (2017). Outcomes in refractory diffuse large B-cell lymphoma: results from the international SCHOLAR-1 study. Blood.

[29] Neelapu, S. S. et al. Comparison of 2-year outcomes with CAR T cells (ZUMA-1) versus salvage chemotherapy in refractory large B-cell lymphoma. *Blood Adv.* **5**, 4149–4155 (2021). 10.1182/bloodadvances.2020003848 PMC8945634 34478487

[30] Locke, F. L. et al. Axicabtagene ciloleucel as second-line therapy for large B-cell lymphoma. *New Engl. J. Med.*10.1056/NEJMoa2116133 (2021). 10.1056/NEJMoa2116133 34891224

[31] Kamdar MK (2021). Lisocabtagene maraleucel (liso-cel), a CD19-directed chimeric antigen receptor (CAR) T cell therapy, versus standard of care (SOC) with salvage chemotherapy (CT) followed by autologous stem cell transplantation (ASCT) as second-line (2L) treatment in patients (pts) with relapsed or refractory (R/R) large B-cell lymphoma (LBCL): results from the randomized phase 3 Transform study. Blood.

[32] Bishop, M. R. et al*.* Second-line tisagenlecleucel or standard care in aggressive B-cell lymphoma. *New Engl. J. Med.*10.1056/NEJMoa2116596 (2021). 10.1056/NEJMoa2116596 34904798

[33] Roschewski, M., Longo, D. L. & Wilson, W. H. CAR T-cell therapy for large B-cell lymphoma – who, when, and how? *New Engl. J. Med.*10.1056/NEJMe2118899 (2021). 10.1056/NEJMe2118899 PMC9295142 34904797

[34] Schoder H (2020). Prognostic value of interim FDG-PET in diffuse large cell lymphoma: results from the CALGB 50303 Clinical Trial. Blood.

[35] Topp MS (2015). Safety and activity of blinatumomab for adult patients with relapsed or refractory B-precursor acute lymphoblastic leukaemia: a multicentre, single-arm, phase 2 study. Lancet Oncol..

[36] Lee DW (2014). Current concepts in the diagnosis and management of cytokine release syndrome. Blood.

[37] Locke FL (2017). Phase 1 results of ZUMA-1: a multicenter study of KTE-C19 anti-CD19 CAR T cell therapy in refractory aggressive lymphoma. Mol. Ther..

