# Supplementary material for: Axicabtagene ciloleucel as first-line therapy in high-risk large B-cell lymphoma: the phase 2 ZUMA-12 trial
Source: Nat Med. 2022 Mar 21;28(4):735–42. doi: 10.1038/s41591-022-01731-4 (PMC9018426; doi:10.1038/s41591-022-01731-4)
Supplement: Supplementary file 1 — Supplementary Fig. 1, and redacted study protocol and SAP. [file 41591_2022_1731_MOESM1_ESM.pdf]

nature portfolio

https://doi.org/10.1038/s41591-022-01731-4

Supplementary information

Axicabtagene ciloleucel as first-line therapy in high-risk large B-cell lymphoma: the phase 2 ZUMA-12 trial

In the format provided by the authors and unedited

Supplemental Figure 1. CD19 CAR T cell phenotype gating strategy

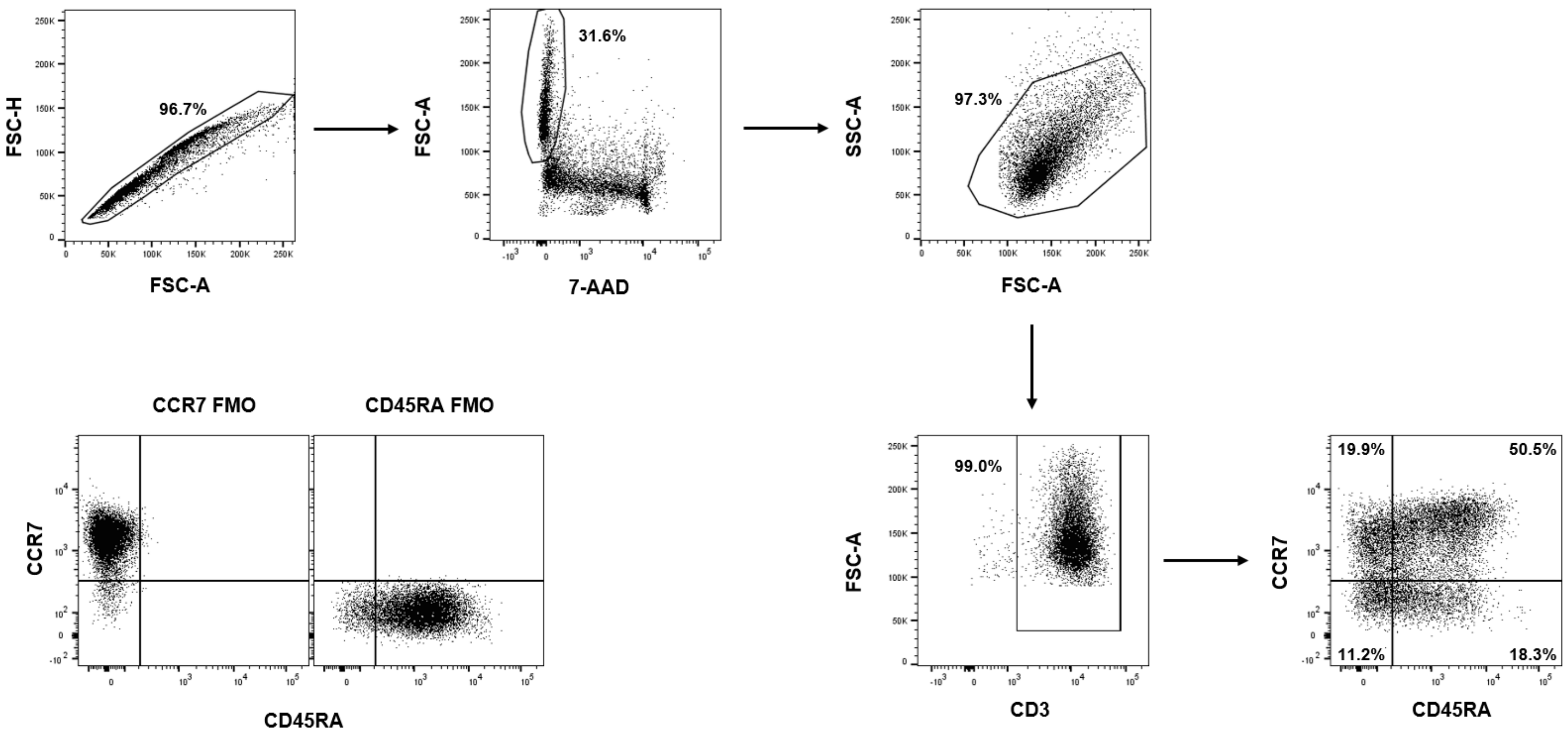

Kite, a Gilead Company

Redacted Clinical Trial Protocol for Journal Use

A Phase 2 Multicenter Study Evaluating the Efficacy and Safety of Axicabtagene Ciloleucel as First-Line Therapy in Subjects with High-Risk Large B-Cell Lymphoma (ZUMA-12)

NCT03761056
EudraCT Number: 2019-002291-13
Phase 2
FINAL PROTOCOL
KTE-C19-112

**Original Protocol Date:**	26 June 2018
**Final Protocol Date:**	14 August 2019
**Prepared by:**	Kite, a Gilead Company, 2400 Broadway, Santa Monica, CA 90404

Figure 1. **Study Schema**

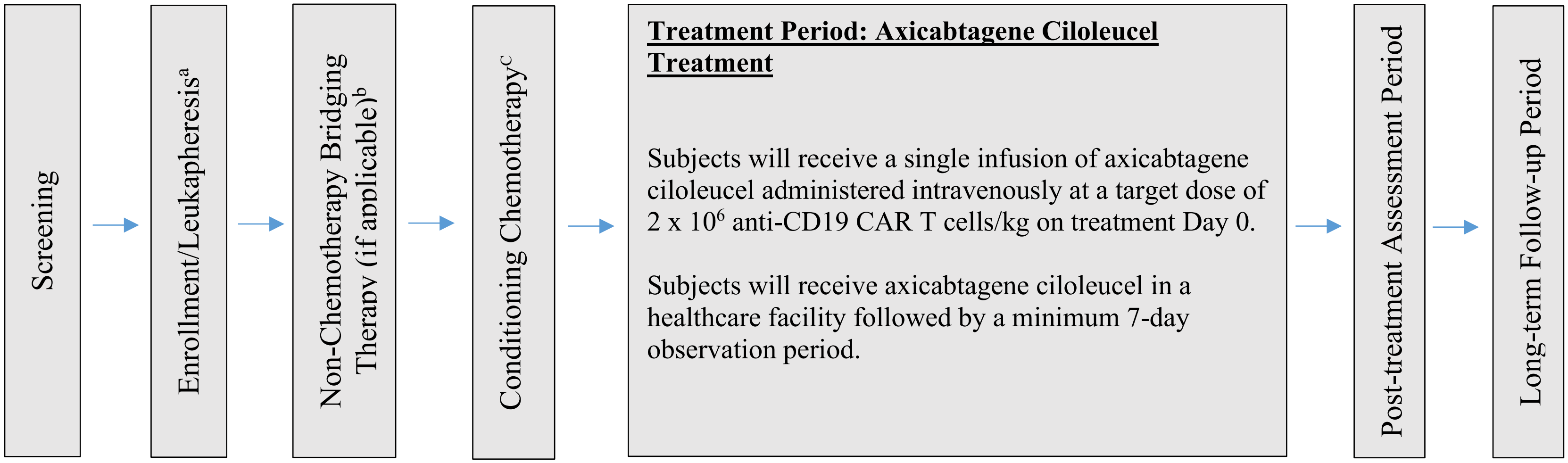

Approximately 40 subjects who are either double hit/triple hit or have IPI ≥ 3 will be enrolled and treated.

[a] **Enrollment/Leukapheresis:** Subjects who have a positive interim PET per the Lugano Classification {Cheson 2014} (Deauville PET score of 4 or 5) after 2 cycles (PET2+) of an anti-CD20 monoclonal antibody and anthracycline-containing regimen per local standard of care (eg, DA-EPOCH-R) if double hit/triple hit, or an anti-CD20 monoclonal antibody and anthracycline-containing regimen per local standard of care (eg, R-CHOP) if large B-cell lymphoma with IPI score ≥ 3.

[b] **Non-Chemotherapy Bridging Therapy:** At the discretion of the investigator, corticosteroid or HDMP + rituximab bridging therapy may be considered for subjects with high disease burden at screening or baseline assessments. Localized radiation therapy for symptom control may also be allowed, provided that the radiation field does not include a target lesion. Refer to Section 6.1.2 for details.

[c] **Conditioning Chemotherapy:** Subjects will receive a 3-day conditioning chemotherapy regimen consisting of fludarabine 30 mg/m^2/day and cyclophosphamide 500 mg/m^2/day (Day –5 to Day –3) followed by 2 rest days (Day –2 and Day –1).

TABLE OF CONTENTS

TABLE OF CONTENTS 3

LIST OF TABLES 5

LIST OF FIGURES 5

LIST OF ABBREVIATIONS 6

1. OBJECTIVES 8
   - 1.1. Primary Objective 8
   - 1.2. Secondary Objective(s) 8
   - 1.3. Study Endpoints 8
     - 1.3.1. Primary Endpoint 8
     - 1.3.2. Secondary Endpoints 8

2. STUDY DESIGN AND RATIONALE 10
   - 2.1. General Study Design 10
   - 2.2. Study Design Rationale 11
   - 2.3. Participating Sites 12
   - 2.4. Number of Subjects 12
   - 2.5. Replacement of Subjects 12
   - 2.6. Study Duration 12
     - 2.6.1. Study Duration for Individual Subjects 12
     - 2.6.2. Completion of Study 12

3. SUBJECT IDENTIFICATION ASSIGNMENT 13

4. SUBJECT ELIGIBILITY 14
   - 4.1. Inclusion Criteria 14
   - 4.2. Exclusion Criteria 15

5. PROTOCOL TREATMENT 18
   - 5.1. Study Treatment 18
     - 5.1.1. Leukapheresis 18
     - 5.1.2. Non-Chemotherapy Bridging Therapy 18
     - 5.1.3. Conditioning Chemotherapy 18
     - 5.1.4. Axicabtagene Ciloleucel 19
     - 5.1.5. Concomitant Therapy 19
     - 5.1.6. Excluded Medications 20
     - 5.1.7. Subsequent Therapy 21
     - 5.1.8. Toxicity Management 21

6. STUDY PROCEDURES 22
   - 6.1. Informed Consent 22
   - 6.2. Screening 22
     - 6.2.1. Rescreening 23
   - 6.3. Demographic Data 23
   - 6.4. Medical and Treatment History 23
   - 6.5. Conditioning Chemotherapy and Axicabtagene Ciloleucel Infusion 23
     - 6.5.1. Requirements for Initiating Conditioning Chemotherapy 24
     - 6.5.2. Requirements for Initiating Axicabtagene Ciloleucel Infusion 25
     - 6.5.3. Requirements to Work-up Potential Infectious and/or Inflammatory States 26

6.5.4. Conditioning Chemotherapy Administration (Day –5 Through Day –3 Prior to Axicabtagene Ciloleucel Infusion) 27
6.6. Physical Exam, Vital Signs, and Performance Status 27
6.7. Neurological Examination 27
6.8. Disease Assessment 28
6.8.1. Imaging 28
6.8.2. Determination of Bone Marrow Involvement 29
6.9. Cell Collection and Axicabtagene Ciloleucel Study Treatment Schedule and Administration 29
6.9.1. Leukapheresis 29
6.9.2. Non-Chemotherapy Bridging Therapy 30
6.9.3. Axicabtagene Ciloleucel Treatment Period 30
6.10. Post-treatment Assessment Period 32
6.11. Long-term Follow-up Period 32

7. SUBJECT WITHDRAWAL 34

7.1. Reasons for Removal from Treatment 34
7.2. Reasons for Removal from Study 34

8. SAFETY REPORTING 35

8.1. Adverse Events 35
8.2. Reporting of Adverse Events 35
8.3. Definition of Serious Adverse Events 36
8.4. Reporting of Serious Adverse Events 37
8.5. Reporting Deaths 38
8.6. Diagnosis Versus Signs and Symptoms 38
8.7. Pregnancy and Lactation 38
8.8. Hospitalization and Prolonged Hospitalization 39
8.9. Abnormal Vital Sign Values 39
8.10. Data Safety Monitoring Board 39

9. STATISTICAL CONSIDERATIONS 41

9.1. Hypothesis 41
9.1.1. Covariates and Subgroups 41
9.2. Sample Size Considerations 41
9.3. Access to Individual Subject Treatment Assignments 42
9.4. Interim Analysis and Early Stopping Guidelines 42
9.5. Analysis Subsets 42
9.6. Planned Method of Analysis 43
9.6.1. CR Rate 43
9.6.2. ORR 43
9.6.3. DOR 43
9.6.4. EFS 44
9.6.5. PFS 44
9.6.6. OS 44
9.6.7. Relapse with CNS Disease 44
9.6.8. Safety 44
9.6.9. Pharmacokinetic Analysis 45
9.6.10. Pharmacodynamics Analyses 45

10. REFERENCES 46

11. APPENDICES 48

Appendix 1. Lugano Classification {Cheson 2014} 49

Appendix 2. International Prognostic Index in Aggressive Lymphomas.....53
Appendix 3. Birth Control Methods Which May Be Considered as Highly Effective.....54

LIST OF TABLES

Table 1. Lower and Upper Limits of 80%/95% CIs for CR Rates from 60% to 100% for a Sample of 40 Subjects.....42

LIST OF FIGURES

Figure 1. Study Schema.....2

LIST OF ABBREVIATIONS

Abbreviation	Definition
5PS	Five-point scale
ABC	activated B-cell
AE	adverse event
autoSCT	Autologous stem cell transplant
CAR	chimeric antigen receptor
CBC	complete blood count
CD	cluster of differentiation
CHOP	cyclophosphamide, doxorubicin, vincristine, prednisone
CI	confidence interval
CNS	central nervous system
CR	complete response
CRF	case report form
CRP	C-reactive protein
CRS	cytokine release syndrome
CSR	clinical study report
CT	Computed Tomography
CTCAE	Common Terminology Criteria For Adverse Events
DA-EPOCH-R	dose-adjusted etoposide, prednisone, vincristine, cyclophosphamide, doxorubicin, rituximab
DH	double- hit
DLBCL	diffuse large B-cell lymphoma
DOR	duration of response
DSMB	Data Safety Monitoring Board
ECHO	Echocardiogram
ECOG	Eastern Cooperative Oncology Group
eCRF	electronic case report form
EFS	event-free survival
FDA	Food and Drug Administration
FDG	fluorodeoxyglucose
GCB	Germinal Center B-cell
GELA	Groupe d'Etude des Lymphomes de l'Adulte
HIV	human immunodeficiency virus
HDMP	high-dose methylprednisolone
HEENT	Head, eyes, ears, nose, and throat
HGBL	high-grade B-cell lymphoma
HR	heart rate
ID	Identification
ICF	informed consent form

IPI	International Prognostic Index
IRB/IEC	institutional review board/independent ethics committee
IRC	independent review committee
IV	Intravenous
IWG	international working group
LDi	longest transverse diameter
NCI	National Cancer Institute
NHL	Non-Hodgkin lymphoma
NOS	Not otherwise specified
ORR	Objective response rate
OS	Overall survival
PBMC	Peripheral blood mononuclear cell
PCR	Polymerase chain reaction
PD	Progressive disease
PET-CT	Positron emission tomography–computed tomography
PFS	Progression-free survival
PK	Pharmacokinetic
PMBCL	Primary mediastinal B-cell lymphoma
PR	Partial response
PFS	Progression-free survival
R-CHOP	Rituximab, cyclophosphamide, doxorubicin, vincristine, prednisone
R-ACVBP	Rituximab, doxorubicin, cyclophosphamide, vindesine, bleomycin, prednisone
RCR	Replication-competent retrovirus
SAE	Serious adverse event
SCT	Stem cell transplant
SDi	Shortest transverse diameter
SUSAR	Suspected unexpected serious adverse reactions
ULN	Upper limit of normal
WBC	White blood cell
WHO	World Health Organization

1. OBJECTIVES

1.1. Primary Objective

To estimate the efficacy of axicabtagene ciloleucel, as measured by CR rate, in subjects with high-risk large B-cell lymphoma, as determined by study investigators

1.2. Secondary Objective(s)

To characterize the safety profile, and to further characterize efficacy with secondary endpoints; further secondary objectives will include pharmacokinetic/pharmacodynamic endpoints

1.3. Study Endpoints

1.3.1. Primary Endpoint

CR rate is defined as the incidence of a CR per the Lugano Classification {Cheson 2014} as determined by study investigators. All evaluable subjects who do not meet the criteria for a CR by the analysis data cutoff date will be considered non-responders.

1.3.2. Secondary Endpoints

ORR is defined as the incidence of either a CR or a PR per the Lugano Classification {Cheson 2014} as determined by the study investigators. All evaluable subjects who do not meet the criteria for an objective response by the analysis cutoff date will be considered non-responders.

DOR is defined only for subjects who experience an objective response after axicabtagene ciloleucel infusion and is the time from the first objective response to disease progression per the Lugano Classification {Cheson 2014} or death from any cause. Subjects not meeting the criteria for disease progression or death from any cause by the analysis data cutoff date will be censored at their last evaluable disease assessment date. Subjects who receive any subsequent new antilymphoma therapy, including SCT in the absence of documented disease progression or death, will be censored at the last evaluable disease assessment prior to the subsequent new antilymphoma therapy.

EFS is defined as the time from the axicabtagene ciloleucel infusion date to the earliest date of disease progression per the Lugano Classification {Cheson 2014}, commencement of subsequent new antilymphoma therapy including SCT, or death from any cause. Subjects alive, in response, and with no new antilymphoma therapy including SCT will be censored at the last evaluable disease assessment.

PFS is defined as the time from the axicabtagene ciloleucel infusion date to the date of disease progression per the Lugano Classification {Cheson 2014} or death from any cause. Subjects not meeting the criteria for disease progression or death from any cause by the analysis data cutoff date will be censored at the last evaluable disease assessment. Subjects who receive any subsequent new antilymphoma therapy including SCT in the absence of documented disease

progression or death will be censored at the last evaluable disease assessment prior to the subsequent new antilymphoma therapy.

OS is defined as the time from axicabtagene ciloleucel infusion to the date of death from any cause. Subjects who are alive will be censored at their last date known to be alive or the data cutoff date, whichever is earlier. Subjects who die after the data cutoff date will be censored at the data cutoff date.

Relapse with CNS disease is defined as the time from the axicabtagene ciloleucel infusion date to the earliest date of CNS involvement with lymphoma, as determined by typical symptoms, CSF evaluation, and/or diagnostic imaging.

Incidence of AEs (including Grade 3 or higher, serious, fatal, and AEs of interest) and clinically significant changes in safety laboratory values. Additional secondary endpoints include PK and pharmacodynamic endpoints, such as the evaluation of anti-CD19 CAR T-cell levels in the blood and cytokine levels in the serum in relationship with clinical outcome.

2. STUDY DESIGN AND RATIONALE

2.1. General Study Design

Study KTE-C19-112 (ZUMA-12) is a Phase 2 multicenter, open-label study evaluating the efficacy and safety of axicabtagene ciloleucel as first-line therapy in adult subjects with high-risk large B-cell lymphoma, including either HGBL with *MYC* and *BCL2* and/or *BCL6* translocations (double-/triple-hit lymphomas) or large B-cell lymphomas with high-intermediate-/high-risk IPI scores (≥ 3). Approximately 40 subjects will be enrolled upon a positive interim PET per the Lugano Classification {Cheson 2014} (Deauville 5-point scale [5PS] PET score of 4 or 5) after 2 cycles (PET2+) of standard-of-care chemoimmunotherapy.

Subjects will proceed through the following study periods:

- Screening
- Enrollment/Leukapheresis
- Non-chemotherapy bridging therapy (if applicable)
- Conditioning chemotherapy period
- Investigational product treatment period
- Post-treatment assessment period
- Long-term follow-up period

An independent Data Safety Monitoring Board (DSMB) will be chartered to meet and review the serious adverse events (SAEs) and suspected unexpected serious adverse reactions (SUSARs) on a semi-annual basis after the first subject has been treated with axicabtagene ciloleucel up through the primary analysis. Kite Pharma, Inc., or delegate, will submit SAEs and SUSARs to the DSMB on a regular basis throughout the study up through the primary analysis. The DSMB will also meet to review safety data after 15 subjects have been enrolled and treated with axicabtagene ciloleucel and have had an opportunity to be followed for 3 months after axicabtagene ciloleucel infusion. The DSMB will make trial conduct recommendations on an ongoing basis based on an analysis of risk versus benefit. The DSMB may request additional safety data for review or recommend modifications to the study conduct if safety concerns are identified. Refer to Section 9.10 for further details.

A study schema is described in Figure 1.

2.2. Study Design Rationale

Study KTE-C19-112 (ZUMA-12) is an open-label, single-arm study evaluating the efficacy and safety of axicabtagene ciloleucel as first-line therapy in adult subjects with high-risk large B-cell lymphoma (ie, double-/triple-hit lymphomas or large B-cell lymphoma with IPI scores ≥ 3).

Patients with high-risk large B-cell lymphoma have an increased risk of relapse and death due to disease progression after first-line rituximab-based chemoimmunotherapy. This was recently demonstrated in a randomized study in high-risk DLBCL patients who had a change of therapy based on interim PET results {Casasnovas 2017}. LNH 2007-3B was a GELA randomized Phase 2 study that evaluated the efficacy of either R-ACVBP or R-CHOP-14 induction and a PET-driven autologous stem cell transplant (autoSCT) or standard chemoimmunotherapy consolidation in young patients (18 to 59 years old) with high-risk DLBCL and 2 or 3 risk factors (age-adjusted IPI 2 or 3). PET was performed at baseline, after 2 (PET2) and 4 (PET4) induction cycles, with central review. At the end of induction treatment, the CR rate according to IWG 2007 criteria {Cheson 2014} was 47% (51 of 109 subjects) with R-ACVBP (95% CI, 38% to 67%) and 39% (40 of 102 subjects) with R-CHOP-14 (95% CI, 28% to 54%; P = 0.76). The primary objective of achieving a higher than 50% CR rate after 4 cycles of induction was not met in either randomization group. In this study, PET2 was positive in 66% (72 of 109 subjects) and 71% (72 of 102 subjects) in the R-ACVBP and the R-CHOP-14 groups, respectively {Casasnovas 2017}. Based on the results of the LNH 2007-3B study, as well as several earlier studies which assessed the role of early PET-CT {de Oliveira Costa 2016, Itti 2013, Mamot 2015, Zhu 2015}, patients with high-risk large B-cell lymphoma who also have not achieved CR based on dynamic PET-CT assessment may have a worse prognosis and represent a subgroup with a high unmet medical need. Because these patients are at higher risk of becoming resistant to chemoimmunotherapy, they may benefit from therapies with different mechanisms of action.

Immunotherapy, which is based on the enhancement of an immune response against the tumor, is a promising approach to treating many cancer types. T cells play an important role in destroying diseased cells throughout the body. Studies with immune checkpoint inhibitors and bi-specific T-cell engagers have demonstrated the potential of T cells to treat cancer. T cells need to possess the appropriate specificity for a tumor, be present in sufficient numbers, and overcome any local immunosuppressive factors to be effective. CAR-engineered T cells may address these issues and are an approach for cancer therapy.

Axicabtagene ciloleucel is an engineered autologous T-cell immunotherapy in which a patient's own T cells are collected and subsequently genetically altered to recognize CD19. CD19 is expressed on the cell surface of B-cell malignancies and normal B cells. In the pivotal ZUMA-1 Phase 1/2 trial, the safety and efficacy of axicabtagene ciloleucel were evaluated in patients with refractory DLBCL, primary mediastinal B-cell lymphoma (PMBCL), and transformed follicular lymphoma. In ZUMA-1, the ORR was 72% with a CR rate of 51%, as determined by an IRC per IWG 2007 criteria {Cheson 2007} and met the primary endpoint {YESCARTA 2019}. Further, in ZUMA-1, the ongoing durable remissions have been comparable across key covariates including IPI and gene expression profiling of ABC and GCB subtypes {Neelapu 2017}. Similar improved outcomes might be expected in double-/triple-hit lymphoma and in high and high-intermediate risk IPI.

Axicabtagene ciloleucel may also have improved efficacy and tolerability in patients with less chemo-refractory disease or in patients with lower disease burden. In ZUMA-1, the rate of ongoing responses in an exploratory quartile analysis of tumor burden decreased with increasing tumor burden as determined by sum of product diameters of target lesions. At the 12 month follow-up, 67% ongoing response was observed in the first quartile compared to 27% for the fourth quartile. On the other hand, Grade 3 or higher CRS and neurologic events occurred less frequently in patients with lower quartiles of tumor burden: 4% and 7%, respectively, for the first quartile compared to 12% and 31%, respectively, for the fourth quartile {Locke 2018}.

Patients with double-/triple-hit lymphoma or high-intermediate-/high-risk IPI (IPI score ≥ 3) have a high unmet medical need. Therefore, axicabtagene ciloleucel will be assessed as first-line treatment in these high-risk large B-cell lymphoma patients who are also PET positive after 2 cycles (PET2+) of standard-of-care chemoimmunotherapy.

2.3. Participating Sites

Approximately 10 centers located in North America and the European Union will participate in this study and other regions may be considered. During the conduct of the study, additional regions, countries, or sites may be added as necessary.

2.4. Number of Subjects

Participants in this trial will be referred to as "subjects." It is anticipated that approximately 40 subjects will be enrolled and treated into this study.

2.5. Replacement of Subjects

Subjects may continue to be enrolled until the specified number of subjects are dosed with axicabtagene ciloleucel. Subjects who have not received axicabtagene ciloleucel will be retained in the analyses of disposition and safety, where appropriate (see Section 10.6).

2.6. Study Duration

2.6.1. Study Duration for Individual Subjects

The duration of the study for individual subjects will vary. For a subject who completes the entire protocol from the date of informed consent through the completion of the long-term follow-up period, the duration of the study will take approximately 15 years to complete. However, individual study duration will vary depending on a subject's screening requirements, response to treatment, and survival.

2.6.2. Completion of Study

Completion of the study is defined as the time at which the last subject completes the long-term follow-up period visit, is lost to follow-up, withdraws consent, or dies.

3. SUBJECT IDENTIFICATION ASSIGNMENT

Each subject who enters the screening period, which starts when the subject signs the informed consent form (ICF), will receive a unique subject identification (ID) number. This number will be used to identify the subject throughout the study and must be used on all study documentation related to the subject. The subject identification number will never be changed even if the subject is rescreened.

4. SUBJECT ELIGIBILITY

4.1. Inclusion Criteria

101. Large B-cell lymphoma with one or more of the following **features**:

- HGBL with *MYC* and *BCL2* and/or *BCL6* translocations (double-hit or triple-hit) as determined by investigator by fluorescent in situ hybridization

  OR

- Other histologically confirmed large B-cell lymphoma defined by WHO 2016 {Swerdlow 2016} with an IPI score of ≥ 3 at initial diagnosis or anytime between initial diagnosis and enrollment, including the following lymphoma types:

  — DLBCL-NOS, including GCB type and ABC type

  — Intravascular large B-cell lymphoma

  — T-cell/histiocyte-rich large B-cell lymphoma

  — DLBCL associated with chronic inflammation

  — Epstein-Barr virus + DLBCL-NOS

  — HGBL-NOS

102. Subjects must have a positive interim PET per the Lugano Classification {Cheson 2014}(Deauville PET score of 4 or 5) after 2 cycles (PET2+) of chemoimmunotherapy as follows:

- 2 cycles of an anti-CD20 monoclonal antibody (unless investigator determines that tumor is CD20 negative) and anthracycline-containing regimen (eg, DA-EPOCH-R), with or without intrathecal chemotherapy, at the discretion of the investigator per local standard of care for double-hit or triple-hit lymphoma

  OR

- 2 cycles of an anti-CD20 monoclonal antibody (unless investigator determines that tumor is CD20 negative) and anthracycline-containing regimen (eg, R-CHOP) at the discretion of the investigator per local standard of care for large B-cell lymphoma with IPI score of ≥ 3

103. At least 2 weeks must have elapsed since any prior systemic therapy at the time the subject is planned for leukapheresis

104. No evidence, suspicion, and/or history of CNS involvement of lymphoma

105. Toxicities due to prior therapy must be stable and recovered to Grade 1 or less (except for clinically non-significant toxicities such as alopecia)

106. Age 18 or older

107. Eastern Cooperative Oncology Group (ECOG) performance status of 0 or 1

108. Adequate bone marrow, renal, hepatic, pulmonary, and cardiac function defined as:

- Absolute neutrophil count ≥ 1000/μL
- Platelet count ≥ 75,000/μL
- Absolute lymphocyte count ≥ 100/μL
- Creatinine clearance (as estimated by Cockcroft Gault) ≥ 60 mL/min
- Serum alanine aminotransferase and aspartate aminotransferase ≤ 2.5 upper limit of normal (ULN)
- Total bilirubin ≤ 1.5 mg/dL, except in subjects with Gilbert's syndrome
- Cardiac ejection fraction ≥ 50% , no evidence of pericardial effusion (except trace or physiological) as determined by an echocardiogram (ECHO), and no clinically significant electrocardiogram findings
- No clinically significant pleural effusion
- Baseline oxygen saturation > 92% on room air

109. Females of childbearing potential must have a negative serum or urine pregnancy test (females who have undergone surgical sterilization or who have been postmenopausal for at least 2 years are not considered to be of childbearing potential).

4.2. Exclusion Criteria

201. History of malignancy other than nonmelanoma skin cancer or carcinoma in situ (eg, cervix, bladder, breast) unless disease free for at least 3 years

202. History of Richter's transformation of chronic lymphocytic leukemia or PMBCL

203. History of autologous or allogeneic SCT

204. Prior CD19-targeted therapy

205. Prior CAR therapy or other genetically modified T-cell therapy

206. History of severe, immediate hypersensitivity reaction attributed to aminoglycosides

207. Presence or suspicion of fungal, bacterial, viral, or other infection that is uncontrolled or requiring IV antimicrobials for management; simple urinary tract infection and uncomplicated bacterial pharyngitis are permitted if responding to active treatment and after consultation with the sponsor's medical monitor

208. History of human immunodeficiency virus (HIV) infection or acute or chronic active hepatitis B or C infection; subjects with history of hepatitis infection must have cleared their infection as determined by standard serological and genetic testing per current Infectious Diseases Society of America guidelines or applicable country guidelines

209. Presence of any indwelling line or drain (eg, percutaneous nephrostomy tube, indwelling Foley catheter, biliary drain, or pleural/peritoneal/pericardial catheter); dedicated central venous access catheters, such as a Port-A-Cath® or Hickman® catheter, are permitted

210. Subjects with detectable cerebrospinal fluid malignant cells, brain metastases, or active CNS lymphoma

211. History or presence of CNS disorder, such as seizure disorder, cerebrovascular ischemia/hemorrhage, dementia, cerebellar disease, or any autoimmune disease with CNS involvement

212. Subjects with cardiac atrial or cardiac ventricular lymphoma involvement

213. History of myocardial infarction, cardiac angioplasty or stenting, unstable angina, or other clinically significant cardiac disease within 12 months of enrollment

214. Requirement for urgent therapy due to tumor mass effects (eg, blood vessel compression, bowel obstruction, or transmural gastric involvement)

215. Primary immunodeficiency

216. History of autoimmune disease (eg, Crohns, rheumatoid arthritis, systemic lupus) resulting in end organ injury or requiring systemic immunosuppression/systemic disease modifying agents within the last 2 years

217. History of symptomatic deep vein thrombosis or pulmonary embolism within 6 months of enrollment

218. Any medical condition likely to interfere with assessment of safety or efficacy of study treatment

219. History of severe immediate hypersensitivity reaction to any of the agents used in this study

220. Live vaccine ≤ 6 weeks prior to planned start of conditioning regimen

221. Women of childbearing potential who are pregnant or breastfeeding because of the potentially dangerous effects of the preparative chemotherapy on the fetus or infant. Females who have undergone surgical sterilization or who have been postmenopausal for at least 2 years are not considered to be of childbearing potential

222. Subjects of both genders who are not willing to practice birth control from the time of consent through 6 months after the completion of conditioning chemotherapy or axicabtagene ciloleucel infusion, whichever is longer

223. In the investigator's judgment, the subject is unlikely to complete all protocol-required study visits or procedures, including follow-up visits, or comply with the study requirements for participation

5. PROTOCOL TREATMENT

5.1. Study Treatment

5.1.1. Leukapheresis

Leukapheresis refers to the procedure for collecting peripheral blood mononuclear cells (PBMCs) that are used to manufacture axicabtagene ciloleucel.

Subjects will undergo leukapheresis to obtain T cells for the manufacturing of axicabtagene ciloleucel. Leukapheresed cells obtained at participating centers will be shipped to the sponsor's manufacturing facility.

At least 2 weeks must have elapsed since any prior systemic therapy at the time the subject is planned for leukapheresis.

5.1.2. Non-Chemotherapy Bridging Therapy

Non-chemotherapy bridging therapy will be supplied by the investigative site unless otherwise noted. Sites should refer to the current product label for guidance on packaging, storage, preparation, administration, and toxicity management of non-chemotherapy bridging therapy.

At the discretion of the investigator, non-chemotherapy bridging therapy may be considered for subjects with high disease burden at screening or baseline assessments (eg, bulky disease or rapidly progressing disease).

Localized radiation therapy for symptom control may also be allowed provided that the radiation field does not include a target lesion.

5.1.3. Conditioning Chemotherapy

Conditioning chemotherapy refers to fludarabine and cyclophosphamide used for lymphodepletion prior to administration of axicabtagene ciloleucel.

Conditioning chemotherapy will be supplied by the investigative site unless otherwise noted.

Refer to the current product label for guidance on packaging, storage, preparation, administration, and toxicity management associated with the administration of chemotherapy agents.

5.1.3.1. Fludarabine

Fludarabine phosphate is a synthetic purine nucleoside that differs from physiologic nucleosides in that the sugar moiety is arabinose instead of ribose or deoxyribose. Fludarabine is a purine antagonist antimetabolite.

Refer to the most recent version of the package insert for specific details surrounding the administration of fludarabine.

5.1.3.2. Cyclophosphamide

Cyclophosphamide is a nitrogen mustard-derivative that acts as an alkylating agent following conversion to active metabolites in the liver and has potent immunosuppressive activity. The serum half-life after IV administration ranges from 3 to 12 hours; the drug and/or its metabolites can be detected in the serum for up to 72 hours after administration.

Refer to the most recent version of the package insert for specific details surrounding the administration of cyclophosphamide.

5.1.3.3. Mesna

Mesna is a detoxifying agent used to inhibit the hemorrhagic cystitis induced by chemotherapy. The active ingredient in mesna is a synthetic sulfhydryl compound designated as sodium-2-mercaptoethane sulfonate with a molecular formula of $C_2H_5NaO_3S_2$.

Mesna should be administered per institutional guidelines. Refer to the most recent version of the package insert for specific details surrounding the administration of mesna.

5.1.4. Axicabtagene Ciloleucel

Axicabtagene ciloleucel is the investigational product for this study.

Axicabtagene ciloleucel is a subject-specific product. The product is labelled per local regulations with the subject's unique subject ID number assigned at the time of screening. Upon receipt, verification that the product and subject-specific labels match the subject's information (eg, subject ID number) is essential. Do not infuse the product if the information on the subject-specific label does not match the intended subject. The volume of axicabtagene ciloleucel infused, the thaw start/stop time, and axicabtagene ciloleucel administration start/stop time will all be noted in the subject's medical record. The product must not be thawed until the subject is ready for the infusion.

There have been no instances of accidental overdose of subjects in this program to date. In case of accidental overdose, treatment should be supportive. Corticosteroid therapy may be considered if any dose is associated with severe toxicity.

If any problems related to the use of axicabtagene ciloleucel or any products that support the management of axicabtagene ciloleucel are identified, research staff should report the problem.

5.1.5. Concomitant Therapy

Concomitant therapy refers to treatment that subjects receive during the conduct of the study.

During the course of the study, investigators may prescribe any concomitant therapies deemed necessary to provide adequate supportive care except those medications listed in Section 5.1.6.

All concomitant therapies, including medications, intubation, dialysis, oxygen, and blood products, will be recorded.

For subjects who receive axicabtagene ciloleucel treatment:

- Concomitant therapies will be recorded from the date of the informed consent until 3 months after completing treatment with axicabtagene ciloleucel.
- After this 3-month follow-up period, targeted concomitant therapies will be recorded for either 24 months after axicabtagene ciloleucel infusion or until disease progression, whichever occurs first. Targeted concomitant therapies include gammaglobulin, immunosuppressive drugs, anti-infective drugs, and vaccinations.

For subjects who are enrolled, but not dosed with axicabtagene ciloleucel, concomitant therapies will be recorded from the date of the informed consent until 30 days after the last study-specific procedure has occurred (eg, leukapheresis, conditioning chemotherapy) or until the initiation of new antilymphoma therapy, whichever occurs first.

For subjects who are not enrolled (eg, screen failure), only concomitant therapies related to any serious adverse event(s) (SAE[s]) will be recorded.

Specific concomitant therapy collection requirements and instructions are included in the case report form (CRF) completion guidelines.

5.1.6. Excluded Medications

Excluded medications refer to treatment that is not to be administered, unless otherwise specified, during the conduct of the study.

Corticosteroid therapy at a pharmacologic dose (≥ 5 mg/day of prednisone or equivalent doses of other corticosteroids) and other immunosuppressive drugs must be avoided for 7 days prior to leukapheresis and 5 days prior to axicabtagene ciloleucel administration.

Systemic corticosteroids may not be administered as premedication to subjects for whom CT scans with contrast are contraindicated (ie, subjects with contrast allergy or impaired renal clearance). Such subjects should undergo non-contrast CT scans instead.

Corticosteroids and other immunosuppressive drugs should also be avoided for 3 months after axicabtagene ciloleucel administration unless used to manage axicabtagene ciloleucel-related toxicities. Other medications that might interfere with the evaluation of axicabtagene ciloleucel, such as nonsteroidal anti-inflammatory agents, should also be avoided for the same period unless medically necessary.

Therapeutic doses of systemic anticoagulants, such as unfractionated heparin and low-molecular weight heparin, should be avoided anytime subjects are at risk of bleeding due to thrombocytopenia when possible.

Treatments for lymphoma, such as chemotherapy, immunotherapy, targeted agents, radiation, and high-dose corticosteroids (other than those defined/allowed in this protocol) and other investigational agents, are prohibited, except as needed for treatment of disease progression after axicabtagene ciloleucel.

If permissibility of a specific medication/treatment is in question, contact the sponsor's medical monitor.

5.1.7. Subsequent Therapy

Subsequent therapy refers to treatment administered after axicabtagene ciloleucel that is necessary to treat a subject's disease.

Subsequent therapy administered after axicabtagene ciloleucel that is necessary to treat a subject's disease, such as non-study specified chemotherapy, immunotherapy, targeted agents, SCT, or radiation therapy, will be recorded for subjects until one of the following happens: the subject completes the long-term follow-up period, is considered lost to follow-up, withdraws consent, or dies.

For subjects who are enrolled, but do not receive axicabtagene ciloleucel infusion, any additional antilymphoma therapy will also be collected until subject completes the long-term follow-up period, lost to follow-up, withdraws consent, or dies.

5.1.8. Toxicity Management

To date, the following risks have been identified with axicabtagene ciloleucel: CRS, neurologic events, infections, cytopenias, and hypogammaglobulinemia. As the safety experience with axicabtagene ciloleucel increases, the management guidance may be updated.

6. STUDY PROCEDURES

The visit schedule is calculated from axicabtagene ciloleucel infusion on Day 0.

The visit schedule for disease assessments is also calculated from axicabtagene ciloleucel infusion on Day 0, including CT scans, PET scans, bone marrow biopsy, physical exams needed to assess disease, and collection of subsequent antilymphoma therapy.

An overview of study assessments/procedures is outlined below. Refer to the CRF completion guidelines for data collection requirements and best practices for documentation of study procedures.

6.1. Informed Consent

Before a subject participates in the clinical study, the investigator is responsible for obtaining written informed consent from the subject after adequately explaining the study design, anticipated benefits, and potential risks. Subjects should sign the most current institutional Review Board/Independent Ethics Committee (IRB/IEC)-approved ICF before any nonstandard-of-care study-specific activity or procedure is performed.

The consent process and the subject's agreement or refusal to participate in the study must be documented in the subject's medical records. If the subject agrees to participate, the ICF must be signed and dated by both the subject and the person who conducted the informed consent discussion. The original signed ICF will be retained in accordance with institution policy and IRB/IEC requirements, and a copy of the ICF will be provided to the subject.

All subjects who are enrolled into the study should be reconsented with any updated version of the IRB/IEC-approved ICF if the new version is relevant to their participation.

6.2. Screening

Investigative sites will maintain a log of all screened subjects who were reviewed and evaluated for study participation. Information collected in the screening log should include limited information, such as the date of screening, date the subject was enrolled, or the reason for why the subject failed screening.

The screening period begins on the date the subject signs the IRB/IEC-approved ICF and continues through confirmation of eligibility into the study. Informed consent must be obtained before completion of any nonstandard-of-care study-specific procedures. Procedures that are part of standard of care are not considered study-specific and, therefore, may be performed prior to obtaining consent and used to confirm eligibility provided they occur within the time allowance outlined below.

After written informed consent has been obtained, Kite will assign a unique subject ID number to the subject.

See Section 6.2.1 for the study procedures for subjects who rescreen into the study. Only subjects who meet the eligibility criteria listed in Section 4 will be enrolled into the study. If at any time prior to enrollment the subject fails to meet the eligibility criteria, the subject should be designated as a screen failure within the subject's screening log, and the reasons for failing screening should also be recorded.

6.2.1. Rescreening

Subjects who are unable to complete or meet the eligibility criteria during the 28-day screening period will be permitted to rescreen 1 time. Subjects will retain the same subject ID number assigned at the original screening. If rescreening occurs within 28 days of the signing of the original informed consent, it is only necessary to perform the procedure(s)/assessment(s) that did not originally meet the eligibility criteria; all other initial screening procedures/assessments do not need to be repeated. If rescreening occurs after more than 28 days, or leukapheresis is delayed more than 28 days from the signing of the original informed consent, subjects must be reconsented and repeat all screening procedures/assessments.

6.3. Demographic Data

Demographic data will be collected as per country and local regulations and guidelines. Where applicable, demographic data will include sex, year of birth, race, ethnicity, and country of enrollment to study a possible association between these variables and subject safety and treatment effectiveness.

6.4. Medical and Treatment History

Relevant medical history prior to the start of adverse event reporting will be collected. Relevant medical history is defined as data on the subject's current medical condition that would be typically shared in a referral letter. In addition to the medical history, all history related to the subject's disease, treatment, and response to treatment will be collected and must date back to the original diagnosis. All findings will be recorded on the CRFs.

For subjects who are being referred from another clinic or institution to the participating research center, copies from the subject's chart should be obtained.

6.5. Conditioning Chemotherapy and Axicabtagene Ciloleucel Infusion

Administration of CAR T cells to subjects with ongoing infection or inflammation, even if such processes are asymptomatic, increases the risk of high-grade and fatal toxicity. All efforts should be made to rule out such conditions prior to cell infusion. Signs, symptoms, or abnormal laboratory results attributed to the malignancy tumor fever and elevated C-reactive protein (CRP) are diagnoses of exclusion that require a documented workup to establish. Conditioning chemotherapy and axicabtagene ciloleucel infusion should be initiated only once it is reasonably assured that cell infusion can safely proceed.

Refer to Section 6.5.3 for requirements for workup of potential infectious and/or inflammatory states.

6.5.1. Requirements for Initiating Conditioning Chemotherapy

If any of the following criteria are met prior to the initiation of conditioning chemotherapy, then the workup listed in Section 6.5.3 must be performed to determine the potential cause if there is no identified source of infection.

- Temperature > 38°C within 72 hours of conditioning chemotherapy
- CRP > 100 mg/L anytime between enrollment to start of conditioning chemotherapy
- White blood cell (WBC) count or WBC differential concerning for infectious process between enrollment to start of conditioning chemotherapy WBC > 20,000, rapidly increasing WBC, or differential with high percentage of segs/bands)

Additionally:

- If any screening assessments or procedures are repeated between confirmation of eligibility and the start of conditioning chemotherapy and results are outside the eligibility criteria listed in Section 4, then the condition must resolve prior to proceeding with conditioning chemotherapy.
- Complete history and physical exam including head, eyes, ears, nose, and throat (HEENT), and cardiac, vascular, respiratory, gastrointestinal, integumentary, and neurological systems must not reveal evidence of infection/inflammation.
- The subject must not have received systemic antimicrobials for the treatment of a known or suspected infection within 48 hours before conditioning chemotherapy (prophylactic use of antimicrobials is allowed).
- The treatment course of any antimicrobials given for known or suspected antecedent infection should be complete as per infectious disease consult (if applicable) recommendation before stopping or switching to prophylactic antimicrobials.
- If a subject is confirmed to have an infectious process for which antimicrobials are not available (eg, viral pneumonia), the infection must be clinically resolved as determined by the investigator in consultation with the infectious disease service (if applicable).
- Most recently collected blood, urine, or other body fluid cultures must show no growth for at least 48 hours, and any other infectious workup performed (bacterial, viral serologies, polymerase chain reaction (PCR), stool studies, imaging studies) must be negative. If clinical suspicion is for an infection for which cultures are unlikely to be positive within 48 hours (eg, fungal infection), adequate time must be allowed for cultures to become positive.

Once the above criteria are met, then the subject can proceed with conditioning chemotherapy.

6.5.2. Requirements for Initiating Axicabtagene Ciloleucel Infusion

If any of the following criteria are met prior to the initiation of axicabtagene ciloleucel infusion, then the workup listed in Section 6.5.3 must be performed to determine the potential cause if there is no identified source of infection.

- Temperature > 38°C within 72 hours of axicabtagene ciloleucel infusion
- CRP > 100 mg/L anytime between enrollment to start of axicabtagene ciloleucel infusion
- WBC count or WBC differential concerning for infectious process between enrollment to start of axicabtagene ciloleucel infusion WBC > 20,000, rapidly increasing WBC, or differential with high percentage of segs/bands)

Additionally:

- If any screening assessments or procedures are repeated between confirmation of eligibility and the start of axicabtagene ciloleucel infusion and results are outside the eligibility criteria listed in Section 4, then the condition must resolve prior to proceeding with axicabtagene ciloleucel infusion (except for peripheral blood cell counts that have been impacted by conditioning chemotherapy).
- Complete history and physical exam including HEENT, and cardiac, vascular, respiratory, gastrointestinal, integumentary, and neurological systems must not reveal evidence of infection/inflammation.
- The subject must not have received systemic antimicrobials for the treatment of a known or suspected infection within 48 hours before axicabtagene ciloleucel (prophylactic use of antimicrobials is allowed).
- The treatment course of any antimicrobials given for known or suspected antecedent infection should be complete as per infectious disease consult (if applicable) recommendation before stopping or switching to prophylactic antimicrobials.
- If a subject is confirmed to have an infectious process for which antimicrobials are not available (eg, viral pneumonia), the infection must be clinically resolved as determined by the investigator in consultation with the infectious disease service (if applicable).
- Most recently collected blood, urine, or other body fluid cultures must show no growth for at least 48 hours, and any other infectious workup performed (bacterial, viral serologies, PCR, stool studies, imaging studies) must be negative. If clinical suspicion is for an infection for which cultures are unlikely to be positive within 48 hours (eg, fungal infection), adequate time must be allowed for cultures to become positive.

Once the above criteria are met, then the subject can proceed with administration of axicabtagene ciloleucel.

If the axicabtagene ciloleucel infusion is delayed > 2 weeks, protocol-specified conditioning chemotherapy must be repeated.

6.5.3. Requirements to Work-up Potential Infectious and/or Inflammatory States

In the absence of an identified source of infection (such as line infection, pneumonia on chest x-ray), the minimum workup to be performed prior to administration of conditioning chemotherapy and/or axicabtagene ciloleucel consists of:

- Call Kite medical monitor
- Infectious disease service consult
- CT imaging of the chest, abdomen, and pelvis with IV contrast. If there is a medical contraindication to contrast, then non-contrast CT is allowed.
- The following must be performed (prior to the initiation of antimicrobials if clinically feasible):
  - Blood cultures (aerobic and anaerobic x 2 bottles each) and urinalysis and urine culture. Deep/induced sputum culture if clinically indicated
  - All indwelling lines, such as central venous catheters, should be examined for any signs of infection and additional cultures should be drawn from the line.
  - Nasopharyngeal-throat swab or equivalent assay for viral infection, such as influenza A/B (including H1N1), parainfluenza 1/2/3, adenovirus, respiratory syncytial virus, coronavirus, metapneumovirus
  - Collection of fungal cultures and markers as appropriate (galactomannan, Fungitell®)
  - Collection of appropriate serum viral studies (eg, cytomegalovirus)
- If a CNS process is suspected, appropriate brain imaging and subsequent lumbar puncture with cytology, culture, Gram stain, and viral PCR should be performed.
- Any additional sign or symptom-directed investigation should be performed as clinically indicated.

Prior to proceeding with conditioning chemotherapy or axicabtagene ciloleucel infusion, the above workup must not suggest the presence of an active infection and all requirements for conditioning chemotherapy and axicabtagene ciloleucel infusion must be satisfied.

If the above workup was triggered due to CRP > 100 mg/L, testing for CRP should be repeated, and if CRP continues to increase significantly, evaluation should be performed for any other potential infectious or inflammatory condition not previously evaluated.

6.5.4. Conditioning Chemotherapy Administration (Day –5 Through Day –3 Prior to Axicabtagene Ciloleucel Infusion)

Subjects will receive a conditioning chemotherapy regimen consisting of cyclophosphamide and fludarabine. The first dose of conditioning chemotherapy will be designated as Day –5. Subjects will initiate conditioning chemotherapy with cyclophosphamide and fludarabine beginning on Day –5 and through Day –3, with 2 rest days (Day –2 and Day –1) before receiving axicabtagene ciloleucel. The 3-day conditioning chemotherapy regimen will be administered in an outpatient setting.

The 3-day conditioning regimen of fludarabine and cyclophosphamide will be administered in accordance with the following daily dosing instructions:

- IV hydration with a balanced crystalloid according to institutional guidelines prior to administration of cyclophosphamide on the day of infusion
- Cyclophosphamide 500 mg/m^2 IV over approximately 60 minutes or per institutional guidelines
- Fludarabine 30 mg/m^2 IV over approximately 30 minutes or per institutional guidelines
- Additional IV hydration with a balanced crystalloid according to institutional guidelines to be administered upon completion of the cyclophosphamide infusion
- Mesna to be administered per institutional guidelines

Subjects should be instructed to drink plenty of liquids during chemotherapy and throughout the 24-hour period following chemotherapy (approximately 2 L/24 hours). In general, subjects should be kept well hydrated but closely monitored to prevent fluid overload.

6.6. Physical Exam, Vital Signs, and Performance Status

All physical exam changes noted in subsequent exams when compared to the baseline exam will be reported as AEs.

Vital signs, including blood pressure (BP), heart rate, respiratory rate, oxygen saturation, and temperature, will be monitored and recorded. It is recommended that vital signs are monitored during and after study treatment and as clinically indicated.

Performance status as measured by the ECOG scale will be performed to quantify the subject’s general well-being and ability to perform activities of daily life.

6.7. Neurological Examination

A neurological examination will be performed, and any of the following abnormalities will be recorded: level of consciousness, orientation, vision, cranial nerves and brain stem functions, pyramidal and extra pyramidal motor system, reflexes, muscle tone and trophic findings,

coordination, sensory system, and neuropsychological findings (eg, speech, cognition, and emotion).

A neurological examination should be done prior to axicabtagene ciloleucel infusion on treatment Day 0, then on Day 1, Day 3, Day 5, and Day 7 during the observation period, which must last a minimum of 7 days.

For new onset of neurologic symptoms suspected to be related to axicabtagene ciloleucel treatment, refer to Section 5.1.8. Subjects will be specifically asked about changes in neurological status since the previous neurological examination.

6.8. Disease Assessment

Subjects will be evaluated for disease response by the site investigator. Disease assessments will be evaluated per the Lugano Classification {Cheson 2014} (see Appendix 1). Flow cytometric, molecular, or cytogenetic studies will not be used to determine response.

6.8.1. Imaging

6.8.1.1. Pre-treatment Disease Assessment

A fluorodeoxyglucose (FDG)-PET from skull base to mid-thighs and a diagnostic quality contrast-enhanced (unless contraindicated) CT from skull base through lesser trochanters (PET-CT), along with the appropriate imaging of all other sites of disease must be performed within 28 days prior to enrollment/leukapheresis.

If a PET-CT cannot be performed as standard of care following 2 cycles of chemoimmunotherapy, a subject may have the PET-CT performed on study after informed consent is obtained.

If the PET-CT performed following 2 cycles of chemoimmunotherapy is > 28 days from enrollment/leukapheresis, or if subject receives bridging therapy between screening and conditioning chemotherapy, the PET-CT must be repeated to confirm subject remains PET2+.

6.8.1.2. Post-treatment Response Assessment

Post-treatment PET-CT response assessments will begin at Day 28 and continue at time points. If there was evidence of baseline bone marrow involvement and no PET-CT is available or if there are unexplained cytopenias or suspicion of bone marrow involvement, then a bone marrow aspirate and biopsy will be performed in subjects who are being assessed for CR. To confirm a CR, the bone marrow aspirate and biopsy must show no evidence of disease by morphology, or, if indeterminate by morphology, must be negative by immunohistochemistry.

PET-CTs will continue through Month 24 or until disease progression, whichever comes first. If the subject's disease has not progressed by Month 24, disease assessments can then be performed at standard-of-care intervals. Subjects with symptoms suggestive of disease progression should be evaluated for progression at the time symptoms occur even if this requires an unscheduled

visit. If the subject has started subsequent antilymphoma therapy, then imaging assessments will no longer be required per protocol. PET-CT can be performed at any time disease progression is suspected. FDG-PET assessment takes precedence over CT assessment for time points when both are available. If only CT is available for a particular time point, then the CT assessment should include a comparison with and may be affected by the PET-CT assessment at the prior time point. Please refer to the imaging manual for further details.

Bone marrow aspirate/biopsy should also be considered to evaluate hemophagocytic lymphohistiocytosis as indicated.

6.8.1.3. Response Evaluation to Retreatment

For the purpose of determining response to retreatment with axicabtagene ciloleucel, the last scan prior to retreatment will be considered the baseline.

6.8.2. Determination of Bone Marrow Involvement

A subject's bone marrow involvement should be confirmed by PET-CT or bone marrow biopsy and aspirate prior to enrollment. Confirmation of marrow involvement from determination at initial diagnosis or between diagnosis and screening is acceptable.

6.9. Cell Collection and Axicabtagene Ciloleucel Study Treatment Schedule and Administration

6.9.1. Leukapheresis

Subjects must remain eligible per the eligibility criteria outlined in Section 4 prior to the start of leukapheresis.

If any screening assessments or procedures are repeated between confirmation of eligibility and the start of leukapheresis and results are outside the eligibility criteria listed in Section 4, contact the sponsor's medical monitor prior to proceeding with leukapheresis.

Before leukapheresis commences, the following criteria must be met:

- No evidence or suspicion of an infection
- Corticosteroid therapy at a pharmacologic dose (≥ 5 mg/day of prednisone or equivalent doses of other corticosteroids) and other immunosuppressive drugs must be avoided for 7 days prior to leukapheresis

The leukapheresis visit should occur within approximately 5 days of eligibility confirmation. After a subject commences leukapheresis, the subject will be considered enrolled into the study.

If criteria are not met, leukapheresis must be delayed until the event resolves. If leukapheresis is delayed more than 5 days after eligibility confirmation, baseline CBC with differential and

chemistry panel must be repeated. If results are outside the eligibility criteria listed in Section 4, contact the medical monitor prior to proceeding with leukapheresis.

After the above criteria are met, mononuclear cells will be obtained by leukapheresis. The leukapheresed cells are then packaged for expedited shipment to the manufacturing facility.

6.9.2. Non-Chemotherapy Bridging Therapy

If prescribed, bridging therapy must be administered after leukapheresis/enrollment and must be completed prior to initiating conditioning chemotherapy per the specifications outlined in Section 5 for bridging therapy.

6.9.3. Axicabtagene Ciloleucel Treatment Period

6.9.3.1. Axicabtagene Ciloleucel Premedication Dosing

The following pre-axicabtagene ciloleucel infusion medications should be administered approximately 1 hour prior to infusion:

- Acetaminophen 500 to 1000 mg taken orally or equivalent
- Diphenhydramine 12.5 to 25 mg administered either orally or via IV or equivalent

6.9.3.2. Axicabtagene Ciloleucel Administration Day 0

All subjects will receive axicabtagene ciloleucel infusion at a healthcare facility, followed by daily monitoring at a healthcare facility for ≥ 7 days to monitor for signs and symptoms of CRS and neurologic events. Alternatively, subjects may be hospitalized to receive their axicabtagene ciloleucel infusion and be observed for CRS and neurologic events in the hospital setting, if deemed appropriate by the investigator. Post-infusion monitoring of subjects must be for a minimum of 7 days unless otherwise required by country regulatory agencies (refer to Appendix 3).

If subjects are hospitalized, subjects should not be discharged from the hospital until all axicabtagene ciloleucel-related non-hematological toxicities resolve to Grade 1 or lower, or return to baseline. Subjects may be discharged with non-critical and clinically stable or improving toxicities (eg, renal insufficiency) even if higher than Grade 1, if deemed appropriate by the investigator. Subjects should remain in a hospital for ongoing axicabtagene ciloleucel-related fever, hypotension, hypoxia, or ongoing neurologic events higher than Grade 1, or if deemed necessary by the investigator.

Subjects should be instructed to remain within proximity of the clinical study site for at least 4 weeks following axicabtagene ciloleucel infusion. Subjects should be advised to refrain from driving and engaging in hazardous occupations or activities, such as or operating heavy or potentially dangerous machinery, for at least 8 weeks following axicabtagene ciloleucel infusion. Subjects and their family members/caregivers should be educated on potential CRS and neurologic symptoms, such as fever, dyspnea, confusion, aphasia, dysphasia, somnolence, encephalopathy, ataxia, or tremor. Subjects or their family members/caregivers should be

instructed to immediately contact the treating investigator or seek immediate medical attention if any of these symptoms develop.

Central venous access, such as a port or a peripherally inserted central catheter, is required for the administration of axicabtagene ciloleucel. Catheter care, per institutional guidelines, should be followed. . Vital signs should be measured during and after axicabtagene ciloleucel treatment (see Section 6.6).

Research sites should follow institutional guidelines for the infusion of cell products.

6.9.3.3. Axicabtagene Ciloleucel Retreatment

Subjects who achieve a PR or CR and subsequently experience disease progression may have an option to receive a second course of conditioning chemotherapy and axicabtagene ciloleucel.

The following criteria must be met prior to being considered for a repeat course of therapy:

- Subject had a PR or CR at any time after axicabtagene ciloleucel therapy and then subsequently progressed with CD19 tumor expression confirmed centrally by biopsy after disease progression and prior to retreatment
- Subject continues to meet the original study eligibility criteria with the exception of prior axicabtagene ciloleucel use in this study; screening assessments and procedures should be repeated if clinically indicated (eg, ECHO)
- Subject has not received subsequent therapy for the treatment of lymphoma
- Subject did not experience a life-threatening toxicity related to axicabtagene ciloleucel during the original course of treatment
- Toxicities related to conditioning chemotherapy (fludarabine and cyclophosphamide), with the exception of alopecia, have resolved to Grade 1 or lower, or returned to baseline prior to retreatment

Sites are required to collect a biopsy confirming disease progression and CD19 expression and to submit the biopsied tissue to the central laboratory before initiating retreatment.

The decision to retreat should be made in consultation with the sponsor's medical monitor. In addition, before performing any study-related procedures or treatment, it is necessary to 1) discuss the risks and benefits of retreatment with the subject, and 2) confirm with the subject how the second dose will be manufactured. The second dose could be manufactured at the same time that the first axicabtagene ciloleucel dose is made with existing PBMCs. Alternatively, the subject may need to undergo a second leukapheresis and should be informed of this possibility. These conversations should also be recorded in the subject's source document.

A maximum of 1 retreatment course may occur per subject. Subjects who are retreated will follow the same treatment schedule and procedural requirements per the initial treatment.

After a subject is deemed eligible for retreatment and the means by which the second dose of axicabtagene ciloleucel has been confirmed (which will include determining whether a second leukapheresis is required), the subject will follow the study procedure requirements of the current IRB-approved protocol.

6.9.3.4. Central Confirmation of Diagnosis

Local diagnosis of large B-cell lymphoma for determination of study eligibility will be made by the investigator at each site. Diagnosis will be confirmed retrospectively by a centralized specialty laboratory.

6.9.3.5. Pharmacokinetics and Pharmacodynamics

Pharmacokinetic (PK) and pharmacodynamic analyses will be performed on blood (levels of anti-CD19 CAR T cells) and serum (cytokines) to evaluate predictive markers for the efficacy and safety of axicabtagene ciloleucel.

6.9.3.6. Product Characteristics

Samples of leukapheresis material or final product will be retained and tested by the sponsor or specialty laboratory.

6.10. Post-treatment Assessment Period

After completing study treatment, all subjects will return to the clinic for post-treatment follow-up visits.

If a subject does not respond to treatment at any time during the post-treatment assessment period, then the subject will continue to undergo the post-treatment follow-up and then be followed for survival and disease outcomes in the long-term follow-up portion of the study starting with the Month 6 visit.

6.11. Long-term Follow-up Period

All enrolled subjects will be followed in the long-term follow-up period for safety analysis and survival and disease status for up to 15 years, if applicable. Subjects will begin the long-term follow-up period beginning at the Month 6 visit. Subjects who did not respond to treatment may receive off-protocol therapy but will continue to be followed for disease assessments (if progression was not documented), subsequent antilymphoma therapy, and survival.

Subjects may also be contacted by telephone to confirm survival status and subsequent antilymphoma therapy use. If the subject fails to return to the clinic for a scheduled protocol-specific visit, sites will need to make 2 attempts, using both the telephone and either mail or email to contact the subject. Sites must document both attempts to contact the subject. If a subject does not respond within 1 month after the second contact, then the subject will be considered lost to follow-up, and no additional contact will be required. Subjects who are

enrolled, but either did not receive the investigational product or received therapy but did not respond to treatment, will be followed in the long-term follow-up period.

7. SUBJECT WITHDRAWAL

Subjects have the right to withdraw from the study at any time and for any reason without prejudice to their future medical care by the physician or at the institution.

7.1. Reasons for Removal from Treatment

Reasons for removal from protocol-required investigational products or procedures include any of the following:

- AEs
- Subject request
- Product not available
- Lost to follow-up
- Death
- Decision by sponsor

7.2. Reasons for Removal from Study

Reasons for removal of a subject from the study are as follows:

- Subject withdrawal of consent from further follow-up
- Investigator decision
- Lost to follow-up
- Death

8. SAFETY REPORTING

8.1. Adverse Events

An AE is defined as any untoward medical occurrence in a clinical trial subject. The event does not necessarily have a relationship with study treatment. The investigator is responsible for ensuring that any AEs observed by the investigator or reported by the subject are recorded in the subject's medical record. The definition of AEs includes worsening of a pre-existing medical condition. When recording such events, provide descriptions that the pre-existing condition has changed.

A pre-existing condition that has not worsened during the study or involves an intervention, such as elective cosmetic surgery or a medical procedure while on study, is not considered an AE.

Interventions for pretreatment conditions (such as elective cosmetic surgery) or medical procedures that were planned before study participation are not considered AEs.

The term "disease progression," as assessed by measurement of malignant lesions on radiographs or other methods, should not be reported as AEs. Death due to disease progression in the absence of signs and symptoms should be reported under the primary tumor type (eg, B-cell lymphoma).

When an AE or SAE is due to the disease under investigation, it is necessary to report the signs and symptoms. Worsening of signs and symptoms of the malignancy under study should also be reported as AEs in the appropriate section of the CRF.

The investigator's clinical judgment is used to determine whether a subject is to be removed from treatment due to an AE. If a subject requests to withdraw from protocol-required therapies or the study because of an AE, then the subject should undergo the procedures outlined in the Month 3 visit.

8.2. Reporting of Adverse Events

The investigator is responsible for reporting all AEs observed by the investigator or reported by the subject that occur from enrollment (ie, commencement of leukapheresis) through 3 months after treatment with axicabtagene ciloleucel infusion.

After 3 months, targeted AEs (eg, infections, neurological, hematological, and autoimmune disorders, and secondary malignancies) will be monitored and reported for 24 months after treatment with axicabtagene ciloleucel or until disease progression, whichever occurs first.

For subjects who are enrolled, but do not receive axicabtagene ciloleucel, the AE reporting period ends 30 days after the last study-specific procedure (eg, leukapheresis, conditioning chemotherapy).

The investigator must provide the information listed below regarding the AEs being reported:

- AE diagnosis or syndrome (if not known, signs or symptoms)

- Dates of onset and resolution
- Severity
- Assessment of relatedness to axicabtagene ciloleucel, conditioning chemotherapy, or study procedures
- Action taken

The AE grading scale used will be the National Cancer Institute (NCI) Common Terminology Criteria for Adverse Events (CTCAE) version 5.0. A copy of the grading scale can be downloaded from the Cancer Therapy Evaluation Program home page (http://ctep.cancer.gov).

In reviewing AEs, investigators must assess whether the AE is possibly related to 1) axicabtagene ciloleucel, 2) conditioning chemotherapy, 3) any protocol-required study procedure or treatment, 4) disease progression, 5) concurrent disease, 6) concomitant medication, or 7) other. The relationship is indicated by a Yes or No response and entered into the CRF. A Yes response should indicate that there is evidence to suggest a causal relationship between the study treatment or procedure and the AE. Additional relevant data with respect to describing the AE will be collected in the CRFs.

The investigator is expected to follow reported AEs until stabilization or resolution. If a subject begins a new antilymphoma therapy, the AE reporting period for non-SAEs ends at the time the new treatment is started.

8.3. Definition of Serious Adverse Events

An SAE is defined as an AE that meets at least 1 of the following serious criteria:

- Fatal
- Life-threatening (places the subject at immediate risk of death)
- Requires inpatient hospitalization or prolongation of existing hospitalization
- Results in persistent or significant disability/incapacity
- Congenital anomaly/birth defect
- Other medically important event

An AE would meet the criterion of “requires hospitalization” if the event necessitated an admission to a healthcare facility (eg, overnight stay).

Events that require an escalation of care when the subject is already hospitalized should be recorded as an SAE. Examples of such events include movement from routine care in the

hospital to the intensive care unit or if that event resulted in a prolongation of the existing planned hospitalization.

If an investigator considers an event to be clinically important, but it does not meet any of the serious criteria, the event could be classified as a serious adverse event with the criterion of "other medically important serious event."

The terms "severe" and "serious" are not synonymous. Severity refers to the intensity of an AE according to NCI CTCAE criteria; the event itself may be of relatively minor medical significance and, therefore, may not meet the seriousness criteria. Severity and seriousness need to be independently assessed for each AE recorded on the electronic CRF (eCRF).

8.4. Reporting of Serious Adverse Events

The investigator is responsible for reporting all SAEs observed by the investigator or reported by the subject that occur after signing of the informed consent through 3 months after the axicabtagene ciloleucel infusion or until the initiation of another antilymphoma therapy, whichever occurs first. After 3 months, only targeted SAEs will be reported. Targeted SAEs are defined as and include infections; neurological, hematological, and autoimmune disorders; and secondary malignancies that occur up to 24 months after axicabtagene ciloleucel infusion or until disease progression, whichever occurs first.

SAEs, which the investigator assesses as related to axicabtagene ciloleucel, should be reported regardless of the time period.

For subjects who screen fail or are enrolled, but do not receive axicabtagene ciloleucel, the reporting period for SAEs ends 30 days after the last study-specific procedure (eg, screening procedure, leukapheresis, conditioning chemotherapy).

All SAEs must be submitted to Kite via the electronic SAE system within 24 hours of the investigator's knowledge of the event. If the electronic SAE system is unavailable (eg, system outage), then the SAE must be submitted using the SAE Report Form and emailed to the SAE Reporting mailbox: safety_fc@gilead.com.

Subsequently, all SAEs will be reported to the health authorities per local reporting guidelines.

Disease progression of the malignancy is not considered an AE. However, signs and symptoms of disease progression may be recorded on the CRF as AEs or SAEs and indicated as being due to disease progression. If the malignancy has a fatal outcome before the end of the SAE reporting period, then the event leading to the death must be recorded as an SAE with the outcome being fatal.

Death must be reported if it occurs during the SAE reporting period, irrespective of any intervening treatment.

Any death occurring after the first dose of chemotherapy, for the purpose of pre-conditioning, and within 3 months of axicabtagene ciloleucel infusion, regardless of attribution to treatment,

requires expedited reporting within 24 hours. Any death occurring after the SAE reporting period requires expedited reporting within 24 hours only if it is considered related to treatment.

8.5. Reporting Deaths

Deaths that occur during the protocol-specified AE reporting period that are attributed by the investigator solely to progression of underlying lymphoma should be recorded as SAEs with the preferred term "B-cell lymphoma" and must be reported immediately to the sponsor. Death is an outcome and not a distinct event. The event or condition that caused or contributed to the fatal outcome should be recorded on the AE form. However, every effort should be made to capture the established cause of death, which may become available later on (eg, after autopsy). Refer to the CRF completion guidelines for detailed instructions.

8.6. Diagnosis Versus Signs and Symptoms

For AEs, a diagnosis (if known) rather than individual signs and symptoms should be recorded on the AE form. The exception is for CRS where both the diagnosis and the signs and symptoms will be captured on the AE form. For signs and symptoms of the underlying cancer, the signs and symptoms should be captured. However, on the AE form, the investigator should state that these signs and symptoms are due to the underlying disease.

8.7. Pregnancy and Lactation

There is no relevant clinical experience with axicabtagene ciloleucel in pregnant or lactating women, and animal reproductive studies have not been performed. Women of childbearing potential must have a negative pregnancy test prior to enrollment because of the potentially dangerous effects of the preparative chemotherapy on the fetus. Women of childbearing potential should be monitored according to local and country-specific regulations. This experimental therapy should not be administered to pregnant women or women who are breastfeeding.

Female subjects and female partners of male subjects are recommended to use highly effective contraception (method must achieve an annual failure rate of < 1%) for at least 6 months after conditioning chemotherapy dosing or the administration of axicabtagene ciloleucel, whichever is longer. Male subjects are recommended to not father a child for at least 6 months after the conditioning chemotherapy dosing or the administration of axicabtagene ciloleucel, whichever is longer. Refer to Appendix 4 for a complete list of highly effective contraception methods.

If a pregnancy occurs in either a female subject enrolled into the study or a female partner of a male subject within 6 months of completing conditioning chemotherapy or the administration of axicabtagene ciloleucel, whichever is longer, the pregnancy must be reported to the sponsor. Information regarding the pregnancy and/or the outcome may be requested by the sponsor.

The pregnancy should be reported to the sponsor within 24 hours of the investigator's knowledge of the pregnancy event by using the pregnancy report form and emailing it to: safety_fc@gilead.com.

The pregnancy outcome should be reported to the sponsor using the pregnancy outcome report form and emailing it to: safety_fc@gilead.com.

Lactation cases occurring in female patients taking protocol-required therapies (PRT) and up to 6 months after PRT should be reported to safety_fc@gilead.com within 24 hours of awareness using the lactation reporting form.

8.8. Hospitalization and Prolonged Hospitalization

Any AE that results in hospitalization or prolonged hospitalization should be documented and reported as an SAE as described in Section 8.4.

The following hospitalization scenarios are not considered to be SAEs:

- Hospitalization for palliative care or hospice care
- Planned hospitalization required by the protocol (eg, for monitoring of the subject or to perform an efficacy measurement for the study)
- Planned hospitalization for a pre-existing condition
- Hospitalization due to progression of the underlying cancer

8.9. Abnormal Vital Sign Values

Not all vital sign abnormalities qualify as an AE. A vital sign result must be reported as an AE if it is a change from baseline and meets any of the following criteria:

- Accompanied by clinical symptoms
- Results in a medical intervention or a change in concomitant therapy
- Clinically significant in the investigator’s judgment

It is the investigator’s responsibility to review all vital sign findings. Medical and scientific judgment should be exercised in deciding if an isolated vital sign abnormality should be classified as an AE. However, if a clinically significant vital sign abnormality is a sign of a disease or syndrome (eg, high BP), only the diagnosis (ie, hypertension) should be recorded on the CRF.

8.10. Data Safety Monitoring Board

An independent Data Safety Monitoring Board (DSMB) will be chartered to meet and review the serious adverse events (SAEs) and suspected unexpected serious adverse reactions (SUSARs) on a semi-annual basis after the first subject has been treated with axicabtagene ciloleucel up through the primary analysis. Kite Pharma, Inc., or delegate, will submit SAEs and SUSARs to the DSMB on a regular basis throughout the study up through the primary analysis. The DSMB

will also meet to review safety data after 15 subjects have been enrolled and treated with axicabtagene ciloleucel and have had an opportunity to be followed for 3 months after axicabtagene ciloleucel infusion. The DSMB will make trial conduct recommendations on an ongoing basis based on an analysis of risk vs benefit. The DSMB may request additional safety data for review or recommend modifications to the study conduct if safety concerns are identified. The DSMB may meet more often as needed.

The sponsor may request additional reviews by the DSMB. Data submitted to the DSMB may be monitored or unmonitored to facilitate timely DSMB review. At the time of expedited reporting of SUSARs to the FDA, Kite Pharma (or designee) will concurrently submit these reports to the DSMB chair.

9. STATISTICAL CONSIDERATIONS

9.1. Hypothesis

No formal hypothesis will be tested. This study is designed to estimate the CR rate in subjects with high-risk large B-cell lymphoma. The CR rate targeted in this study is 60%.

9.1.1. Covariates and Subgroups

The following covariates at screening/baseline may be used in efficacy and safety analyses:

- Age (< 65, ≥ 65 years)
- Gender
- Race
- Ethnicity
- ECOG status
- IPI score
- Diagnosis category (double-hit lymphomas versus triple-hit lymphomas versus non-double-/triple-hit with IPI score ≥ 3)
- Levels of cytokines
- Levels of CAR T cells
- CR achieved on the study

Additional associative analyses of covariates with subject outcomes will be specified in the statistical analysis plan.

9.2. Sample Size Considerations

In the GELA randomized Phase 2 study evaluating the efficacy of R-ACVBP or R-CHOP-14 induction, using IWF 2007 criteria in young patients with high-risk DLBCL, the primary objective of achieving a higher than 50% CR rate after 4 cycles of induction regimen was not met in both randomization groups (47% in R-ACVBP and 39% in R-CHOP-14) {Casasnovas 2017}. Accordingly, it is postulated that a CR rate of 60% would represent a clinically meaningful improvement over standard chemoimmunotherapy in a high-risk large B-cell lymphoma patient population. Although there is no formal hypothesis testing, the sample size has been determined in part with descriptive analysis described below.

The trial uses a single-arm design to estimate the CR rate in subjects with high-risk large B-cell lymphoma treated with axicabtagene ciloleucel. A CR rate of 60% with axicabtagene ciloleucel treatment is targeted. With a total sample size of 40 subjects, an observed CR rate of 60% will yield an 80% CI for the response rate with a maximum half-width of less than or equal to 11%, corresponding to a lower limit of at least 48.6%. This target CR rate, and the lower limit of the 80% CI for the CR rate, is meaningful because it would represent a significant improvement in the response rate for the subjects with high-risk large B-cell lymphoma and would likely offer an improvement over existing therapies in patients with high -risk large B-cell lymphoma.

Table 1 provides the estimated CR rate and the lower and upper limits of 80%/95% CI based on the Clopper-Pearson method for a range of possible CR rates for a sample of 40 subjects.

Table 1. Lower and Upper Limits of 80%/95% CIs for CR Rates from 60% to 100% for a Sample of 40 Subjects

Number (%) of CRs	24 (60)	28 (70)	32 (80)	36 (90)	40 (100)
Lower and upper limits of 80% CI (%, %)	48.6, 70.6	58.8, 79.5	69.6, 88.0	81.0, 95.6	94.4, 100
Lower and upper limits of 95% CI (%, %)	43.3, 75.1	53.5, 83.4	64.4, 90.9	76.3, 97.2	91.2, 100

Abbreviations: CI, confidence interval; CR, complete response.

9.3. Access to Individual Subject Treatment Assignments

This is a single-arm, open-label study, and subjects and investigators will be aware of treatment received. Data-handling procedures for the study will be devised to reduce potential sources of bias and maintain the validity and credibility of the study. These procedures will be outlined in the study statistical plan, DSMB charter, and trial integrity document.

9.4. Interim Analysis and Early Stopping Guidelines

The DSMB will meet and review SAEs and SUSARs on a semi-annual basis after the first subject has been dosed. The DSMB may request additional safety data.

One planned interim analysis will be performed. Interim Analysis 1 will be conducted after 15 subjects have been enrolled and treated with axicabtagene ciloleucel and have had an opportunity to be followed for 3 months after axicabtagene ciloleucel infusion. This analysis will be for efficacy and safety and will be descriptive.

9.5. Analysis Subsets

In this study, subjects are to be dosed at a target of 2×10^6 (1.0×10^6 to 2.4×10^6) anti-CD19 CAR T cells/kg. A minimum dose of 1×10^6 anti-CD19 CAR T cells/kg may be administered. For subjects weighing $\geq$ 100 kg, a maximum flat dose of 2×10^8 anti-CD19 CAR T cells will be administered. Subjects are considered to have received the target dose if they receive at least 1×10^6 anti-CD19 CAR T cells/kg.

The safety analysis set is defined as all subjects treated with any dose of axicabtagene ciloleucel. This analysis set will be used for all safety data analyses.

The full analysis set will consist of all enrolled/leukapheresed subjects and will be used for the summary of subject disposition and the listing of deaths.

The response evaluable analysis set will consist of the subjects who are enrolled and treated with axicabtagene ciloleucel at a dose of at least 1 x 10^6 anti-CD19 CAR T cells/kg and have a centrally confirmed disease type (double-/triple- hit lymphomas) or large B--cell lymphoma with an IPI score ≥ 3. This analysis set will be used for all efficacy data analyses including CR rate, ORR, endpoints based on response (DOR, EFS, and PFS), relapse with CNS, and OS.

9.6. Planned Method of Analysis

The primary analysis will occur after all treated subjects have an opportunity to be assessed for response 6 months after the Week 4 disease assessment. The CSR will be written based on the data collected and analyzed from this primary analysis. The final analysis will occur after all subjects complete the study.

The primary analysis of CR rate will be based on the investigator's review of disease assessments in the response evaluable analysis set.

9.6.1. CR Rate

The subject incidence of CR will be calculated. The 2-sided 80%/90%/95% CIs will be provided about the CR rate, calculated with the Clopper-Pearson method.

9.6.2. ORR

The subject incidence of objective response will be calculated. The 2-sided 80%/90%/95% CIs will be provided about the ORR, calculated with the Clopper-Pearson method.

The incidence of subjects with CR, PR, stable disease, PD, not done, and not evaluable, as best overall response to treatment, and exact 2-sided 80%/90%/95% CIs about the incidence will be generated.

9.6.3. DOR

Kaplan-Meier plots, estimates, and 2-sided 80%/90%/95% CIs will be generated for DOR among the subjects who achieve an objective response. Kaplan-Meier estimates of the proportion of subjects alive and PFS at 3-month intervals will be provided. The number of subjects censored or having events and the reasons for censoring or type of events (PD or death) will be summarized.

A sensitivity analysis of DOR will be conducted in which disease assessments obtained after SCT (for subjects who undergo SCT while in an axicabtagene ciloleucel-induced response) will be used in the derivation of DOR.

DOR may be evaluated in subgroups defined by the covariates described in Section 9.1.1.

9.6.4. EFS

Kaplan-Meier plots, estimates, and 2-sided 80%/90%/95% CIs will be generated for EFS. Kaplan-Meier estimates of the proportion of subjects alive and event-free at 3-month intervals will be provided. The number of subjects censored or having events, and the reasons for censoring or type of events (PD, subsequent new antilymphoma therapy, or death) will be summarized.

EFS may be evaluated in subgroups defined by the covariates described in Section 9.1.1.

9.6.5. PFS

Kaplan-Meier plots, estimates, and 2-sided 80%/90%/95% CIs will be generated for PFS. Kaplan-Meier estimates of the proportion of subjects alive and progression-free at 3-month intervals will be provided. The number of subjects censored or having events, and the reasons for censoring or type of events (PD or death) will be summarized.

PFS may be evaluated in subgroups defined by the covariates described in Section 9.1.1.

9.6.6. OS

Kaplan-Meier plots, estimates, and 2-sided 80%/90%/95% CIs will be generated for OS. Estimates of the proportion of subjects alive at 3-month intervals will be provided through 2 years after the final subject has been enrolled and then annually through the completion of the study. The number of subjects censored or having events, and the reasons for censoring or type of events (death) will be summarized.

OS may be evaluated in subgroups defined by the covariates described in Section 9.1.1.

9.6.7. Relapse with CNS Disease

The number of subjects with CNS relapse and time to relapse with CNS disease among the subjects who experience CNS relapse will be summarized.

Relapse with CNS disease may be evaluated in subgroups defined by the covariates described in Section 9.1.1.

9.6.8. Safety

Subject incidence rates of AEs, including all, serious, fatal, CTCAE Grade 3 or higher, treatment-related AEs, and AEs of interest reported throughout the conduct of the study, will be tabulated by system organ class and preferred terms or preferred terms only, coded with the Medical Dictionary for Regulatory Activities. Changes in laboratory values and vital signs will be summarized with descriptive statistics.

The incidence of concomitant medications will be summarized.

Tables and/or narratives of deaths through the long-term follow-up and treatment-related SAEs will be provided.

The incidence, prevalence, duration, and reversibility of identified risks, RCR, and secondary malignancies will be summarized.

9.6.9. Pharmacokinetic Analysis

The levels of anti-CD19 CAR T cells measured in peripheral blood at Day 7 after axicabtagene ciloleucel infusion will be summarized with descriptive statistics.

9.6.10. Pharmacodynamics Analyses

The levels of cytokines in serum will be summarized with descriptive statistics.

10. REFERENCES

Barrington SF, Mikhaeel NG, Kostakoglu L, Meignan M, Hutchings M, Mueller SP, et al. Role of imaging in the staging and response assessment of lymphoma: consensus of the International Conference on Malignant Lymphomas Imaging Working Group. J Clin Oncol 2014;32 (27):3048-58.

Casasnovas RO, Ysebaert L, Thieblemont C, Bachy E, Feugier P, Delmer A, et al. FDG-PET-driven consolidation strategy in diffuse large B-cell lymphoma: final results of a randomized phase 2 study. Blood 2017;130 (11):1315-26.

Cheson BD, Fisher RI, Barrington SF, Cavalli F, Schwartz LH, Zucca E, et al. Recommendations for initial evaluation, staging, and response assessment of Hodgkin and non-Hodgkin lymphoma: the Lugano classification. J Clin Oncol 2014;32 (27):3059-68.

Cheson BD, Pfistner B, Juweid ME, Gascoyne RD, Specht L, Horning SJ, et al. Revised response criteria for malignant lymphoma. J Clin Oncol 2007;25 (5):579-86.

de Oliveira Costa R, Hallack Neto A, Siqueira S, Lage LA, de Paula HM, Coutinho AM, et al. Interim Fluorine-18 Fluorodeoxyglucose PET-Computed Tomography and Cell of Origin By Immunohistochemistry Predicts Progression-Free and Overall Survival in Diffuse Large B-Cell Lymphoma Patients in the Rituximab Era. Nucl Med Commun 2016;37 (10):1095-101.

Itti E, Meignan M, Berriolo-Riedinger A, Biggi A, Cashen AF, Vera P, et al. An International Confirmatory Study of the Prognostic Value of Early PET/CT in Diffuse Large B-cell Lymphoma: Comparison between Deauville Criteria and DeltaSUVmax. Eur J Nucl Med Mol Imaging 2013;40 (9):1312-20.

Locke F, Ghobadi A, Lekakis L, Miklos D, Jacobson C, E. J, et al. Outcomes by prior lines of therapy (LoT) in ZUMA-1, the pivotal phase 2 study of axicabtagene ciloleucel (Axi-Cel) in patients (Pts) with refractory large B cell lymphoma. J Clin Oncol 2018;Abstract # 3039.

Mamot C, Klingbiel D, Hitz F, Renner C, Pabst T, Driessen C, et al. Final Results of a Prospective Evaluation of the Predictive Value of Interim Positron Emission Tomography in Patients With Diffuse Large B-Cell Lymphoma Treated With R-CHOP-14 (SAKK 38/07). J Clin Oncol 2015;33 (23):2523-9.

Neelapu SS, Locke FL, Bartlett NL, Lekakis LJ, Miklos DB, Jacobson CA, et al. Axicabtagene Ciloleucel CAR T-Cell Therapy in Refractory Large B-Cell Lymphoma. N Engl J Med 2017.

Swerdlow SH, Campo E, Pileri SA, Harris NL, Stein H, Siebert R, et al. The 2016 revision of the World Health Organization classification of lymphoid neoplasms. Blood 2016;127 (20):2375-90.

Zhu D, Xu XL, Fang C, Ji M, Wu J, Wu CP, et al. Prognostic Value of Interim (18)F-FDG-PET in Diffuse Large B Cell Lymphoma Treated with Rituximab-Based Immune-Chemotherapy: A Systematic Review and Meta-Analysis. Int J Clin Exp Med 2015;8 (9):15340-50.

11. APPENDICES

Appendix 1. Lugano Classification {Cheson 2014}
Appendix 2. International Prognostic Index in Aggressive Lymphomas
Appendix 3. Birth Control Methods Which May Be Considered as Highly Effective

Appendix 1. Lugano Classification {Cheson 2014}

Please refer to {Cheson 2014} for details of assessment.

Deauville 5-Point Scale (5PS) {Barrington 2014}

Score	Description
1	No uptake above background
2	Uptake ≤ mediastinum
3	Uptake > mediastinum but ≤ liver
4	Uptake moderately higher than liver
5	Uptake markedly higher than liver and/or new lesions
X	New areas of uptake unlikely to be related to lymphoma

Complete Remission:

<u>Complete Metabolic Response for Positron Emission Tomography–Computed Tomography-Based Response</u>

The designation of complete metabolic response requires all of the following:

- A 5PS (5-point scale) score of 1, 2 or 3, with or without a residual mass
  - In Waldeyer's ring or extranodal sites with high physiologic uptake or with activation within spleen or marrow, uptake may be greater than normal in the mediastinum and/or liver. In this circumstance, complete metabolic response may be inferred if uptake at sites of initial involvement is no greater than surrounding normal tissue even if the tissue has high physiologic uptake.
- No new sites of disease should be observed
- No evidence of fluorodeoxyglucose (FDG)-avid disease in bone marrow

Complete Radiologic Response for Computed Tomography-Based Response

The designation of complete radiologic response requires all of the following:

- Target nodes/nodal masses must regress to ≤ 1.5 cm in longest transverse diameter (LDi) of a lesion
- No extralymphatic sites of disease
- Absent nonmeasured lesion

- Organ enlargement regress to normal
- No new sites of disease should be observed
- Bone marrow normal by morphology; if indeterminate, immunohistochemistry negative

Partial Remission:

<u>Partial Metabolic Response for Positron Emission Tomography–Computed Tomography-Based Response</u>

The designation of partial metabolic response requires all of the following:

- A 5PS score of 4 or 5, with reduced uptake compared to baseline (screening), and residual mass(es) of any size

  Note:

  - At interim, these findings suggest responding disease
  - At end of treatment, these findings suggest residual disease
- No new sites of disease should be observed
- Residual uptake is higher than uptake in normal bone marrow but reduced compared with baseline (diffuse uptake is compatible with reactive changes from chemotherapy allowed)

If there are persistent focal changes in the marrow in the context of a nodal response, consideration should be given to further evaluation with magnetic resonance image or biopsy or an interval scan.

<u>Partial Radiologic Response for Computed Tomography-Based Response</u>

The designation of partial radiologic response requires all of the following:

- ≥ 50% decrease in sum of the product of the perpendicular diameters of up to 6 target measurable nodes and extranodal sites
  - When a lesion is too small to measure on a computed tomography scan, assign 5 mm x 5 mm as the default value
  - When no longer visible, 0 x 0 mm
  - For a node > 5 mm x 5 mm, but smaller than normal, use actual measurement for calculation
- Absent/normal, regressed, but no increase of nonmeasured lesions
- Spleen must have regressed by > 50% in length beyond normal
- No new sites of disease should be observed

Stable Disease:

<u>No Metabolic Response for Positron Emission Tomography–Computed Tomography-Based Response</u>

The designation of no metabolic response requires all of the following:

- A 5PS score of 4 or 5, with no significant change in FDG uptake compared to baseline (screening) at an interim time point or end of treatment
- No new sites of disease should be observed
- No change from baseline in bone marrow

<u>Stable Radiologic Disease for Computed Tomography-Based Response</u>

The designation of stable radiologic disease requires all of the following:

- < 50% decrease from baseline in the sum of the product of the perpendicular diameters of up to 6 dominant, measurable nodes and extranodal sites; no criteria for progressive disease are met
- No increase consistent with progression in nonmeasured lesion and organ enlargement
- No new sites of disease should be observed

Progressive Disease:

<u>Progressive Metabolic Disease for Positron Emission Tomography–Computed Tomography-Based Response</u>

The designation of progressive metabolic disease requires at least 1 of the following:

- A 5PS score 4 or 5 with an increase in intensity of uptake from baseline and/or
- New FDG-avid foci consistent with lymphoma at interim or end of treatment assessment
- New FDG-avid foci consistent with lymphoma rather than another etiology (eg, infection, inflammation). If uncertain regarding etiology of new lesions, biopsy or interval scan may be considered
- New or recurrent FDG-avid foci in bone marrow

Progressive Radiologic Disease for Computed Tomography-Based Response

The designation of progressive radiologic disease requires at least one of the following:

- An individual node/lesion must be abnormal with:
  - LDi > 1.5 cm and
  - Increase by ≥ 50% from cross product of LDi and perpendicular diameter nadir and
  - An increase in LDi or SDi, shortest axis perpendicular to the LDi, (SDi) from nadir
    - 0.5 cm for lesions ≤ 2 cm
    - 1.0 cm for lesions > 2 cm
  - In the setting of splenomegaly, the splenic length must increase by > 50% of the extent of its prior increase beyond baseline (eg, a 15-cm spleen must increase to > 16 cm). If no prior splenomegaly, spleen must increase by at least 2 cm from baseline
  - New or recurrent splenomegaly
- New or clear progression of pre-existing nonmeasured lesions
- New lesion
  - Regrowth of previously resolved lesions
  - A new node > 1.5 cm in any axis
  - A new extranodal site > 1.0 cm in any axis; if < 1.0 cm in any axis, its presence must be unequivocal and must be attributable to lymphoma
  - Assessable disease of any size unequivocally attributable to lymphoma
- New or recurrent bone marrow involvement

Appendix 2. International Prognostic Index in Aggressive Lymphomas

The International Prognostic Index (IPI) score is calculated by adding the number of prognostic factors:

- Age > 60 years
- Performance status ≥ 2
- Lactate dehydrogenase 1× normal
- Extranodal sites > 1
- Stage III or IV

The IPI risk level is determined based on the IPI score:

Risk Level	IPI Score
Low (L)	0 or 1
Low-intermediate (LI)	2
High-intermediate (HI)	3
High (H)	4 or 5

Appendix 3. Birth Control Methods Which May Be Considered as Highly Effective

For the purpose of this guidance, methods that can achieve a failure rate of < 1% per year when used consistently and correctly are considered as highly effective birth control methods. Such methods include:

- Combined (estrogen and progesterone containing) hormonal contraception associated with inhibition of ovulation[1]:
  - Oral
  - Intravaginal
  - Transdermal
- Progestogen-only hormonal contraception associated with inhibition of ovulation[2]:
  - Oral
  - Injectable
  - Implantable[2]
- Intrauterine device[2]
- Intrauterine hormone-releasing system[2]
- Bilateral tubal occlusion[2]
- Vasectomized partner [2,3]
- Sexual abstinence[4]

1 Hormonal contraception may be susceptible to interaction with the investigational product, which may reduce the efficacy of the contraception method.
2 Contraception methods that in the context of this guidance are considered to have low user dependency.
3 Vasectomized partner is a highly effective birth control method provided that partner is the sole sexual partner of the woman of childbearing potential trial subject and that the vasectomized partner has received medical assessment of the surgical success.
4 In the context of this guidance sexual abstinence is considered a highly effective method only if defined as refraining from heterosexual intercourse during the entire period of risk associated with the study treatments. The reliability of sexual abstinence needs to be evaluated in relation to the duration of the clinical trial and the preferred and usual lifestyle of the subject.

**Sponsor:**	Kite Pharma, Inc.<br>2400 Broadway<br>Santa Monica, CA 90404<br>United States of America
**Product Name:**	**Axicabtagene ciloleucel**
**Protocol**	**A Phase 2 Multicenter Study Evaluating the Efficacy and Safety of Axicabtagene Ciloleucel as First-Line Therapy in Subjects with High-Risk Large B-Cell Lymphoma (ZUMA-12)**
**Protocol Number:**	**KTE-C19-112**
**Version Number:**	2.0
**Release Date:**	25 SEPTEMBER 2019
**Replaces Previous Version(s):**	1.0 27 July 2018

TABLE OF CONTENTS

TABLE OF CONTENTS 2
LIST OF TABLES 3
LIST OF FIGURES 3
LIST OF ABBREVIATIONS AND DEFINITIONS OF TERMS 4
1. INTRODUCTION 6
2. OBJECTIVES 7
3. STUDY DESIGN 8
3.1. Overview 8
3.2. Hypothesis 11
3.3. Sample Size Consideration 11
4. STUDY ENDPOINTS AND COVARIATES 12
4.1. Endpoints 12
4.1.1. Primary Endpoint 12
4.1.2. Secondary Endpoints 12
4.2. Covariates and Subgroups 13
5. DEFINITIONS 14
5.1. General 14
5.2. Safety 14
5.3. Efficacy 17
6. ANALYSIS SUBSETS 20
6.1. Safety Analysis Set 20
6.2. Full Analysis Set 20
6.3. Response Evaluable Analysis Set 20
6.4. Safety Retreatment Analysis set 20
6.5. Subgroup Analysis Sets 20
7. INTERIM ANALYSIS AND EARLY STOPPING GUIDELINES 21
8. DATA SCREENING AND ACCEPTANCE 22
8.1. General Principles 22
8.2. Electronic Transfer and Archiving of Data 22
8.3. Handling of Missing and Incomplete Data 22
8.3.1. Efficacy 22
8.3.2. Safety 22
8.4. Detection of Bias 22
8.5. Outliers 23
8.6. Distributional Characteristics 23
8.7. Validation and Configuration Management 23
9. STATISTICAL METHODS OF ANALYSIS 24
9.1. General Principles 24
9.2. Subject Accountability 24
9.3. Important Protocol Deviations 24
9.4. Demographic and Baseline Characteristics 24
9.5. Efficacy Analyses 25
9.5.1. CR rate 25

9.5.2. ORR and Best Overall Response (BOR) 26
9.5.3. DOR 26
9.5.4. EFS 26
9.5.5. PFS 26
9.5.6. OS 27
9.5.7. Relapse with CNS Disease 27
9.5.8. Tumor Burden 27
9.5.9. ORR and BOR among Subjects Retreated with axicabtagene ciloleucel 27
9.5.10. DORR 27
9.6. Safety Analyses 28
9.6.1. Adverse Events 28
9.7. Pharmacokinetics 30
9.8. Pharmacodynamics 30
9.9. Subsequent Anti-Cancer Therapy and SCT 30
10. CHANGES FROM PROTOCOL-SPECIFIED ANALYSES 31
11. REFERENCES 32
12. APPENDICES 33

Appendix 1. Conventions for Clinical Data that Require Imputation for Partial or Missing Dates 34
Appendix 2. Derivation of Time to Event Endpoints and Last Date Known to Be Alive 36

LIST OF TABLES

Table 1. Lower and upper limits of 80/95% CIs for CR rates from 60% to 100% for a sample of 40 subjects 11
Table 2. Imputation Rules for Partial or Missing Start Dates 34

LIST OF FIGURES

Figure 1. Study Schema for Study KTE-C19-112 10

LIST OF ABBREVIATIONS AND DEFINITIONS OF TERMS

Abbreviation	Definition
ADaM	Analysis data model
AE	Adverse event
ASCT	Autologous stem cell transplant
BOR	Best overall response
BSA	Body surface area
CAR	Chimeric antigen receptor
CI	Confidence Interval
CNS	Central nervous system
CR	Complete response
CRF	Case report form
CRS	Cytokine release syndrome
CSF	Cerebrospinal fluid
CSR	Clinical study report
CTCAE	Common terminology criteria for adverse event
DOR	Duration of response
DORR	Duration of response to retreatment
DLBCL	Diffuse large B-cell lymphoma
DSMB	Data safety monitoring board
ECOG	Eastern Cooperative Oncology Group
EFS	Event-free survival
FAS	Full Analysis Set
GVHD	Graft-Versus-Host-Disease
IPI	International Prognostic Index
MedDRA	Medical Dictionary for Regulatory Activities
MST	MedDRA search terms
NCI	National Cancer Institute
ND	Not done
NE	Not evaluable
ORR	Objective response rate
OS	Overall survival
PET-CT	Positron emission tomography-computed tomography

Abbreviation	Definition
PFS	Progression-free survival
PR	Partial response
PT	Preferred term
SAP	Statistical analysis plan
SCT	Stem cell transplant
SD	Stable disease
SDTM	Study data tabulation model
SMQ	Standardized MedDRA query
SOC	System organ class
SUSAR	Suspected unexpected serious adverse reactions
TEAE	Treatment-emergent adverse event
WHO	World Health Organization

1. INTRODUCTION

This statistical analysis plan (SAP) provides the pre-specification and details for the statistical analyses to support the protocol KTE-C19-112 (ZUMA-12) entitled “A Phase 2 Multicenter Study Evaluating the Efficacy and Safety of Axicabtagene Ciloleucel as First-Line Therapy in Subjects with High-Risk Large B-Cell Lymphoma”. The scope of this document is to provide details on the planned interim, primary, and final analyses.

2. OBJECTIVES

The primary objective of the analyses outlined herein is to estimate the efficacy of axicabtagene ciloleucel, as measured by CR rate, in subjects with high-risk large B-cell lymphoma, as determined by study investigators.

Secondary objectives are to characterize the safety profile, and to further characterize efficacy with secondary endpoints; further secondary objectives will include pharmacokinetic/pharmacodynamics endpoints.

3. STUDY DESIGN

3.1. Overview

Study KTE-C19-112 is a Phase 2, multicenter, single arm, open-label study evaluating the efficacy and safety of axicabtagene ciloleucel as first-line therapy in subjects with high-risk large B-cell lymphoma.

Approximately 40 subjects with high-risk large B-cell lymphoma, including either high-grade B-cell lymphoma (HGBL) with MYC and BCL2 and/or BCL6 translocations (double-/triple-hit lymphomas), or large B-cell lymphoma with a high-intermediate/ high-risk International Prognostic Index (IPI) score ≥ 3, will be enrolled and treated with cyclophosphamide and fludarabine conditioning chemotherapy, followed by a target dose of 2 x 10^6 anti-CD19 chimeric antigen receptor (CAR) T cells/kg body weight.

Each subject with a positive interim positron emission tomography-computed tomography (PET-CT) per the Lugano Classification (Cheson et al, 2014) (Deauville 5-point scale PET score of 4 or 5) after 2 cycles (PET2+) of standard-of-care chemoimmunotherapy will proceed through the following study periods:

- Screening
- Enrollment/Leukapheresis
- Non-chemotherapy bridging therapy (if applicable)
- Conditioning chemotherapy period
- Investigational Product (IP) treatment period
- Post treatment assessment period
- Long term follow-up period

For study requirements assigned to each study period, refer to protocol Section 7 for details.

At specific time points, subjects will undergo the following assessments/procedures: collection of informed consent, general medical history including previous treatments for large B-cell lymphoma, physical exam including vital signs and performance status, neurologic assessments, cytokines, and anti-CD19 CAR T cell analysis. Subjects will also undergo a baseline PET-CT, and leukapheresis. Subjects may also need bone marrow aspirate/biopsy.

Routinely throughout the conduct of the study, subjects will be asked to report concomitant medications and adverse events and will have their disease assessed.

The primary endpoint is complete response (CR) rate, defined as the incidence of a CR per the Lugano Classification (Cheson et al, 2014), as determined by study investigators. All evaluable subjects who do not meet the criteria for a CR by the analysis data cutoff date will be considered non-complete responders.

Further details on study procedures may be found in the study protocol. A study schema is present in Figure 1.

Figure 1. Study Schema for Study KTE-C19-112

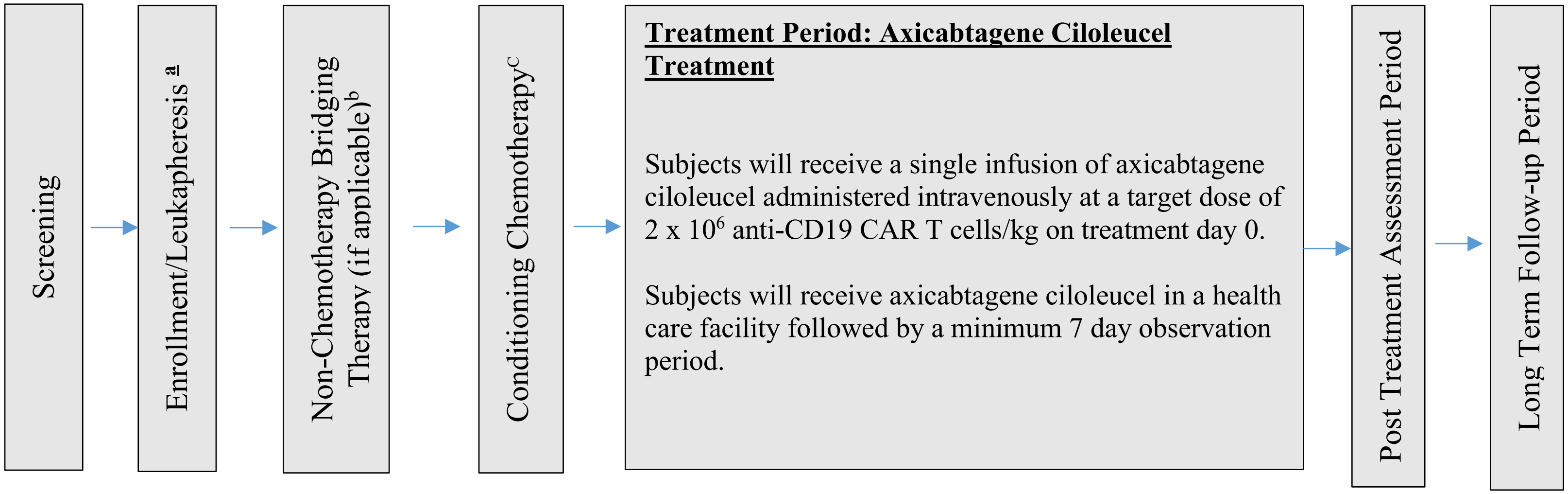

Approximately 40 subjects who are either double hit/triple hit or have IPI ≥ 3 will be enrolled and treated.

[a] **Enrollment/Leukapheresis:** Subjects who have a positive interim PET per the Lugano Classification {Cheson 2014} (Deauville PET score of 4 or 5) after 2 cycles (PET2+) of an anti-CD20 monoclonal antibody and anthracycline-containing regimen per local standard of care (eg, DA-EPOCH-R) if double hit/triple hit, or an anti-CD20 monoclonal antibody and anthracycline-containing regimen per local standard of care (eg, R-CHOP) if large B-cell lymphoma with IPI score ≥ 3.

[b] **Non-Chemotherapy Bridging Therapy:** At the discretion of the investigator, corticosteroid or HDMP + rituximab bridging therapy may be considered for subjects with high disease burden at screening or baseline assessments. Localized radiation therapy for symptom control may also be allowed, provided that the radiation field does not include a target lesion. Refer to Section 6.1.2 for details.

[c] **Conditioning Chemotherapy:** Subjects will receive a 3-day conditioning chemotherapy regimen consisting of fludarabine 30 mg/m^2/day and cyclophosphamide 500 mg/m^2/day (Day –5 to Day –3) followed by 2 rest days (Day –2 and Day –1).

3.2. Hypothesis

No formal hypothesis will be tested. This study is designed to estimate the CR rate in subjects with high-risk large B-cell lymphoma. The CR rate targeted in this study is 60%.

3.3. Sample Size Consideration

In the GELA randomized phase 2 study evaluating the efficacy of R-ACVBP or R-CHOP-14 induction, using IWF 2007 criteria in young patients with high-risk DLBCL, the primary objective of achieving a higher than 50% CR rate after 4 cycles of induction regime was not met in both randomization groups (Casasnovas et al, 2017). Accordingly, it is postulated that a CR rate of 60% would represent a clinically meaningful improvement over standard chemoimmunotherapy in a high-risk large B-cell lymphoma patient population. Although there is no formal hypothesis testing, the sample size has been determined in part with descriptive analysis described below.

The trial uses a single-arm design to estimate the CR rate in subjects with high-risk large B-cell lymphoma treated with axicabtagene ciloleucel. A CR rate of 60% with axicabtagene ciloleucel treatment is targeted. With a total sample size of 40 subjects, an observed CR rate of 60% will yield an 80% confidence interval (CI) for the response rate with a maximum half-width of less than or equal to 11%, corresponding to a lower limit of at least 48.6%. This target CR rate, and the lower limit of the 80% CI for the CR rate, is meaningful because it would represent a significant improvement in the response rate for the subjects with high-risk large B-cell lymphoma and would likely offer an improvement over existing therapies in patients with high-risk large B-cell lymphoma (Table 1).

Table 1 provides the estimated CR rate, and the lower and upper limits of 80/95% CIs based on the Clopper-Pearson method for a range of possible CR rate for a sample of 40 subjects.

Table 1. Lower and upper limits of 80/95% CIs for CR rates from 60% to 100% for a sample of 40 subjects

Number (%) of CRs	24 (60)	28 (70)	32 (80)	36 (90)	40 (100)
Lower and Upper Limits of 80% CI (%, %)	48.6, 70.6	58.8, 79.5	69.6, 88.0	81.0, 95.6	94.4, 100
Lower and Upper Limits of 95% CI (%, %)	43.3, 75.1	53.5, 83.4	64.4, 90.9	76.3, 97.2	91.2, 100

One planned interim analysis will be performed. Interim Analysis 1 will be conducted after 15 subjects have been enrolled and treated with axicabtagene ciloleucel, and have had an opportunity to be followed for 3 months after axicabtagene ciloleucel infusion. This analysis will be for efficacy and safety, and will be descriptive.

4. STUDY ENDPOINTS AND COVARIATES

4.1. Endpoints

4.1.1. Primary Endpoint

- CR rate, defined as the incidence of a CR per the Lugano Classification (Cheson et al, 2014) as determined by study investigators.

4.1.2. Secondary Endpoints

- Objective response rate (ORR), defined as the incidence of either a CR or a partial response (PR) per the Lugano Classification (Cheson et al, 2014) as determined by the study investigators

- Duration of response (DOR), defined only for subjects who experience an objective response after axicabtagene ciloleucel infusion and is the time from the first objective response to disease progression per the Lugano Classification (Cheson et al, 2014) or death from any cause.

- Event-free survival (EFS), defined as the time from the axicabtagene ciloleucel infusion date to the earliest date of disease progression per the Lugano Classification (Cheson et al, 2014), commencement of subsequent new anti-lymphoma therapy including SCT, or death from any cause.

- Progression-free survival (PFS), defined as the time from the axicabtagene ciloleucel infusion date to the date of disease progression per the Lugano Classification (Cheson et al, 2014) or death from any cause.

- Overall survival (OS), defined as the time from axicabtagene ciloleucel infusion to the date of death from any cause.

- Incidence of adverse events (including grade ≥3, serious, fatal, and adverse events of interest).

- Relapse with central nervous system (CNS) disease, defined only for subjects who experience CNS relapse and defined as the time from the axicabtagene ciloleucel infusion date to the earliest date of CNS involvement with lymphoma as determined by typical symptoms, CSF evaluation, and/or diagnostic imaging.

Additional secondary endpoints include pharmacokinetic/pharmacodynamic endpoints such as evaluation of levels of anti-CD19 CAR T cells in blood and levels of cytokines in serum.

4.2. Covariates and Subgroups

The following covariates at screening/baseline or on the study may be used to examine CR, ORR and other efficacy and safety endpoints in covariate or subgroups analyses:

- Age (< 65, ≥ 65 years)
- Gender
- Race
- Ethnicity
- ECOG status
- IPI score
- Diagnosis category (double-hit lymphomas vs triple-hit lymphomas vs non double-/triple-hit with IPI score ≥3), by both local and central pathology
- Levels of cytokines
- Levels of CAR T Cells
- CR achieved on the study

Covariate levels that are sparse may be collapsed for purposes of statistical modeling.

Additional associative analyses of covariates with subject outcomes may be explored.

5. DEFINITIONS

5.1. General

Study enrollment: Study enrollment occurs at the commencement of leukapheresis.

Study Day 0: Study Day 0 is defined as the day the subject received the first axicabtagene ciloleucel infusion. The day prior to Study Day 0 will be study day -1. The day of enrollment and any days after enrollment and before study day -1 will be sequential and negative integer-valued.

Baseline: The baseline value is defined as the last value taken prior to first dose of conditioning chemotherapy; if enrolled subjects do not receive conditioning chemotherapy, the baseline value is defined as the last value taken prior to enrollment/leukapheresis.

Study therapy: Study therapy is defined as conditioning chemotherapy or axicabtagene ciloleucel.

On-study: Time from enrollment to the last date of contact.

End of study: This will occur after all subjects treated with axicabtagene ciloleucel have been followed for 15 years post axicabtagene ciloleucel infusion, have withdrawn consent, been lost to follow-up, or have died.

Actual follow-up time: Actual follow-up time among all subjects treated with axicabtagene ciloleucel is calculated as the time from the first dose of axicabtagene ciloleucel to the date of death, last date known alive, lost to follow-up, or withdrawal of consent, whichever is later.

Potential follow-up time: Potential follow-up time is defined as the time from the axicabtagene ciloleucel infusion to the data cutoff date for the analysis.

Follow-up time for response: Follow-up time for response is derived as the time from the axicabtagene ciloleucel infusion date to the last disease assessment or censoring date. Follow-up time for response is derived using the reverse Kaplan-Meier approach in which the censoring times and event times are reversed to derive the median follow-up time.

5.2. Safety

Treatment-emergent adverse event (TEAE): Any adverse event with onset on or after the axicabtagene ciloleucel infusion. For subjects who receive retreatment with axicabtagene ciloleucel, TEAEs after retreatment may be summarized separately.

Deaths: All deaths that occur after leukapheresis up through the end of study.

Adverse events of interest: Adverse events of interest for axicabtagene ciloleucel treatment include adverse events in the categories of:

Important Identified risks:

- Cytokine-release syndrome (CRS)
- Neurologic toxicity
- Cytopenias, including
  - neutropenia
  - thrombocytopenia
  - anemia
- Hypogammaglobulinemia
- Infections

Important Potential risks:

- Secondary malignancies
- Tumor lysis syndrome
- Bone marrow failure
- Graft-Versus-Host-Disease (GVHD)
- Replication competent retrovirus (RCR)
- Immunogenicity (anti-axicabtagene ciloleucel antibodies)

Cytokine release syndrome (CRS): CRS as a syndrome (i.e. a collection of individual symptoms) is identified via collection of the syndrome on a case report form specifically designed to collect CRS. Individual symptoms of CRS are separately collected on the adverse events log and are linked to the CRS syndrome. CRS syndrome severity is graded according to a modification of the grading system proposed by Lee and colleagues (Lee et al, 2014). In the modified grading scale, neurologic AEs are not reported as part of the CRS syndrome; rather, they are reported on the AE log form separately based on specific symptoms per Common Terminology Criteria for Adverse Events (CTCAE).

Neurologic toxicity (Neurotoxicity): Neurologic adverse events will be identified with Medical Dictionary for Regulatory Activities (MedDRA) search terms (MST) search strategy based on known neurologic toxicities associated with anti-CD19 immunotherapy (Topp et al, 2015). The search strategy focuses on central nervous system (CNS) toxicity, without regard to temporal relationship and concomitant conditions (e.g. CRS). MedDRA system organ classes (SOCs) of Psychiatric Disorders and Nervous System Disorders will be reviewed for additional events.

These events will then be evaluated for potential inclusion as neurologic AEs. Neurologic toxicity will be reported separately from CRS.

Cytopenias (including aplastic anemia): Cytopenias (neutropenia or thrombocytopenia or anemia including aplastic anemia and bone marrow failure) are identified as:

- Thrombocytopenia will be identified using the standardized MedDRA query (SMQ) for haematopoietic thrombocytopenia (narrow search)
- Neutropenia will be identified using Kite-specified MedDRA search terms (MST)
- Anemia (including aplastic anemia) will be identified using the SMQ haematopoietic erythropenia (broad search)

Subjects with cytopenias (neutropenia or thrombocytopenia or anemia) present on or after Day 30 post axicabtagene ciloleucel infusion will be summarized separately by cell lineage.

Hypogammaglobulinemia: Hypogammaglobulinemia will be identified using a MST search strategy defined by Kite.

Infections: Infections are identified as adverse events within the system organ class (SOC) of Infections and Infestations that occur after treatment with axicabtagene ciloleucel and in MedDRA high level group terms (HLGTs) that capture events of:

1) Bacterial infection, encompassing PTs within the MedDRA HLGTs of
    a) bacterial infectious disorders
    b) chlamydial infectious disorders
2) Viral infection, encompassing PTs within the MedDRA HLGTs of viral infectious disorders
3) Opportunistic infections, encompassing PTs within the MedDRA HLGTs of
    a) fungal infectious disorders
    b) mycobacterial infectious disorders
4) Other infections, encompassing PTs within the MedDRA HLGTs of Infections – pathogen unspecified

Secondary malignancy: Adverse events that are coded into the SOC of Neoplasms benign, malignant, and unspecified (including cysts and polyps) will be reviewed to identify potential events. Additionally, adverse events that are coded into the SOC of Neoplasms benign, malignant, and unspecified (including cysts and polyps) will be reviewed to identify other potential events.

Tumor Lysis Syndrome: Tumor lysis syndrome will be identified as events with MedDRA PTs in the Tumor Lysis Syndrome SMQ (MedDRA). The narrow version of this SMQ will be used.

Bone marrow failure: Bone marrow failure will be identified using the narrow SMQ of haematopoietic cytopenias affecting more than one type of blood test.

Graft-Versus-Host Disease (GVHD): Graft-Versus-Host-Disease (GVHD) will be identified using a MST search strategy defined by Kite by using subsets of PT from HLGT of procedural related injuries and complications NEC and high level term (HLT) of immune and associated conditions NEC.

Immunogenicity (Anti-axicabtagene ciloleucel antibody): Immunogenicity will be identified for subjects who have treatment emergent anti-axicabtagene ciloleucel antibodies and have developed any AE belonging to the SMQ of anaphylactic reaction and the SMQ of hypersensitivity. The narrow version of these two SMQs will be used.

Time to Onset of Event/Syndrome: Time to onset of an event/syndrome is defined as the time from study day 0 to the day of the first occurrence of the event/syndrome. Time to Onset of Grade 3 or Higher Events/Syndromes are defined in the same way, but restricted to Grade 3 or higher events/syndromes.

Duration of Event/Syndrome: The duration across all events is the last day of the last event – first day of the first event +1, regardless of whenever the events are consecutive, overlapping, or neither.

Durations of events will not be calculated for events that are ongoing at the time of the data cutoff date or subject death. For events defined by laboratory criteria, time to onset and duration will not be calculated. For events defined by both laboratory criteria and adverse events, only the adverse event component will be used to define time to onset and duration.

5.3. Efficacy

CR rate: CR rate is defined as the incidence of a CR per the Lugano Classification (Cheson et al, 2014) as determined by study investigators. All evaluable subjects who do not meet the criteria for a CR by the analysis data cutoff date will be considered non-complete responders. The derivation of this endpoint will only include response assessments obtained after the initial axicabtagene ciloleucel infusion and up to progressive disease (PD)/death due to any cause or the disease assessments prior to any subsequent anti-lymphoma therapy including stem cell transplant (SCT) or retreatment with axicabtagene ciloleucel.

ORR: ORR is defined as the incidence of either a CR or a PR per the Lugano Classification (Cheson et al, 2014) as determined by the study investigators. All evaluable subjects who do not meet the criteria for an objective response by the analysis cutoff date will be considered non-responders, including the subjects with nonevaluable assessment data and those without any assessment. The derivation of this endpoint will only include response assessments obtained after the initial axicabtagene ciloleucel infusion and up to PD/death due to any cause or the disease assessments prior to any subsequent anti-lymphoma therapy including SCT or retreatment with axicabtagene ciloleucel

DOR: DOR is defined only for subjects who experience an objective response (CR or PR) after axicabtagene ciloleucel infusion and is the time from the first objective response to disease progression per the Lugano Classification (Cheson et al, 2014) or death due to any cause. Subjects not meeting the criteria for disease progression or death by the analysis data cutoff date will be censored at their last evaluable disease assessment date. The DOR for subjects who receive any subsequent anti-lymphoma therapy including SCT or retreatment with axicabtagene ciloleucel in the absence of documented disease progression or death due to any cause will be censored at the last evaluable disease assessment prior to the start date of the subsequent anti-lymphoma therapy. Further details on the derivation of DOR are provided in Appendix 2.

EFS: EFS is defined as the time from the axicabtagene ciloleucel infusion date to the earliest date of disease progression per the Lugano Classification (Cheson et al, 2014), commencement of subsequent anti-lymphoma therapy including SCT or retreatment with axicabtagene ciloleucel, or death due to any cause. Subjects alive, in response, and with no new anti-lymphoma therapy including SCT or retreatment with axicabtagene ciloleucel will be censored at the last evaluable disease assessment. Further details on the derivation of EFS are provided in Appendix 2.

PFS: PFS is defined as the time from the axicabtagene ciloleucel infusion date to the date of disease progression per the Lugano Classification (Cheson et al, 2014) or death due to any cause. Subjects not meeting the criteria for disease progression or death from any cause by the analysis cutoff date will be censored at the last evaluable disease assessment. The PFS for subjects who receive any subsequent anti-lymphoma therapy including SCT or retreatment with axicabtagene ciloleucel in the absence of documented disease progression or death due to any cause will be censored at the last evaluable disease assessment prior to the start date of the subsequent anti-lymphoma therapy. Further details on the derivation of PFS are provided in Appendix 2.

OS: OS is defined as the time from the axicabtagene ciloleucel infusion to the date of death due to any cause. Subjects who are alive by the analysis data cutoff date will be censored at their last date known to be alive or the data cutoff date, whichever is earlier. Subjects who die after the data cutoff date will be censored at the data cutoff date. Further details on the derivation of overall survival and the specific data modules that will be used to derive the last date known to be alive are provided in Appendix 2.

Relapse with CNS: relapse with CNS disease is defined only for subjects who experience CNS relapse. It is defined as the time from the axicabtagene ciloleucel infusion date to the earliest date of CNS involvement with lymphoma as determined by typical symptoms, CSF evaluation, and/or diagnostic imaging.

Duration of response to retreatment (DORR): DORR is defined only for subjects who receive retreatment following progression of disease and then go on to experience an objective response to retreatment. It is defined as the time from the first objective response after retreatment to disease progression per the Lugano Classification (Cheson et al, 2014) or death due to any cause. Subjects not meeting the criteria for disease progression after retreatment or death due to any cause by the analysis data cutoff date will be censored at their last evaluable disease assessment date after retreatment. DORR will be derived using disease assessments obtained on study prior

to the start date of any subsequent anti-lymphoma therapy including SCT. Further details on the derivation of DORR are provided in Appendix 2.

6. ANALYSIS SUBSETS

In this study, subjects are to be dosed at a target of 2×10^6 (1.0×10^6 to 2.4×10^6) anti-CD19 CAR T cells/kg. A minimum dose of 1×10^6 anti-CD19 CAR T cells/kg may be administered. For subjects weighing ≥ 100 kg, a maximum flat dose of 2×10^8 anti-CD19 CAR T cells will be administered. Subjects are considered to have received the target dose if they receive at least 1×10^6 anti-CD19 CAR T cells/kg.

6.1. Safety Analysis Set

The safety set is defined as all subjects treated with any dose of axicabtagene ciloleucel. This analysis set will be used for all safety data analyses.

6.2. Full Analysis Set

The full analysis set (FAS) will consist of all enrolled/leukapheresed subjects and will be used for the summary of subject disposition and the listing of deaths.

6.3. Response Evaluable Analysis Set

The response evaluable analysis set will consist of the subjects who are enrolled and treated with axicabtagene ciloleucel at a dose of at least 1×10^6 anti-CD19 CAR T cells/kg, and centrally confirmed disease type (double-/triple- hit lymphomas) or IPI score ≥ 3. This analysis set will be used for all efficacy data analyses including CR rate and ORR, and endpoints based on response (DOR, EFS, PFS), relapse with CNS, and OS.

6.4. Safety Retreatment Analysis set

The safety retreatment analysis set will consist of all subjects who undergo retreatment with axicabtagene ciloleucel. This set will be used for all retreatment safety and efficacy analyses.

6.5. Subgroup Analysis Sets

Subgroup analyses of selected efficacy and safety endpoints may be performed for the covariates and subgroups defined in Section 4.2.

7. INTERIM ANALYSIS AND EARLY STOPPING GUIDELINES

The data safety monitoring board (DSMB) will meet and review the serious adverse events (SAEs) and suspected unexpected serious adverse reactions (SUSARs) on at least a semi-annual basis after the first subject has been treated with axicabtagene ciloleucel up through the primary analysis of the study. The DSMB will also review SAEs and SUSARs on a regular basis up through the primary analysis.

One planned interim analysis will be performed. At Interim Analysis 1, the DSMB will meet to review safety data after 15 subjects have been enrolled and treated with axicabtagene ciloleucel and have had an opportunity to be followed for 3 months after axicabtagene ciloleucel infusion.

The DSMB will make trial conduct recommendations on an ongoing basis based on an analysis of risk vs benefit. The DSMB may request additional safety data or may recommend modifying the study conduct if safety concerns are identified. Data submitted to the DSMB may be monitored or unmonitored to facilitate and ensure timely DSMB review.

8. DATA SCREENING AND ACCEPTANCE

8.1. General Principles

The database will be subject to the edit checks outlined in the Data Management Plan and additional manual data reviews defined by the study team. Data inconsistencies will be reviewed and resolved before the database snapshot for the primary analyses and the final database lock. For interim analyses, snapshots may include data that has not passed all data cleaning procedures at the time the data are extracted for snapshot.

8.2. Electronic Transfer and Archiving of Data

The database for this study will be managed and maintained by Kite Pharma. Raw data, Study Data Tabulation Model (SDTM) data, and Analysis Data Model (ADaM) datasets will be generated by Kite Pharma, Inc. and will be archived for all planned analyses. Any additional unplanned analyses that occur after the primary analyses and prior to the final analysis will also be archived.

Data from the central pathology laboratory, the product characteristics central laboratory assessment of subject serum samples (CAR T cell levels in the peripheral blood, antibody assays, RCR testing) will be generated from contract laboratories and Kite Pharma. These data will be transferred to Kite held in a peripheral directory and not built into the clinical trial database. At the time analyses require these data, they may be merged with the SDTM and ADaM datasets.

8.3. Handling of Missing and Incomplete Data

8.3.1. Efficacy

The method for handling missing data is described in the definition for each efficacy endpoint. Every effort will be made to obtain complete dates for deaths. In the event of a partial or missing death date and the corresponding censoring date for survival, the algorithm in Appendix 2 will be used.

8.3.2. Safety

Partial adverse event start dates will be imputed. If dates are missing or incomplete for adverse event start dates, the algorithm defined in Appendix 1 will be used. Completely missing death dates or death dates with only a year reported will not be imputed.

8.4. Detection of Bias

A listing of subjects with important protocol deviations will be generated. The deviations included in this list will include violations of eligibility criteria and use of exclusionary medication during the study. Lack of protocol compliance will be evaluated by summarizing the subject incidence of important protocol deviations. High rates of important protocol deviations may indicate bias.

8.5. Outliers

Descriptive statistics will be used to identify potential outliers in any key variables analyzed. Suspected outliers will be included in all analyses unless there is sufficient scientific justification to exclude them.

8.6. Distributional Characteristics

The goal of the primary statistical analysis is to estimate CR rate. All 80/90/95% confidence intervals will be 2-sided and calculated via the Clopper-Pearson method. This test assumes only the independence of the individual subject responses.

8.7. Validation and Configuration Management

Programs for the development of the SDTM and ADaM datasets and the generation of the tables, figures, and listings will be developed and maintained according to Kite Pharma Standard Operating Procedures. The software and version used to generate analyses will be indicated in the archived documentation.

9. STATISTICAL METHODS OF ANALYSIS

9.1. General Principles

The primary analysis will occur after all treated subjects have an opportunity to be assessed for response 6 months after the Week 4 disease assessment. The clinical study report (CSR) will be written based on the data collected and analyzed from this primary analysis. The final analysis will occur after all subjects complete the study. The primary analysis of CR rate will be based on investigator review of disease assessments in the response evaluable analysis set.

9.2. Subject Accountability

The number of subjects screened, enrolled, leukapheresed, treated with conditioning chemotherapy, treated with axicabtagene ciloleucel, and retreated with axicabtagene ciloleucel will be summarized. The reasons for discontinuing treatment and the disease assessment and survival follow-up periods will be summarized.

Summaries of actual follow up time will be provided.

The number of subjects enrolled by country and site will be summarized.

The number of subjects in each analysis set along with reasons for exclusion will be provided.

9.3. Important Protocol Deviations

The clinical study team will define important protocol deviation categories and review all potential important protocol deviations at minimum, prior to the database snapshot for the primary efficacy analysis. Important protocol deviations will be categorized by deviation type (e.g. entry/eligibility, use of excluded medication, etc). The subject incidence of important protocol deviations will be summarized overall and by deviation category.

9.4. Demographic and Baseline Characteristics

Summary statistics and frequencies for the following demographic and baseline characteristics will be tabulated:

- Age (< 65, ≥ 65)
- Race: white, Asian, other (categories may be collapsed or expanded based on accrual)
- Ethnicity (Hispanic or Latino, Not Hispanic or Latino)
- Sex (male, female)
- Weight
- Height

- IPI score
- ECOG performance status
- Diagnosis category (double-hit lymphomas vs triple-hit lymphomas vs non double-/triple-hit with IPI score ≥3). Double/triple hit status will be summarized per investigator and per central pathology.
- Disease stage
- Disease extent (presence of B symptoms, splenic involvement, extranodal disease, bulky disease)
- Bone marrow involvement

9.5. Efficacy Analyses

For the primary analysis, the investigator assessment status per the Lugano Classification (Cheson et al, 2014) for CR rate will be used. The investigator reviewer will provide the determination of disease status (CR, PR, stable disease [SD], progressive disease [PD], not evaluable [NE], not done [ND]) at each time point. SAS programs developed by Kite Pharma will derive the best overall response (BOR), DOR, EFS, and PFS based on these assessments.

The primary efficacy analysis will be presented in the response evaluable analysis set.

For subjects retreated with axicabtagene ciloleucel, disease assessments obtained prior to retreatment but not disease assessment obtained after retreatment will be included in the primary summaries of ORR and BOR, DOR, EFS, PFS, and summaries of change in tumor burden (if applicable). For such retreated subjects, disease assessments obtained after retreatment will be included in the summaries of ORR, BOR rate to retreatment with axicabtagene ciloleucel, and DOR after retreatment with axicabtagene ciloleucel.

In the event any subject undergoes any subsequent antilymphoma therapy (including SCT or retreatment with axicabtagene ciloleucel) while on study, the subject's BOR and change in tumor burden will be derived only based on disease outcomes assessed prior to initiation of the subsequent anti-lymphoma therapy (including SCT or retreatment with axicabtagene ciloleucel), whichever is earlier. For subjects without documentation of disease progression prior to initiation of the subsequent anti-lymphoma therapy (including SCT or retreatment with axicabtagene ciloleucel), DOR, EFS, and PFS time will be censored at the last disease assessment prior to the initiation of subsequent anti-lymphoma therapy.

9.5.1. CR rate

9.5.1.1. Primary Analyses of CR Rate

The subject incidence of CR will be calculated. The 2-sided 80/90/95% CIs will be provided about the CR rate, calculated with the Clopper-Pearson method.

9.5.1.2. Subgroup Analyses of CR

CR rate, and 95% CIs about CR rate will be generated for subgroups defined in Section 4.2.

A forest plot of the proportion of responders for each of these groups will be generated.

9.5.2. ORR and Best Overall Response (BOR)

9.5.2.1. Primary Analyses of ORR and BOR

The subject incidence of objective response will be calculated. The 2-sided 80/90/95% CIs will be provided about the ORR, calculated with the Clopper-Pearson method.

The incidence of subjects with PR, SD, PD, ND and NE, as BOR to treatment and exact 2-sided 80/90/95% CIs about the incidence will also be generated.

9.5.2.2. Subgroup Analyses of ORR

ORR, and 95% CIs about ORR will be generated for subgroups defined in Section 4.2.

A forest plot of the proportion of responders for each of these groups will be generated.

9.5.3. DOR

Kaplan-Meier plots, estimates, and 2-sided 80/90/95% CIs will be generated for DOR among the subjects who achieve an objective response. Kaplan-Meier estimates of the proportion of subjects alive and progression-free at 3-month intervals will be provided. The number of subjects censored or having events, and the reasons for censoring or type of events (PD or death) will be summarized.

A sensitivity analysis of DOR will be conducted in which disease assessments obtained after SCT (for subjects who undergo SCT while in an axicabtagene ciloleucel-induced response) will be used in the derivation of DOR.

DOR may be evaluated in subgroups defined by the covariates described in Section 4.2.

9.5.4. EFS

Kaplan-Meier plots, estimates, and 2-sided 80/90/95% CIs will be generated for EFS. Kaplan-Meier estimates of the proportion of subjects alive and event-free at 3-month intervals will be provided. The number of subjects censored or having events, and the reasons for censoring or type of events (PD, subsequent anti-lymphoma therapy, or death) will be summarized.

EFS may be evaluated in subgroups defined by the covariates described in Section 4.2.

9.5.5. PFS

Kaplan-Meier plots, estimates, and 2-sided 80/90/95% CIs will be generated for PFS. Kaplan-Meier estimates of the proportion of subjects alive and progression-free at 3-month intervals will

be provided. The number of subjects censored or having events, and the reasons for censoring or type of events (PD or death) will be summarized.

PFS may be evaluated in subgroups defined by the covariates described in Section 4.2.

9.5.6. OS

Kaplan-Meier plots, estimates and 2-sided 80/90/95% CIs will be generated for OS. Estimates of the proportion of subjects alive at 3-month intervals will be provided through 2 years after the final subject has been enrolled and then annually through the completion of the study. The number of subjects censored or having events, and the reasons for censoring or type of events (death) will be summarized.

OS may be evaluated in subgroups defined by the covariates described in Section 4.2.

9.5.7. Relapse with CNS Disease

The number of subjects with CNS relapse and time to relapse with CNS disease among the subjects who experience CNS relapse will be summarized.

Relapse with CNS disease may be evaluated in subgroups defined by the covariates described in Section 4.2.

9.5.8. Tumor Burden

The change in tumor burden, as measured by the sum of the products of the diameters of the selected lesions, from baseline to post-baseline nadir will be summarized in absolute numbers (mm^2) and percentage. A graphical summary of this change will be presented in a vertical bar chart with each subject's change from baseline to nadir displayed as a vertical bar, with color coding that indicates best response attained ("waterfall" plot). Summary statistics will be provided for this change. Additionally, plots over time of the percent change in tumor burden for each subject (superimposed on one graph) will be presented. Data collected after the subsequent anti-lymphoma therapy including SCT or retreatment with axicabtagene ciloleucel will not be included for the analyses.

9.5.9. ORR and BOR among Subjects Retreated with axicabtagene ciloleucel

The subject incidence of objective response and BOR (CR, PR, SD, PD, NE) to the retreatment among subjects retreated with axicabtagene ciloleucel will be calculated. Confidence intervals will be provided about the ORR and CR rate to the retreatment.

9.5.10. DORR

The analysis of DORR will use the same methods as the analysis of duration of response.

9.6. Safety Analyses

The primary analysis of safety data will summarize all treatment-emergent adverse events. For subjects who undergo retreatment with axicabtagene ciloleucel, adverse events occurring in the axicabtagene ciloleucel retreatment period may be summarized in an additional separate summary that presents only the AEs occurring during the axicabtagene ciloleucel retreatment period.

Adverse events will be coded with MedDRA. The version of the MedDRA may vary over time as the current version in use is updated. The severity of adverse events will be graded using the National Cancer Institute (NCI) Common Terminology Criteria for Adverse Events (CTCAE) version 5.0 or above. Cytokine release syndrome (CRS) will be graded using a revised CRS grading scale developed by Lee et al (Lee et al, 2014). The incidence and severity of CRS will be reported as a syndrome with severity per Lee et al. Individual symptoms associated with CRS will be graded per CTCAE version 5.0 or above.

Tables and/or narratives of deaths through the long term follow-up and treatment related SAEs will be provided.

Subjects enrolled but not dosed with axicabtagene ciloleucel will be followed for adverse events for 30 days after the last study procedure. Adverse events reported in these patients will be archived in the study database and available in SDTM and ADaM datasets, but will not be tabulated in adverse event summaries.

The safety summaries by disease diagnosis type (eg, double-hit lymphomas vs triple-hit lymphomas vs non double-/triple-hit with IPI score ≥ 3) may be presented.

9.6.1. Adverse Events

The subject incidence of the following treatment-emergent adverse events will be tabulated by SOC and PT:

- Summary of adverse events (any, worst severity, serious, related)
- All adverse events
- All serious adverse events
- All leukapheresis-related adverse events
- All conditioning-chemotherapy-related adverse events
- All axicabtagene ciloleucel-related adverse events
- All conditioning chemotherapy-related serious adverse events
- All axicabtagene ciloleucel-related serious adverse events
- All Grade 3 or higher adverse events

- All Grade 3 or higher conditioning chemotherapy-related adverse events
- All Grade 3 or higher axicabtagene ciloleucel-related adverse events
- The most common adverse events (incidence ≥20%)
- The most common grade 3 or higher adverse events (incidence ≥10%)
- Fatal adverse events
- Adverse events of interest, including important identified and important potential risks

Summary statistics for the onset time (Kaplan-Meier estimates) and duration of adverse events of interest will be provided.

The subject incidence of deaths will be provided.

A subject listing of deaths and serious adverse events (including narratives) will be provided.

Subgroup analyses of AEs may be generated using the covariates listed in Section 4.2.

Summary of AEs during specific time periods may be conducted to support the safety evaluations.

9.6.1.1. Exposure to Study Treatment

Summary statistics and subject listings will be provided for the following:

- Total body surface area (BSA)-adjusted dose of cyclophosphamide
- Total BSA-adjusted dose of fludarabine
- Weight-adjusted dose of axicabtagene ciloleucel
- Number and percent of subjects who received a dose of axicabtagene ciloleucel within +/- 10% of the planned dose

The analysis by patient demographics (age, and sex etc.) as well as by tumor burden may be provided.

Separate summaries will be presented for the 2nd administration of conditioning chemotherapy and retreatment of axicabtagene ciloleucel for subjects in the Safety Retreatment Analysis Set.

9.6.1.2. Exposure to Concomitant Medications and Procedures

The subject incidence of concomitant medications will be provided and summarized, including tocilizumab, steroids, vasopressors, and IVIG. In addition, the incidence of dialysis and intubation will be summarized.

9.6.1.3. Schedule of Study Treatment

Summary statistics will be provided for the following durations:

- Days from leukapheresis to commencement of conditioning chemotherapy
- Days from leukapheresis to administration of axicabtagene ciloleucel
- Days from leukapheresis to axicabtagene ciloleucel product release
- Days from leukapheresis to receipt of axicabtagene ciloleucel at the study site
- Days from conditioning chemotherapy to administration of axicabtagene ciloleucel
- Duration of hospitalization for the axicabtagene ciloleucel infusion

9.7. Pharmacokinetics

Summary statistics for the level of CAR T cells in serum post axicabtagene ciloleucel infusion will be provided for CAR T cells measured at day 7, week 2, week 4, month 3, month 6, month 12, and month 24. The maximum CAR T cell level attained, the time at which the maximum level was attained, and the time at which there were no detectable CAR T cells in the serum will be summarized. The area under the curve (AUC) of CAR T cell levels from day 0 to day 28 and the peak value of CAR T cell levels from day 0 to day 28 will be summarized and may be used in subgroup analyses. Additional detail can be found in the translational research plan.

9.8. Pharmacodynamics

Levels of cytokines in serum in relationship with clinical outcome will be detailed in the translational research plan.

9.9. Subsequent Anti-Cancer Therapy and SCT

The incidence and type (by WHO Drug coded term and categories) of subsequent anti-lymphoma therapy and subsequent SCT (autologous, allogeneic) by treatment period will be summarized.

The subject incidence of SCT post-treatment with axicabtagene ciloleucel will be tabulated by occurrence of SCT post progression on axicabtagene ciloleucel or occurrence of SCT while in an axicabtagene ciloleucel-induced response.

10. CHANGES FROM PROTOCOL-SPECIFIED ANALYSES

There is no change from protocol-specified analyses.

11. REFERENCES

Casasnovas RO, Ysebaert L, Thieblemont C, Bachy E, Feugier P, Delmer A, et al. FDG-PET-driven consolidation strategy in diffuse large B-cell lymphoma: final results of a randomized phase 2 study. Blood. 2017;130(11):1315-26.

Cheson BD, Fisher RI, Barrington SF, Cavalli F, Schwartz LH, Zucca E, Lister TA. Recommendations for initial evaluation, staging, and response assessment of Hodgkin and non-Hodgkin lymphoma: the Lugano classification. J Clin Oncol. 2014;32(27):3059-68.

Lee DW, Gardner R, Porter DL, Louis CU, Ahmed N, Jensen M, Grupp SA, Mackall CL. Current concepts in the diagnosis and management of cytokine release syndrome. Blood. 2014;124(2):188-95.

Topp MS, Gökbuget N, Stein AS, Zugmaier G, O'Brien S, Bargou RC, et al. Safety and activity of blinatumomab for adult patients with relapsed or refractory B-precursor acute lymphoblastic leukaemia: a multicentre, single-arm, phase 2 study. Lancet Oncology. 2015;16(1):57-66.

12. APPENDICES

Appendix 1. Conventions for Clinical Data that Require Imputation for Partial or Missing Dates
Appendix 2. Derivation of Time to Event Endpoints and Last Date Known to Be Alive

Appendix 1. Conventions for Clinical Data that Require Imputation for Partial or Missing Dates

The following data will be imputed using the following algorithm:

- Adverse event start dates
- Deaths (please see exceptions below)
- Concomitant medication start dates
- Subsequent anti-cancer therapy start dates

Table 2. Imputation Rules for Partial or Missing Start Dates

<table>
<tr><th colspan="2" rowspan="3">Start Date</th><th colspan="7">Stop Date</th></tr>
<tr><th colspan="2">Complete: yyyymmdd</th><th colspan="2">Partial: yyyymm</th><th colspan="2">Partial: yyyy</th><th rowspan="2">Missing</th></tr>
<tr><th>< Study Day 0</th><th>≥ Study Day 0</th><th>< Study Day 0 yyyymm</th><th>≥ Study Day 0 yyyymm</th><th>< Study Day 0 yyyy</th><th>≥ Study Day 0 yyyy</th></tr>
<tr><td rowspan="2">Partial yyyymm</td><td>= Study Day 0 yyyymm</td><td rowspan="2">2</td><td>1</td><td rowspan="2">2</td><td>1</td><td>n/a</td><td>1</td><td>1</td></tr>
<tr><td>≠ day 0 yyyymm</td><td>2</td><td>2</td><td>2</td><td>2</td><td>2</td></tr>
<tr><td colspan="9"></td></tr>
<tr><td rowspan="2">Partial yyyy</td><td>= Study Day 0 yyyy</td><td rowspan="2">3</td><td>1</td><td rowspan="2">3</td><td>1</td><td>n/a</td><td>1</td><td>1</td></tr>
<tr><td>≠ Study Day 0 yyyy</td><td>3</td><td>3</td><td>3</td><td>3</td><td>3</td></tr>
<tr><td colspan="9"></td></tr>
<tr><td colspan="2">Missing</td><td>4</td><td>1</td><td>4</td><td>1</td><td>4</td><td>1</td><td>1</td></tr>
</table>

1 = impute the date of day 1
2 = impute the first of the month
3 = impute January 1 of the year
4 = impute January 1 of the stop year
Note: if the start date imputation leads to a start date that is after the stop date, then do not impute the start date.

Imputation rules for partial or missing death dates:

1) If death year and month are available but day is missing:

   - If yyyymm for the last contact date = yyyymm for death date, set death date to the day after the last date known to be alive.

   - If yyyymm for the last date known to be alive < yyyymm for death date, set death date to the first day of the death month.

   - If yyyymm for last date known to be alive > yyyymm for death date, data error and do not impute.

2) If both month and day are missing for death date or a death date is completely missing, do not impute and censor the subject survival time at the last date known to be alive.

Appendix 2. Derivation of Time to Event Endpoints and Last Date Known to Be Alive

Additional detail on the derivations of DOR, DORR, EFS, PFS, and OS is provided below.

Duration of response (DOR): DOR is defined only for subjects who experience an objective response (CR or PR) after axicabtagene ciloleucel infusion and is the time from the first objective response to disease progression per the Lugano Classification (Cheson et al, 2014) or death due to any cause. Subjects not meeting the criteria for disease progression or death by the analysis data cutoff date will be censored at their last evaluable disease assessment date. The DOR for subjects who receive any subsequent anti-lymphoma therapy (including SCT or retreatment with axicabtagene ciloleucel) in the absence of documented disease progression or death due to any cause will be censored at the last evaluable disease assessment prior to the start date of the subsequent anti-lymphoma therapy. A sensitivity analysis will be conducted in which disease assessments obtained after SCT are included in the derivation of DOR.

Primary analysis of DOR:

Circumstance	Event / Censored	Date of Event / Censoring
Disease progression prior to initiation of subsequent anti-lymphoma therapy (including SCT or retreatment with axicabtagene ciloleucel)	Event	Progression date
Death due to any cause without documented disease progression and without subsequent anti-lymphoma therapy (including SCT or retreatment with axicabtagene ciloleucel)	Event	Death date
Remain in response without any subsequent anti-lymphoma therapy (including SCT or retreatment with axicabtagene ciloleucel)	Censored	Date of last evaluable disease assessment
Initiated subsequent anti-lymphoma therapy (including SCT or retreatment with axicabtagene ciloleucel) prior to documented progression or death due to any cause	Censored	Date of last evaluable disease assessment prior to initiation of subsequent anti-lymphoma therapy (including SCT or retreatment, whichever is earlier)
Progression or death due to any cause documented after data cutoff for analysis	Censored	Date of last evaluable disease assessment prior to data cutoff for analysis
No progression through discontinuation of study prior to the data cutoff for analysis	Censored	Date of last evaluable disease assessment prior to data cutoff for analysis

Sensitivity analysis of DOR (including the disease assessments after SCT):

Circumstance	Event / Censored	Date of Event / Censoring
Disease progression after SCT, but prior to other subsequent anti-lymphoma therapy (including retreatment with axicabtagene ciloleucel)	Event	Progression date
Death due to any cause after SCT without prior documented disease progression and other subsequent anti-lymphoma therapy (including retreatment with axicabtagene ciloleucel)	Event	Death date
Remain in response after SCT without other subsequent anti-lymphoma therapy (including retreatment with axicabtagene ciloleucel)	Censored	Date of last evaluable disease assessment
Remain in response after SCT prior to other subsequent anti-lymphoma therapy (including retreatment with axicabtagene ciloleucel)	Censored	Date of last evaluable disease assessment prior to initiation of subsequent anti-lymphoma therapy or retreatment, whichever is earlier
Progression or death due to any cause documented after data cutoff for analysis	Censored	Date of last evaluable disease assessment prior to data cutoff for analysis
No progression through discontinuation of study prior to the data cutoff for analysis	Censored	Date of last evaluable disease assessment prior to data cutoff for analysis

Duration of response to retreatment (DORR): DORR is defined only for subjects who receive retreatment following progression of disease and then go on to experience an objective response to retreatment. It is defined as the time from the first objective response after retreatment to disease progression per the Lugano Classification (Cheson et al, 2014) or death due to any cause. Subjects not meeting the criteria for disease progression after retreatment or death due to any cause by the analysis data cutoff date will be censored at their last evaluable disease assessment date after retreatment. The DORR for subjects who receive any subsequent anti-lymphoma therapy (including SCT) after axicabtagene ciloleucel retreatment in the absence of prior documented progression will be censored at the last evaluable disease assessment prior to subsequent anti-lymphoma therapy. Disease assessments obtained after SCT will not be used in the derivation of DORR. A sensitivity analysis may be conducted in which disease assessments obtained after the SCT are included in the derivation of DORR.

Primary analysis of DORR:

Circumstance	Event / Censored	Date of Event / Censoring
Disease progression prior to initiation of subsequent anti-lymphoma therapy (including SCT)	Event	Progression date
Death due to any cause without documented disease progression and without subsequent anti-lymphoma therapy (including SCT)	Event	Death date
Remain in response without any subsequent anti-lymphoma therapy (including SCT)	Censored	Date of last evaluable disease assessment
Initiated subsequent anti-lymphoma therapy (including SCT) prior to documented progression or death due to any cause	Censored	Date of last evaluable disease assessment prior to initiation of subsequent anti-lymphoma therapy (including SCT)
Progression or death due to any cause documented after data cutoff for analysis	Censored	Date of last evaluable disease assessment prior to data cutoff for analysis
No progression through discontinuation of study prior to the data cutoff for analysis	Censored	Date of last evaluable disease assessment prior to data cutoff for analysis

Sensitivity analysis of DORR (including the disease assessments after SCT):

Circumstance	Event / Censored	Date of Event / Censoring
Disease progression after SCT, but prior to other subsequent anti-lymphoma therapy	Event	Progression date
Death due to any cause after SCT without prior documented disease progression and other subsequent anti-lymphoma therapy	Event	Death date
Remain in response after SCT without other subsequent anti-lymphoma therapy	Censored	Date of last evaluable disease assessment
Remain in response after SCT prior to other subsequent anti-lymphoma therapy	Censored	Date of last evaluable disease assessment prior to initiation of subsequent anti-lymphoma therapy
Progression or death due to any cause documented after data cutoff for analysis	Censored	Date of last evaluable disease assessment prior to data cutoff for analysis
No progression through discontinuation of study prior to the data cutoff for analysis	Censored	Date of last evaluable disease assessment prior to data cutoff for analysis

Event-free Survival (EFS): EFS is defined as the time from the axicabtagene ciloleucel infusion date to the earliest date of disease progression per the Lugano Classification (Cheson et al, 2014), commencement of subsequent anti-lymphoma therapy including SCT or retreatment with axicabtagene ciloleucel, or death from any cause. Subjects alive, in response, and with no new anti-lymphoma therapy including SCT or retreatment with axicabtagene ciloleucel will be censored at the last evaluable disease assessment.

Primary analysis of EFS:

Circumstance	Event / Censored	Date of Event / Censoring
Disease progression prior to initiation of subsequent anti-lymphoma therapy (including SCT or retreatment with axicabtagene ciloleucel)	Event	Progression date
Subject with CR or PR or a best response of SD and subsequently received anti-lymphoma therapy (including SCT or retreatment with axicabtagene ciloleucel)	Event	Date of last evaluable disease assessment prior to initiation of subsequent anti-lymphoma therapy (including SCT or retreatment)
Subsequent anti-lymphoma therapy in the absence of any evaluable disease assessment	Event	Axicabtagene ciloleucel infusion date
Death due to any cause without documented disease progression (excluding the death after the initiation of subsequent anti-lymphoma therapy)	Event	Death date
Death due to any cause without documented disease progression and after the initiation of subsequent anti-lymphoma therapy (including SCT or retreatment with axicabtagene ciloleucel)	Event	Date of last evaluable disease assessment prior to initiation of subsequent anti-lymphoma therapy (including SCT or retreatment)
Subsequent anti-lymphoma therapy (including SCT or retreatment with axicabtagene ciloleucel) started prior to documented progression or death due to any cause	Event	Date of last evaluable disease assessment prior to initiation of subsequent anti-lymphoma therapy (including SCT or retreatment)
Remain in response without new anti-lymphoma therapy (including SCT or retreatment with axicabtagene ciloleucel)	Censored	Date of last evaluable disease assessment
Progression or death documented after data cutoff for analysis	Censored	Date of last evaluable disease assessment prior to data cutoff for analysis
Remain event-free through the discontinuation of the study prior to data cutoff for analysis	Censored	Date of last evaluable disease assessment prior to data cutoff for analysis
Subjects treated but no post baseline disease assessment	Censored	Axicabtagene ciloleucel infusion date

Progression-free Survival (PFS): PFS is defined as the time from the axicabtagene ciloleucel infusion date to the date of disease progression per the Lugano Classification (Cheson et al, 2014) or death due to any cause. Subjects not meeting the criteria for disease progression or death will be censored at the last evaluable disease assessment. Subjects who receive any subsequent anti-lymphoma therapy including SCT or retreatment with axicabtagene ciloleucel in the absence of documented disease progression or death due to any cause will be censored at the last evaluable disease assessment prior to the start date of the subsequent anti-lymphoma therapy.

Primary analysis of PFS:

Circumstance	Event / Censored	Date of Event / Censoring
Disease progression prior to initiation of subsequent anti-lymphoma therapy (including SCT or retreatment with axicabtagene ciloleucel)	Event	Progression date
Death due to any cause without documented disease progression (excluding the death after the initiation of subsequent anti-lymphoma therapy (including SCT or retreatment with axicabtagene ciloleucel)	Event	Death date
Remain in response without subsequent anti-lymphoma therapy (including SCT or retreatment with axicabtagene ciloleucel)	Censored	Date of last evaluable disease assessment
Initiated subsequent anti-lymphoma therapy (including SCT or retreatment with axicabtagene ciloleucel) prior to documented progression or death due to any cause	Censored	Date of last evaluable disease assessment prior to initiation of subsequent anti-lymphoma therapy (including SCT or retreatment)
Progression or death due to any cause documented after data cutoff for analysis	Censored	Date of last evaluable disease assessment prior to data cutoff for analysis
No progression through discontinuation of study prior to the data cutoff for analysis	Censored	Date of last evaluable disease assessment prior to data cutoff for analysis
Subjects treated but no post baseline disease assessment	Censored	Axicabtagene ciloleucel infusion date

Overall Survival (OS): OS is defined as the time from the axicabtagene ciloleucel infusion to the date of death due to any cause. Subjects who have not died by the analysis data cutoff date will be censored at their last date known to be alive prior to the data cutoff date with the

exception that subjects known to be alive or determined to have died after the data cutoff date will be censored at the data cutoff date.

Circumstance	Event / Censored	Date of Event / Censoring
Death prior to data cutoff date for analysis	Event	Death date
Death after data cutoff date for analysis	Censored	Data cutoff date
Known to be alive after data cutoff date for analysis	Censored	Data cutoff date
Alive up through data cutoff date and no further information available after cutoff date	Censored	Last date known to be alive up through the data cutoff date
Alive including withdrawal of consent or lost to follow-up prior to data cutoff date	Censored	Last date known to be alive prior to withdrawal of consent or lost to follow-up

Last date known to be alive

The last date known to be alive with be derived by obtaining the maximum complete date among the following data modules:

- Start and end dates of AE (including targeted AE) and concomitant medication
- Screening dates
- Leukapheresis dates
- Conditioning chemo admin dates
- axicabtagene ciloleucel infusion dates
- CT scan dates
- PET scan dates
- Clinical symptoms of lymphoma assessment dates
- Target lesion assessment
- Non-target lesion assessment
- New lesion assessment

- Disease response assessment
- Long term follow up subject status date where status = ‘alive’
- End of treatment disposition where status is not equal to death, lost to follow up
- End of post-treatment assessment period where status is not equal to death, lost to follow up
- End of study data where end of study reason is not equal to death, lost to follow up
