# Supplementary material for: Axicabtagene ciloleucel as first-line therapy in high-risk large B-cell lymphoma: the phase 2 ZUMA-12 trial
Source: Nat Med. 2022 Mar 21;28(4):735–42. doi: 10.1038/s41591-022-01731-4 (PMC9018426; doi:10.1038/s41591-022-01731-4)
Supplement: Supplementary file 2 — Reporting Summary [file 41591_2022_1731_MOESM2_ESM.pdf]

nature portfolio

Corresponding author(s): Sattva S Neelapu, MD

Last updated by author(s): Jan 31, 2022

Reporting Summary

Nature Portfolio wishes to improve the reproducibility of the work that we publish. This form provides structure for consistency and transparency in reporting. For further information on Nature Portfolio policies, see our Editorial Policies and the Editorial Policy Checklist.

Statistics

For all statistical analyses, confirm that the following items are present in the figure legend, table legend, main text, or Methods section.

n/a	Confirmed	
☐	☒	The exact sample size (*n*) for each experimental group/condition, given as a discrete number and unit of measurement
☒	☐	A statement on whether measurements were taken from distinct samples or whether the same sample was measured repeatedly
☐	☒	The statistical test(s) used AND whether they are one- or two-sided<br>*Only common tests should be described solely by name; describe more complex techniques in the Methods section.*
☐	☒	A description of all covariates tested
☒	☐	A description of any assumptions or corrections, such as tests of normality and adjustment for multiple comparisons
☐	☒	A full description of the statistical parameters including central tendency (e.g. means) or other basic estimates (e.g. regression coefficient) AND variation (e.g. standard deviation) or associated estimates of uncertainty (e.g. confidence intervals)
☐	☒	For null hypothesis testing, the test statistic (e.g. *F*, *t*, *r*) with confidence intervals, effect sizes, degrees of freedom and *P* value noted<br>*Give P values as exact values whenever suitable.*
☒	☐	For Bayesian analysis, information on the choice of priors and Markov chain Monte Carlo settings
☒	☐	For hierarchical and complex designs, identification of the appropriate level for tests and full reporting of outcomes
☒	☐	Estimates of effect sizes (e.g. Cohen's *d*, Pearson's *r*), indicating how they were calculated

Our web collection on statistics for biologists contains articles on many of the points above.

Software and code

Policy information about availability of computer code

Data collection	No software was used for data collection.
Data analysis	All data was analyzed using SAS software (version 9.4).

For manuscripts utilizing custom algorithms or software that are central to the research but not yet described in published literature, software must be made available to editors and reviewers. We strongly encourage code deposition in a community repository (e.g. GitHub). See the Nature Portfolio guidelines for submitting code & software for further information.

Data

Policy information about availability of data

All manuscripts must include a data availability statement. This statement should provide the following information, where applicable:

- Accession codes, unique identifiers, or web links for publicly available datasets
- A description of any restrictions on data availability
- For clinical datasets or third party data, please ensure that the statement adheres to our policy

Gilead is committed to sharing clinical trial data with external medical experts and scientific researchers in the interest of advancing public health. As such, Gilead shares anonymized individual patient data (IPD) upon request or as required by law and/or regulation. Qualified external researchers may request IPD for studies of Gilead compounds approved in the United States and the European Union with a marketing authorization date on or after 1 January 2014 and are publicly listed on clinicaltrials.gov or the European Union-Clinical Trials Register (EU CTR). For studies of newly approved compounds or indication, the IPD will be available for request six months after US Food and Drug Administration (FDA) and European Medicines Agency (EMA) approval. Such requests are at Gilead's discretion and are dependent on the nature of the request, the merit of the research proposed, availability of the data, and the intended use of the data. If Gilead agrees to the

release of clinical data for research purposes, the requestor will be required to sign a data sharing agreement to ensure protection of patient confidentiality before the release of any data. Please contact medinfo@kitepharma.com for all data requests.

Field-specific reporting

Please select the one below that is the best fit for your research. If you are not sure, read the appropriate sections before making your selection.

☒ Life sciences ☐ Behavioural & social sciences ☐ Ecological, evolutionary & environmental sciences

For a reference copy of the document with all sections, see nature.com/documents/nr-reporting-summary-flat.pdf

Life sciences study design

All studies must disclose on these points even when the disclosure is negative.

Sample size	Although there is no formal hypothesis testing in ZUMA-12, the trial used a single-arm design to estimate the CR rate in patients with high-risk large B-cell lymphoma treated with axi-cel. A CR rate of 60% with axi-cel treatment was targeted. With a total sample size of 40 patients, an observed CR rate of 60% would yield an 80% CI for the response rate with a maximum half-width of less than or equal to 11%, corresponding to a lower limit of at least 48.6%. This target CR rate, and the lower limit of the 80% CI for the CR rate, is meaningful because it would represent a significant improvement in the response rate for the subjects with high-risk large B-cell lymphoma and would likely offer an improvement over existing therapies in patients with high-risk large B-cell lymphoma, higher than the primary objective in the GELA phase 2 study of achieving a higher than 50% CR rate after 4 cycles of induction regimes of R-ACVBP or R-CHOP-14 (Casasnovas et al, 2017).
Data exclusions	No primary or secondary endpoint data were excluded from the analysis.
Replication	The study protocol is sufficient to use in replicating the trial in the future, if necessary. Replication within the results in study was not applicable due to the inclusion of human participants with disease characteristics that are not common in the population, and the descriptive nature of the analysis in which no formal hypothesis testing was performed.
Randomization	ZUMA-12 used a single-arm trial design, thus randomization of patients is not relevant to the study.
Blinding	ZUMA-12 used an open label, single-arm trial design, thus blinding is not relevant to the study.

Reporting for specific materials, systems and methods

We require information from authors about some types of materials, experimental systems and methods used in many studies. Here, indicate whether each material, system or method listed is relevant to your study. If you are not sure if a list item applies to your research, read the appropriate section before selecting a response.

Materials & experimental systems

n/a	Involved in the study	
☒	☐	Antibodies
☒	☐	Eukaryotic cell lines
☒	☐	Palaeontology and archaeology
☒	☐	Animals and other organisms
☐	☒	Human research participants
☐	☒	Clinical data
☒	☐	Dual use research of concern

Methods

n/a	Involved in the study	
☒	☐	ChIP-seq
☐	☒	Flow cytometry
☒	☐	MRI-based neuroimaging

Human research participants

Policy information about studies involving human research participants

Population characteristics	The following pre-specified covariates at screening/baseline or on the study were reported in CR and ORR subgroups analyses: age (<65, ≥65 years), sex, IPI score and/or double- or triple-hit status per central laboratory or per investigator, double expression per central or per investigator, c-Myc expression per central laboratory or per investigator, ECOG status, Deauville five-point scale of 4 or 5, best response to prior therapy, and cell of origin.
Recruitment	Recruitment of patients for the study was done by the investigators at each site. Enrollment was open to eligible patients and manufacturing slots for axi-cel were available for eligible patients at enrollment. Although recruitment biases cannot be ruled out, it would be difficult to identify and collect the sources of bias and assess the impact on the results in this study.
Ethics oversight	The protocol was approved by the institutional review board or independent ethics committee at each study site and was provided to the key sponsor contact. These institutions are City of Hope National Medical Center, Moffitt Cancer Center, MD Anderson Cancer Center, Banner MD Anderson Cancer Center, Vanderbilt - Ingram Cancer Center, Peter MacCallum Cancer Center, and Hopital Saint-Louis (AP-HP) - Service Hematologic Seniors. Patients are not compensated for trial participation

beyond receipt of therapy and associated care. Patients may be compensated for study-related illness or injury pursuant to the information outlined in the injury section of the informed consent form.

Note that full information on the approval of the study protocol must also be provided in the manuscript.

Clinical data

Policy information about clinical studies

All manuscripts should comply with the ICMJE guidelines for publication of clinical research and a completed CONSORT checklist must be included with all submissions.

Clinical trial registration	ClinicalTrials.gov Number NCT03761056
Study protocol	The full redacted protocol as well as the SAP are included as Supplementary Information.
Data collection	Study accrual occurred between February 6, 2019 and October 22, 2020. The investigator and study staff of each trial site were responsible for maintaining a comprehensive and centralized filing system of all patient records that are readily retrieved to be monitored and or audited at any time by the key sponsor contact, health authorities, and IRB/IECs. All data were collected in an electronic case report form (CRF) system between study initiation on January 29, 2019 and the data cutoff date for the primary analysis of May 17, 2021.
Outcomes	All primary and secondary outcomes were pre-defined in the approved study protocol. The primary endpoint was complete response rate per the Lugano Classification as determined by study investigators. Secondary endpoints were objective response rate (defined as the incidence of either a complete response or a partial response), duration of response (among patients experiencing an objective response, defined as the time from the first objective response to events of disease progression or death from any cause), event-free survival (defined as the time from the axi-cel infusion date to the earliest date of the events of disease progression, commencement of subsequent new anti lymphoma therapy, including stem cell transplant, or death from any cause), progression-free survival (defined as the time from the axi-cel infusion date to the date of the events of disease progression or death from any cause), overall survival (defined as the time from axi-cel infusion to the date of the event of death from any cause), incidence of adverse events, levels of anti-CD19 CAR T cells in blood, levels of cytokines in serum, and associations between biomarker levels and clinical outcomes.

Flow Cytometry

Plots

Confirm that:

☒ The axis labels state the marker and fluorochrome used (e.g. CD4-FITC).

☒ The axis scales are clearly visible. Include numbers along axes only for bottom left plot of group (a 'group' is an analysis of identical markers).

☒ All plots are contour plots with outliers or pseudocolor plots.

☒ A numerical value for number of cells or percentage (with statistics) is provided.

Methodology

Sample preparation	Transduced CD19 CAR T cells obtained on harvest day were cryopreserved and stored for later analysis (ex. flow, qPCR). Cryopreserved CD19 CAR T cells were thawed in cell culture media (RPMI 1640 + FBS, 10% v/v), strained through a 40µm filter, and washed. Cells were then transferred to cell stain buffer for antibody staining and flow analysis.
Instrument	BD FACS Canto/Canto II 6-10 color flow cytometer, with 3 lasers: 488 nm Blue laser, 633 nm Red laser, 405 nm violet laser
Software	BD FACSDivaTM v.8.0<br>FlowJo v. 10, Data Analysis Software for Flow Cytometry
Cell population abundance	Flow analysis was performed on CD19 CAR T cells post manufacturing therefore samples contain mostly CD3+ T cells. Within the CD19 CAR T cell samples analyzed, the abundance of T cells being interrogated by the flow panel was high.
Gating strategy	T-cell phenotypes were assessed for CCR7 and CD45RA expression by multicolor flow cytometry using established protocols and antibodies. The analysis employed a cell-gating strategy that selected viable CD3+ cells by excluding dead/apoptotic cells using 7-AAD. Data analysis was performed using FlowJo software, version 10 (FLOWJO) using standardized gating and compensation strategies. For further details see list of cell staining reagents below and gating strategy supplementary figure.<br><br>BD Biosciences APC-H7 – human CD8 Clone SK1, Cat# 641400<br>BioLegend FITC - human CD197 (CCR7) Clone G043H7, Cat# 353216<br>BioLegend BV421 - human CD3 Clone SK7, Cat# 344834<br>BioLegend APC - human CD45RA Clone HI100, Cat# 304112<br>BioLegend Cell Staining Buffer, Cat# 420201<br>BD Biosciences 7-AAD cellular viability stain, Cat# 559925<br>Miltenyi Biotec FcR Blocking Reagent (human), Cat# 130-059-901

☒ Tick this box to confirm that a figure exemplifying the gating strategy is provided in the Supplementary Information.
